# Rhoifolin Alleviates Alcoholic Liver Disease *In Vivo* and *In Vitro via* Inhibition of the TLR4/NF-κB Signaling Pathway

**DOI:** 10.3389/fphar.2022.878898

**Published:** 2022-05-24

**Authors:** Baoyu Mai, Ling Han, Jiarui Zhong, Jingqi Shu, Zelin Cao, Jiaqi Fang, Xiaoying Zhang, Zelin Gao, Fengxia Xiao

**Affiliations:** ^1^ School of Pharmaceutical Sciences, Guangzhou University of Chinese Medicine, Guangzhou, China; ^2^ State Key Laboratory of Dampness Syndrome of Chinese Medicine, The Second Affiliated Hospital of Guangzhou University of Chinese Medicine (Guangdong Provincial Hospital of Chinese Medicine), Guangzhou, China; ^3^ Guangdong Provincial Key Laboratory of Clinical Research on Traditional Chinese Medicine Syndrome, Guangzhou, China; ^4^ Guangdong Provincial Clinical Medicine Research Center for Chinese Medicine Dermatology, Guangzhou, China; ^5^ College of Acumox and Tuina, Jiangxi University of Chinese Medicine, Jiangxi, China; ^6^ Jiangsu Hengrui Medicine Co., Ltd., Jiangsu, China; ^7^ Maoming Branch, Guangdong Laboratory for Lingnan Modern Agriculture, Maoming, China

**Keywords:** rhoifolin, ALD, LO2 cell, NF-κB, CYP2E1

## Abstract

**Background:** Alcoholic liver disease (ALD) is a common chronic liver disorder worldwide, which is detrimental to human health. A preliminary study showed that the total flavonoids within *Citrus grandis* “Tomentosa” exerted a remarkable effect on the treatment of experimental ALD. However, the active substances of *Citrus grandis* “Tomentosa” were not elucidated. Rhoifolin (ROF) is a flavonoid component present in high levels. Therefore, this research aimed to evaluate the hepatoprotective effects of ROF and its possible mechanisms.

**Methods:** Molecular docking was performed to analyze the binding energy of ROF to the main target proteins related to ALD. Subsequently, mice were fed ethanol (ETH) for 49 days to establish the chronic alcoholic liver injury models. The liver pathological injury, serum aminotransferase levels, and oxidative stress levels in the liver tissue were measured. Human normal hepatocytes (LO2 cells) were incubated with ETH to construct the alcoholic liver cell model. The inflammatory markers and apoptosis factors were evaluated using real-time PCR and flow cytometry. Finally, the effects of ROF on the CYP2E1 and NF-κB signaling pathways were tested *in vitro* and *in vivo*.

**Results:** Molecular docking results demonstrated that ROF was able to successfully dock with the target proteins associated with ALD. In animal studies, ROF attenuated ETH-induced liver damage in mice by decreasing the serum concentrations of AST and ALT, reducing the expression of inflammatory cytokines, and maintaining antioxidant balance in the liver tissue. The *in vitro* experiments demonstrated that ROF suppressed ETH-induced apoptosis in LO2 cells by promoting Bcl-2 mRNA and inhibiting Bax mRNA and caspase 3 protein expression. ROF decreased the level of LDH, ALT, AST, ROS, and MDA in the supernatant; induced the activity of GSH and SOD; and inhibited TNF-α, IL-6, and IL-1β expression levels. Mechanistically, ROF could significantly downregulate the expression levels of CYP2E1, TLR4, and NF-κB phosphorylation.

**Conclusion:** This study indicates that ROF is the active component within the total flavonoids, which may alleviate ETH-induced liver injury by inhibiting NF-κB phosphorylation. Therefore, ROF may serve as a promising compound for treating ALD.

## Introduction

Alcoholic liver disease (ALD) refers to a spectrum of alcohol-related liver injuries due to excess alcohol intake, which includes three stages of liver damage: steatosis, hepatitis, and fibrosis/cirrhosis (Liu et al., 2019; Roychowdhury et al., 2019). The prevalence of ALD is increasing worldwide and undermines the public health response (Wang and Liu, 2021). Many factors have contributed to the occurrence of ALD. Growing evidence has indicated that oxidative stress is involved in the pathogenesis of alcoholic liver damage (Wang et al., 2016). Excess alcohol intake can generate various harmful free radicals and acetaldehyde, an extremely toxic metabolite which alters enzyme activity and destabilizes the cellular oxidant and antioxidant defense systems (Wang et al., 2017; Lu and Cederbaum, 2018). In addition, the development and progression of ALD are closely associated with inflammatory responses (Wang et al., 2020). Alcohol abuse can trigger the formation of endotoxins and activate Kupffer cells, thereby generating the pro-inflammatory factors tumor necrosis factor-alpha (TNF-α) and interleukin-6 (IL-6) (Yang et al., 2020). This may lead to oxidative damage, hepatocyte necrosis, and inflammatory cell infiltration (Song et al., 2021). Glucocorticoids and hepatic protectants have been proven, by the FDA, to be effective for treating ALD patients (Purohit et al., 2007; Yang et al., 2020), but the debate on their effectiveness continues (Kharbanda, 2013). Over the last decade, herbal medicine has gained increasing attention as a potential agent for the treatment and prevention of ALD, owing to its multitarget activities and fewer adverse effects. Therefore, it is necessary to develop a safe and effective drug for ALD, and herbal medicines provide a valuable avenue for exploration.

According to folk therapy, *Citrus grandis* “Tomentosa” has been traditionally used to wake a drunk person up (Xiao et al., 2012b). The tea made from *Citrus grandis* “Tomentosa” has become increasingly important for wine consumers, due to its anti-hangover effects. This research group has previously observed the therapeutic effect of total flavones from *Citrus grandis* “Tomentosa” in ALD (Xiao et al., 2012a; Rodriguez et al., 2018). However, the active substances are still not clear, and further investigations need to be conducted. Therefore, we studied the chemical composition of the total flavonoids, and rhoifolin (ROF) was one of the pharmacodynamic compounds present in a higher level. ROF possesses anti-inflammatory and antioxidant properties (Liao et al., 2019; Peng et al., 2020). Our previous work (Fang et al., 2020) showed that ROF attenuated inflammation *in vitro* and *in vivo* by mediating the nuclear factor-kappa B (NF-κB) pathway. However, its effects and mechanisms in ALD have yet to be elucidated.

In this study, the therapeutic mechanism of ROF was investigated via molecular docking studies and experimental validation. At first, the target docking of ROF with related proteins in ALD was established through molecular docking and network analysis. Then, ETH-induced mice and human normal hepatocytes (LO2 cells) were selected as experimental models to verify the effects of these predicted targets on ALD. The results showed that ROF attenuated ETH-induced liver damage both *in vitro* and *in vivo* and suppressed liver inflammation and oxidative stress. Furthermore, we also discussed the preliminary findings regarding the ROF molecular mechanism. This work provides experimental evidence for expanding the clinical applications of ROF in ALD and other liver diseases.

## Methodology

### Materials

ROF was extracted from *Citrus grandis* “Tomentosa.” The HPLC method (Lin, 2014) was used for purification, and the purity of ROF was > 98% (Supplementary Figure S1). Ethanol (ETH, Catalog No: 181130) was purchased from Tianjin Guangcheng Chemical Reagent Co., Ltd. Bifendatatum (BIF, Catalog No: 170911) was purchased from Beijing Union Pharmaceutical Factory. The aspartate aminotransferase (AST, Catalog No: C010-2-1), alanine aminotransferase (ALT, Catalog No: C009-2-1), lactate dehydrogenase (LDH, Catalog No: A020-2), malondialdehyde (MDA, Catalog No: A003-1), glutathione (GSH, Catalog No: A006-2-1), superoxide dismutase (SOD, Catalog No: A001-3), and DCFHDA (Catalog No: E004-1-1) biochemical assay kits were purchased from Nanjing Jiancheng Bioengineering Institute. BCA protein assay reagent kits (Catalog No: P0010) were purchased from Shanghai Beyotime Biotechnology. TNF-α (Catalog No: MM-0132M2), IL-6 (Catalog No: MM-0163M2), and IL-1β (Catalog No: MM-0040M2) were procured from Jiangsu Meimian industrial Co. Ltd. (Meimian, Yancheng, Jiangsu, China). The primary antibodies including Polyclonal antibodies cytochrome P450 2E1 (CYP2E1, Catalog No: DF6883), caspase 3 (Catalog No: AF6311), cleaved caspase 3 (Catalog No: AF70221), toll-like receptor 4 (TLR4, Catalog No: AF7017), NOD-like receptor protein 3 (NLRP3, Catalog No: DF7438), tubulin beta (Catalog No: AF7011), and GAPDH (Catalog No: AF7021) were purchased from Affinity Biosciences, Ltd. (Affinity, Changzhou, Jiangsu, China). The NF-κB pathway sampler kit (Catalog No: #9936) and anti-rabbit IgG, HRP-linked antibody (Catalog No: #7074) were bought from Cell Signaling Technology (Danvers, MA, United States).

### The Molecular Docking Analysis of Rhoifolin

Molecular docking is available for modeling interactions between small molecules and proteins. The compounds (MOL000010) were characterized based on the TCMSP database. Protein Data Bank was used to retrieve the three-dimensional structures of the target proteins, and the corresponding optimal structures of ligands were searched. In the docking process, each protein targeted the natural ligand (positive substrate) as the center of the active site. The specificity of each ligand was downloaded from PubChem. The relationship between the ingredient and the putative target was determined based on the TCMSP database. Network analysis was conducted to estimate the times of interaction among two nodes with a degree. The significant candidate targets were identified as ROF effect targets. Molecular docking was completed by glide module in Schrödinger Maestro software and carried out by SP method. The Protein Preparation Wizard module was used for protein processing.

### Animal Experiments

**FIGURE 1:**
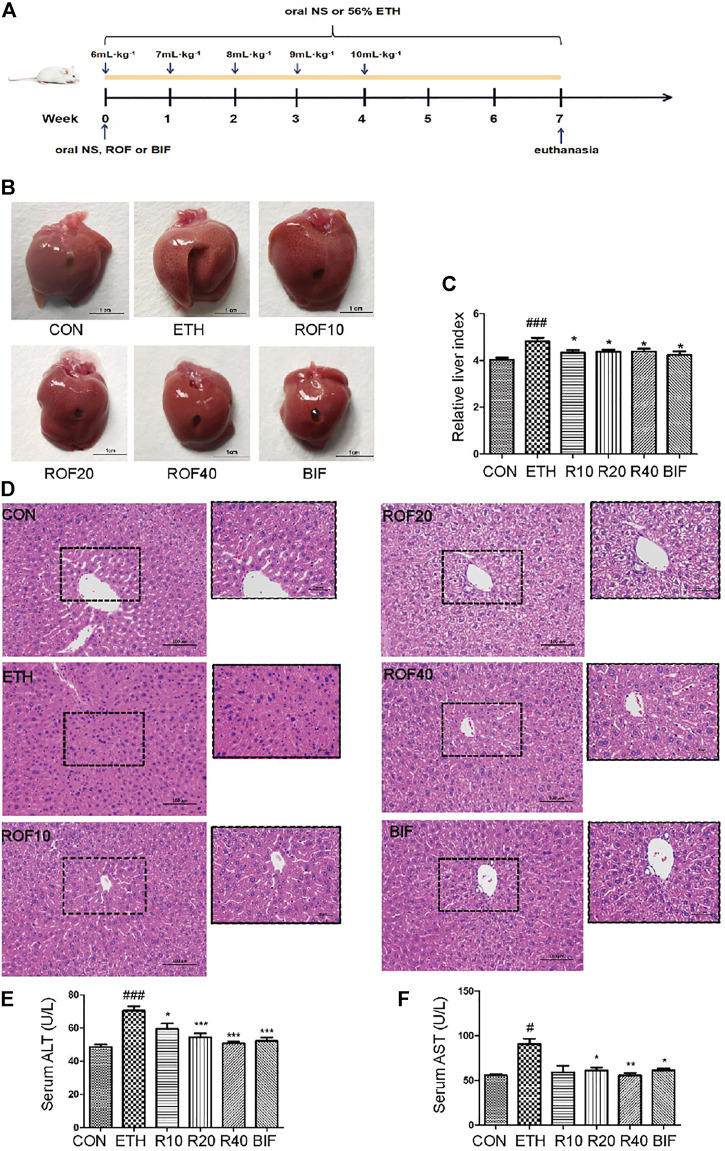
Effects of ROF on histopathological changes and hepatic index in ALD mice. The time flowchart is shown in **(A)**. Macroscopic appearance of liver tissues **(B)** and hepatic index **(C)**. **(D)** Histopathology analysis of liver damage (200× and 400×). The serum concentrations of ALT **(E)** and AST **(F)**. Mean ± SEM (*n* = 6–9). *p < 0.05, **p < 0.01 and ***p < 0.001 vs. the ETH group. ##*p* < 0.01 and ###*p* < 0.001 vs. the CON group.

BALB/c mice (male, 18–20 g) were obtained from the animal center of Guangzhou University of Chinese Medicine [laboratory animal production and use license numbers: SCXK (Yue) 2018–0,034 and SYXK (Yue) 2018–0,085, respectively]. This experiment was approved by the Institutional Review Board of Guangzhou University of Chinese Medicine (Number: 44005800008970). The mice were randomly divided into six groups: three ROF treatment groups (10, 20, and 40 mg kg^−1^), ethanol (ETH) group, control (CON) group, and bifendatatum (BIF) group (150 mg kg^−1^). To establish the mouse ALD model, all mice were fed with gradient ETH (56% ETH), except those in the CON group (Lu et al., 2015; Wang et al., 2017; Liu et al., 2019). The initial dose was 6 ml kg^−1^ for the first week and then elevated by 1 ml every week (up to 10 ml kg^−1^) for 7 weeks. At the same time, ROF was orally administrated for 7 weeks to determine its hepatoprotective effects (Figure 1A). After treatment, the mice were euthanized for blood sample collection. After removing the liver and weighing, a section of the liver was fixed, and the remaining part was kept at −80°C until analysis.

### Liver Function and Inflammatory Factors Analysis

After centrifugation (3,500 r min^−1^, 10 min, 4°C), the blood specimens were employed for biochemical analysis. The levels of AST and ALT were measured using the kits according to the kit’s protocol. The serum levels of TNF-α, IL-6, and IL-1β were determined using the ELISA kits in accordance with the kit’s protocol.

### Histological and Immunohistochemical Analyses

After fixing in 4% buffered paraformaldehyde, the hepatic tissues were cut, hydrated in a series of graded alcohol, cleared in xylene, embedded with paraffin, and sectioned at 4 μm thickness. After hematoxylin–eosin staining, the tissue sections were subjected to pathological evaluation using an optical microscope (Olympus Corporation, Japan). Finally, the degrees of inflammation and oxidative damage were assessed by p65 and CYP2E1 immunohistochemical staining.

### Malondialdehyde, Glutathione, and Superoxide Dismutase Levels

To prepare liver homogenate (10%), liver tissues were homogenized in PBS, followed by centrifugation (3,000 rpm, 10 min, 4°C). After collecting the supernatants, the activities of MDA, GSH, and SOD in the liver homogenate were evaluated using the kits according to the kit’s instructions. The total protein content in the liver was determined using the BCA assay kit. Normalization to total liver protein was then performed.

### Cell Culture

LO2 cell line was obtained from the Biological Sample Collection Center (BSCC, Wuhan, China). The cells were cultured in a DMEM (Gibco, Gland Island, United States) containing 10% fetal bovine serum (Gibco, Gland Island, United States) at 37°C and 5% CO_2_. In the ETH group, 200 mmol L^−1^ ETH was added. In the treatment groups, 200 µl of ETH (200 mmol L^−1^) and ROF (25, 50, and 100 mol L^−1^) were added. An equivalent amount of the serum-free medium was added to the blank cell wells.

### Cell Viability Assay

LO2 cells (5 ml^−1^×l0^3^ cells ml^−1^) were grown in a 96-well plate for 24 h. MTT assay was employed to evaluate the viability of cells. Briefly, MTT solution (5 mg mL^−1^) was added and incubated for 4 h at 37°C. After rinsing with PBS, 150 µl DMSO was used to dissolve the formed formazan. Finally, the optical density was recorded using a microplate reader (Thermo Fisher Scientific, United States) at 490 nm.

### Cellular Lactate Dehydrogenase, Alanine Aminotransferase, Aspartate Aminotransferase Glutathione, Malondialdehyde, and Superoxide Dismutase Assays

The LDH cytotoxicity assay kit was used to determine LDH release. First, LO2 cells were grown in 6-well plates and incubated with DMEM containing ROF or ETH for 24 h. The content of LDH in the medium was determined according to the kit’s protocol. ALT and AST levels in the supernatant were determined by commercial kits. At the same time, GSH, MDA, and SOD activities in the supernatant were measured using the commercial kits.

### Apoptosis Assay

Cell suspension (2 ml/well) was plated in a 6-well plate at 37°C for 12 h. After treatments, the supernatants and cells were harvested separately and resuspended in 1× binding buffer. Then, Annexin V-FITC (5 μl) and PI (10 μl) were added and incubated for 5 min, followed by flow cytometric analysis (NovoCyte Quanteon, United States).

### ROS Measurement

ROS was assessed in LO2 cells by the Reactive Oxygen Species Assay Kit of DCFH-DA. After incubation for 24 h, 10 μM DCFH-DA was added to cell medium without serum and incubated for 30 min at 37°C. After rinsing two times with PBS, ROS levels were determined using a fluorescence microplate reader (Biotek, Vermont, United States) with the emission and excitation wavelengths set at 525 and 500 nm, respectively.

### Real-Time PCR

Total RNA was extracted from LO2 cells using the TRIzol reagent (Cwbio, Beijing, China), followed by quantification and dilution into 1 mg mL^−1^. cDNA synthesis was conducted with HiScript RT SuperMix (Vazyme, Nanjing, China) by following the kit’s instructions. Real-time PCR was conducted with SYBR qPCR Master Mix (Vazyme, Nanjing, China). Melting curve analysis was performed to assess the purity and identity of PCR products. The primer pairs (Table 1) were synthesized by Sangon Biotech (Shanghai, China).

**TABLE 1:** Primer sequences of each gene.

Gene	Forward (5′-3′)	Reverse (5′-3′)
GAPDH	ACT CCT CCA CCT TTG ACG CT	GGT CTC TCT CTT CCT CTT GTG C
TNF-α	TGG GAT CAT TGC CCT GTG AG	GGT GTC TGA AGG GGG TA
IL-6	TAG TGA GGA ACA AGC CAG AGC	GTT GGG TCA GGG GTG GTT ATT
IL-1β	AGT TGA CGG ACC CCA AA	TCT TGT TGA TGT GCT G
Bax	CCC CGA GAG GTC TTT TTC CG	CCG GAG GAA GTC CAA TGT CC
Bcl-2	GGT GAA CTG GGG GAG GAT TG	GTG CCG GTT CAG GTA CTC AG

### Western Blotting

Protein content was detected using the BCA assay kit. After separation through 10% SDS-PAGE, the protein specimens were transferred onto PVDF membranes. The membranes were exposed to primary antibodies antiCYP2E1, IκBα, p-IκBα, p65, p-p65, NLRP3, TLR4, caspase 3, cleaved caspase 3, tubulin-β, and GAPDH diluted in 5% BSA-containing TBST at 4°C overnight. After rinsing with TBST, the membranes were incubated with rabbit antibodies for 1 h at room temperature. Lastly, the membranes were rinsed again with TBST and visualized with ECL solution.

### Statistical Analysis

The experimental results were analyzed by IBM SPSS Statistics 23. All values were expressed as means ± SEM. The data in this study followed a normal distribution, and the one-way analysis of variance (ANOVA) was employed. Tukey’s test and Dunnett’s T3 test were applied when the variances were uniform and not uniform, respectively. A T-test was used for comparison between the two groups. The results of the statistical analysis were graphed in GraphPad Prism 5.0. The level of statistical significance was set at *p* < 0.05.

## Results

### Docking Results of Rhoifolin and Target Protein of Alcoholic Liver Injury

Under normal conditions, ETH is released in the stomach and the small intestine and enters the liver via the bloodstream. The liver is the primary site of ETH metabolism, and < 10% of ETH is excreted unchanged in sweat and through the lung and kidney (Louvet and Mathurin, 2015). The activity and expression of CYP2E1 are induced by alcohol intake, which catalyzes the oxidation from ETH to acetaldehyde. Its metabolites can produce hepatocellular toxicity and increase the risk of ALD (Nagappan et al., 2018). Primarily, this study evaluated the binding of ROF to hepatic CYP2E1. Excessive oxidative stress and inflammation caused the destruction of cellular ingredients and cell apoptosis, which played a vital role in the occurrence of ALD. Bcl-2 and caspase 3 can reflect apoptosis to a certain extent (Hung et al., 2021; Xiao et al., 2021). We simulated the binding of ROF to Bcl-2 and caspase 3. TLR4 is closely associated with inflammation in alcohol-induced liver injury by activating the NF-κB pathway and regulating the expression of inflammatory factors (Hu et al., 2020). We simulated and evaluated the binding of ROF to TLR4 protein, an upstream of the NF-κB pathway. The structures of Bcl-2 (PDB ID:6OQC), CASP3 (PDB ID:1NMS), p65 (PDB ID:2O61), TLR4 (PDB ID:2Z62), and CYP (PDB ID:3E6I) target proteins were retrieved from the RCSB database. The binding energy and binding modes of ROF (MOL000010) with TLR4, p65, Bcl-2, caspase 3, and CYP2E1 are summarized in Table 2 and Supplementary Figure S2. The results showed that ROF could bind to five target proteins, thus forming stable complexes with these proteins.

**TABLE 2:** Target protein docking results for compounds.

Target	Compound	Compound structure	Binding energy (kcal/mol)	Combination type
TLR4	MOL000010	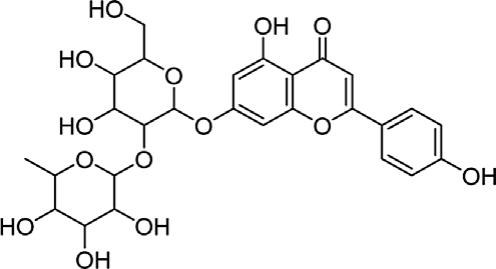	−6.64	HB, HI
p65			−6.92	HB, HI
Bcl-2			−8.08	HB, HI
Caspase-3			−7.71	HB, HI
CYP2E1			−8.28	HB, HI

HB, hydrogen bond; HI, hydrophobic interaction.

### Rhoifolin Relieves the Alcohol Liver Injury in Mice

To assess the protective effect of ROF on ETH-induced liver injury in mice, we first established the experimental chronic alcoholic liver injury models and evaluated the morphological changes in liver tissues and liver index from six different groups (Figures 1B, C). The macroscopic view of liver surfaces indicated that the liver of the model group was obviously swollen, and ROF could alleviate liver damage to some extent. Bifendatatum and different concentrations of ROF also markedly decreased the liver/body weight ratio of ALD mice. The histological changes showed that radiating hepatic cords containing normal lobular architecture was observed in the liver tissues of the CON group. After 7 weeks of intragastric ETH administration, the liver tissues of ALD mice showed that the normal architecture of liver tissues has disappeared, along with obvious disordered hepatic cords, narrowed hepatic sinusoids, liver cell swelling, and inflammatory cell infiltration. Interestingly, bifendatatum and different concentrations of ROF could remarkably attenuate the degree of histopathological alterations in the liver. The structures of hepatic cords in all treatment groups were clear, and there was no obvious lymphocyte infiltration in the liver (Figure 1D). In addition, we observed that ROF could promote the regrowth of functional liver cells. The serum levels of AST and ALT in ALD mice were dramatically higher than in control mice. Treatment with ROF markedly reversed the elevation of these indicators (Figures 1E, F). The biochemical data are in good agreement with the histological findings.

### Rhoifolin Improves Oxidative Stress in Liver and Inhibits Inflammatory Factors’ Expression in Serum

**FIGURE 2:**
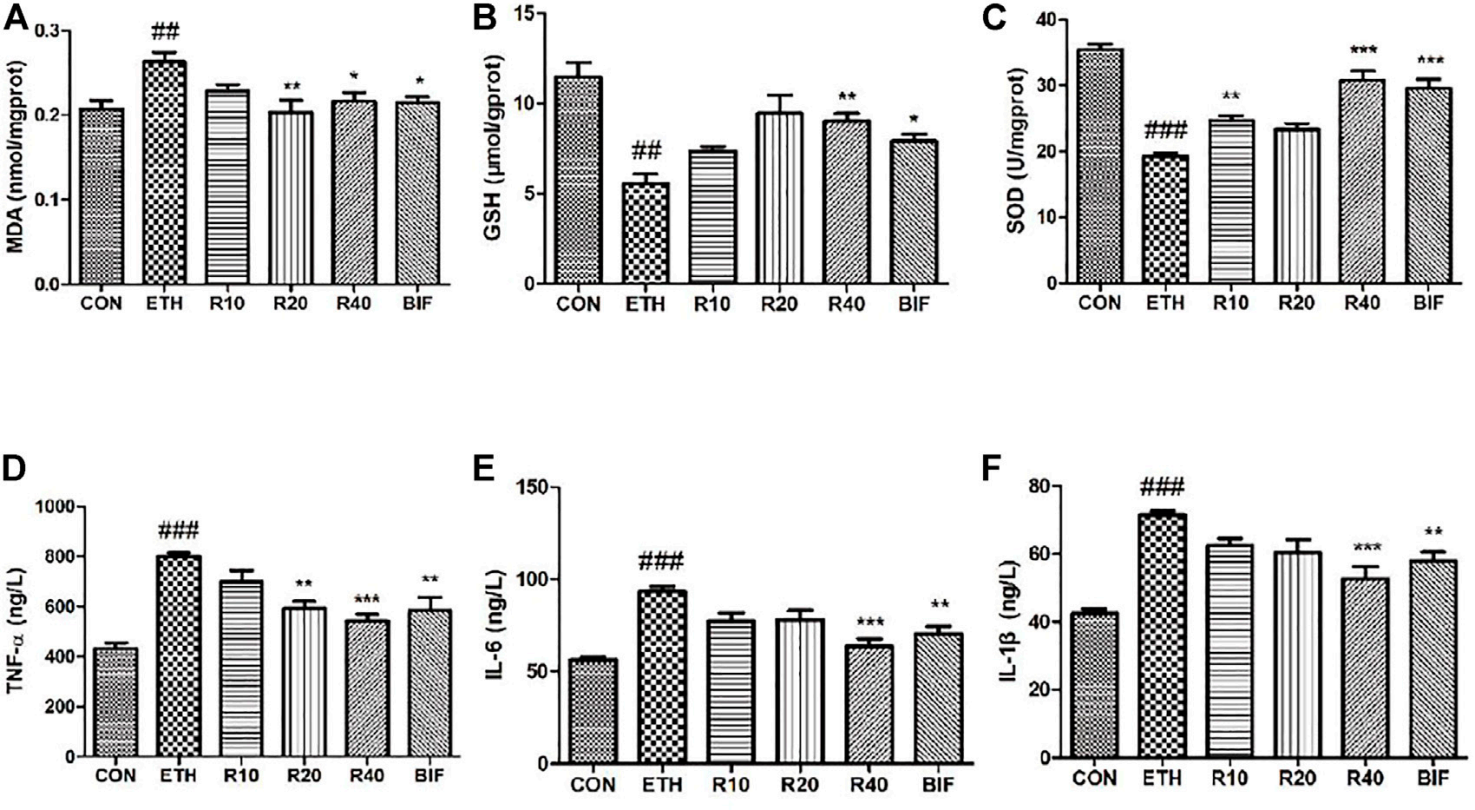
Effects of ROF on the level of oxidative stress indicators and the secretion of inflammatory cytokines in ALD mice. The levels of MDA **(A)**, GSH **(B)**, SOD **(C)**, TNF-α **(D)**, IL-6 **(E)**, and IL-1β **(F)**. Mean ± SEM (*n* = 6). *p < 0.05, **p < 0.01, and ***p < 0.001 vs. the ETH group. ###*p* < 0.001 vs. the CON group.

The levels of MDA, GSH, and SOD in the supernatant of liver homogenate were determined according to the colorimetric method, and the levels of TNF-α, IL-6, and IL-1β within serum were measured using ELISA kits. Compared to the control mice, the MDA level was markedly elevated while GSH and SOD levels were dramatically reduced in the liver tissues of the ETH-treated mice (Figures 2A–C). Different concentrations of ROF and bifendatatum decreased the MDA levels and reversed the ETH-induced reductions of GSH and SOD activities. The serum levels of IL-1β, IL-6, and TNF-α were markedly elevated in the ETH group compared to the CON group but dramatically reduced after treatment with ROF and bifendatatum (Figures 2D–F). Taken together, our findings indicate that ROF can mediate the levels of inflammatory cytokines and oxidative stress-related factors.

### Rhoifolin Downregulates CYP2E1 Protein and TLR4/NF-κB Signaling Pathways in Mice

**FIGURE 3:**
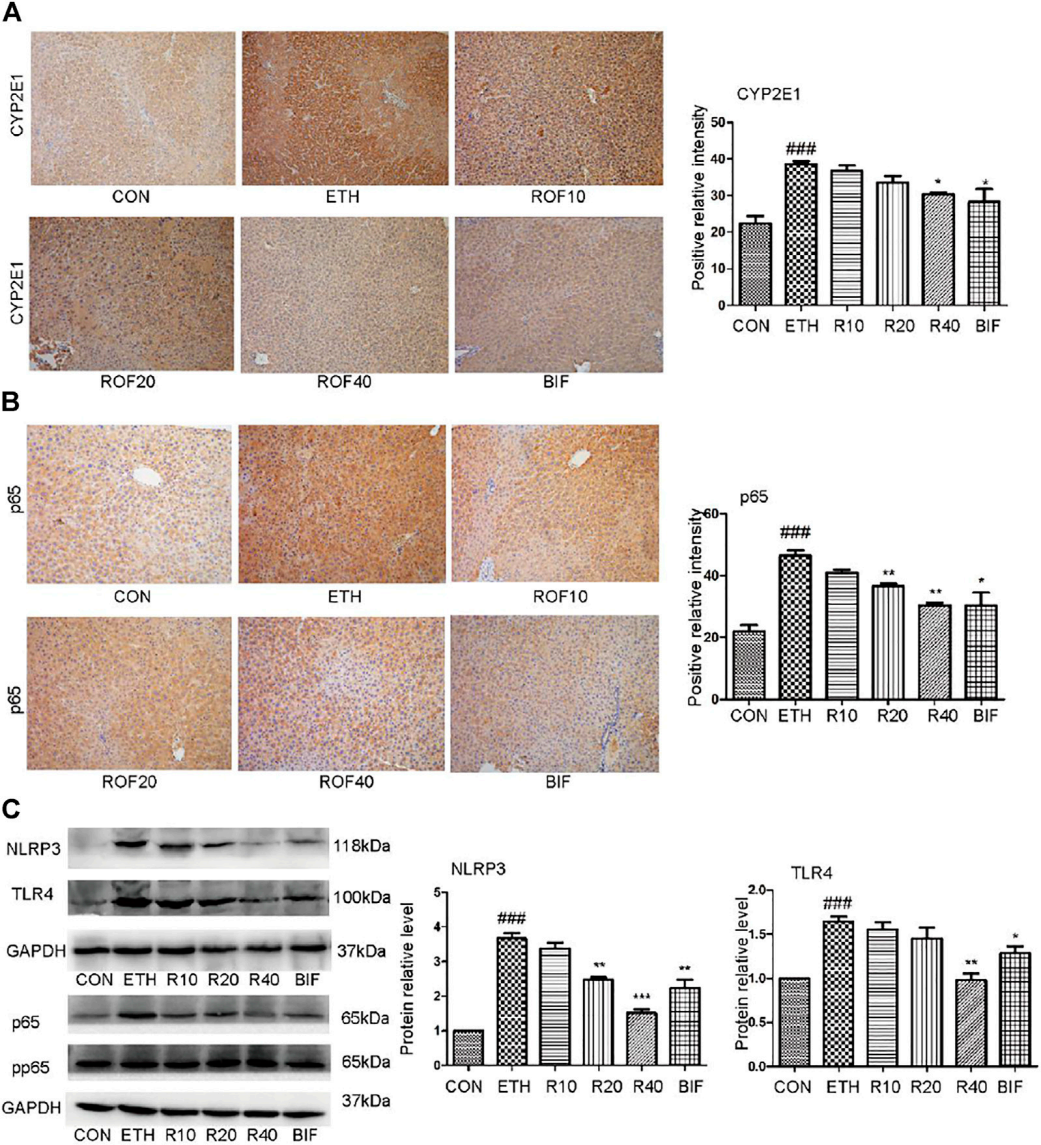
Effect of ROF on CYP2E1 protein and TLR4/NF-κB axis in mice. The expression of CYP2E1 **(A)** and p65 **(B)** in the liver tissues detected by immunohistochemical staining (×200 magnification ). The protein levels of NLRP3, TLR4, and pp65 were detected by Western blotting **(C)**. Mean ± SEM (*n* = 3). *p < 0.05, **p < 0.01 and ***p < 0.001 vs. the ETH group. ##*p* < 0.01 vs. the CON group.

It has been reported that the upregulated expression of CYP2E1 and activation of the NF-κB pathway are involved in the pathogenesis of ETH-induced liver injury (Leung and Nieto, 2013; Harjumaki et al., 2021; Zhang et al., 2021). Immunohistochemistry and Western blot were employed to detect CYP2E1 protein expression and TLR4/NF-κB signaling pathway in the experimental animal liver. Immunohistochemical staining revealed that ETH markedly induced hepatic CYP enzyme and inflammatory cytokines in the liver. The ETH group significantly upregulated the expression of CYP2E1 and NF-κB compared to the CON group (Figures 3A, B). Treatment with ROF and bifendatatum noticeably downregulated the expression of CYP2E1 and NF-κB. To further examine the role of the NF-κB pathway in ETH-induced inflammation, the protein levels of NLRP3, TLR4, and pp65 were determined by Western blotting (Figure 3C). These proteins were highly expressed in the ETH group compared to the CON group. Treatment with ROF and bifendatatum noticeably reduced the expression levels of these proteins. These findings imply that ROF could protect against hepatic damage in ALD mice by inhibiting CYP2E1 and NF-κB expression.

### Rhoifolin Inhibits Apoptosis and Increases the Viability of ETH-Treated LO2 Cells

**FIGURE 4:**
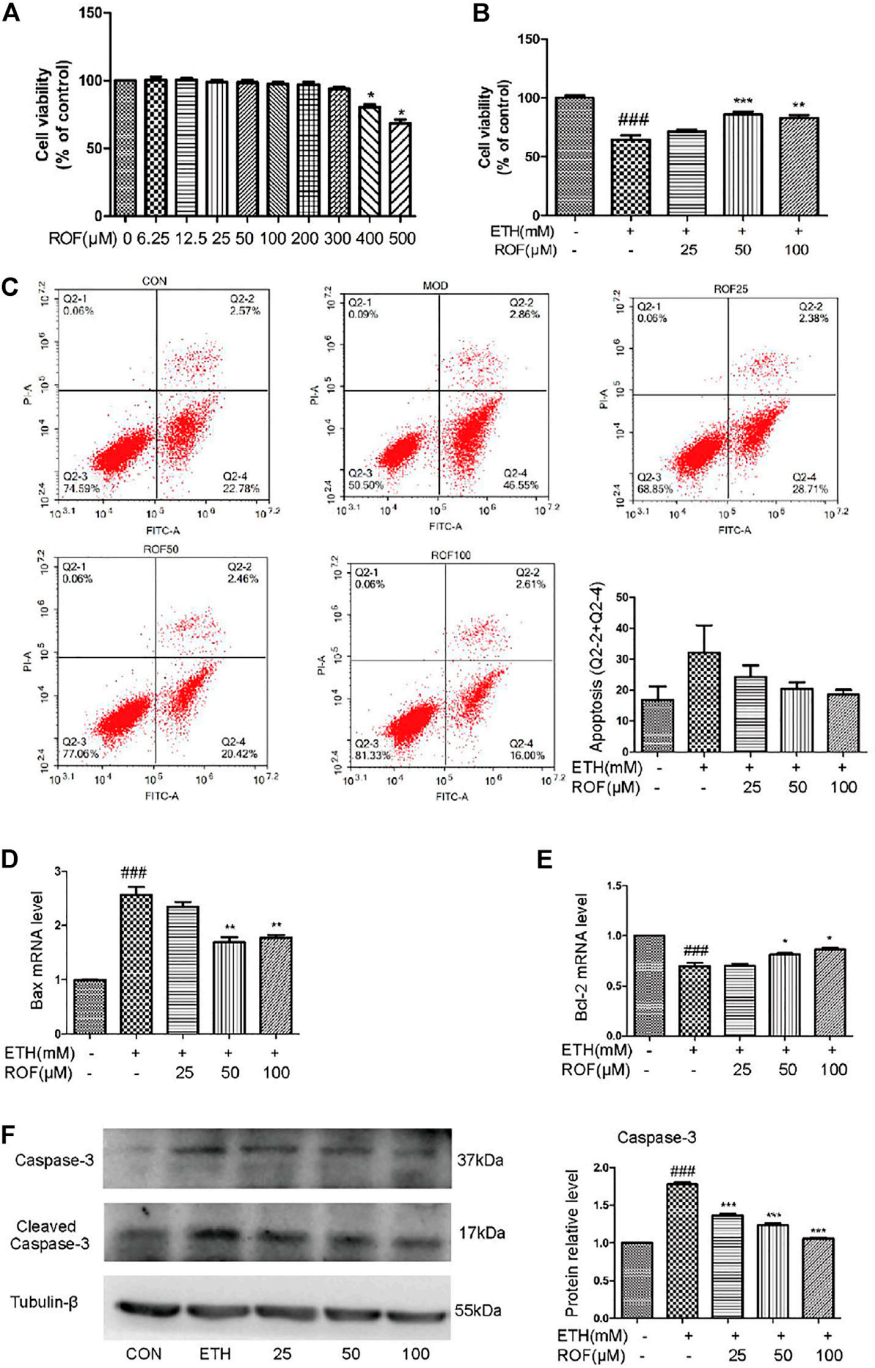
Effects of ROF on cell apoptosis and viability: **(A**,**B)** Cell viability of ETH (200 mm) and ETH + ROF groups after treatment for 24 h. The apoptotic rate **(C)**, Bax expression **(D)**, and Bcl-2 expression **(E)** in LO2 cells. The protein levels of caspase 3 and cleaved caspase 3 **(F)**. Mean ± SEM (*n* = 3–6). *p < 0.05, **p < 0.01 and ***p < 0.001 vs. the ETH group. ###*p* < 0.001 vs. the CON group.

The aforementioned results have confirmed that ROF relieved the ETH-induced hepatic injury in mice. To further evaluate the protective mechanism of ROF, human normal hepatocyte LO2 cells were cultured *in vitro* to observe whether ROF can improve hepatocyte apoptosis. LO2 cells were exposed to ETH (0, 50, 100, 200, 300, 400, 500, or 600 mm) to mimic the pathological features of alcohol-induced liver damage (Shao et al., 2019; Liu F et al., 2020). When the ETH concentration was 200 mm, the cell activity decreased to about 60% (Supplementary Figure S4). Treatment with ROF increased the viability of LO2 cells exposed to ETH (Figures 4A, B). Annexin V-FITC/PI and flow cytometric analysis were conducted to examine apoptosis induced by ETH (Figure 4C), while real-time PCR was employed to assess the RNA expression of apoptosis-related genes (Figures 4D, E). Following ETH exposure, the expression levels of Bax and Bcl-2 were upregulated and downregulated, respectively. ROF downregulated Bax expression, upregulated Bcl-2 expression, and decreased the ratio of Bax/Bcl-2. Furthermore, the protein levels of caspase 3 and cleaved caspase 3 are demonstrated in Figure 4F. The expression levels of these proteins were elevated in ETH-treated cells compared to control cells. Treatment with ROF markedly downregulated the expression levels of these proteins in ETH-treated LO2 cells.

### Rhoifolin Regulates the Expression of ALD-Related Factors in LO2 Cells.

**FIGURE 5:**
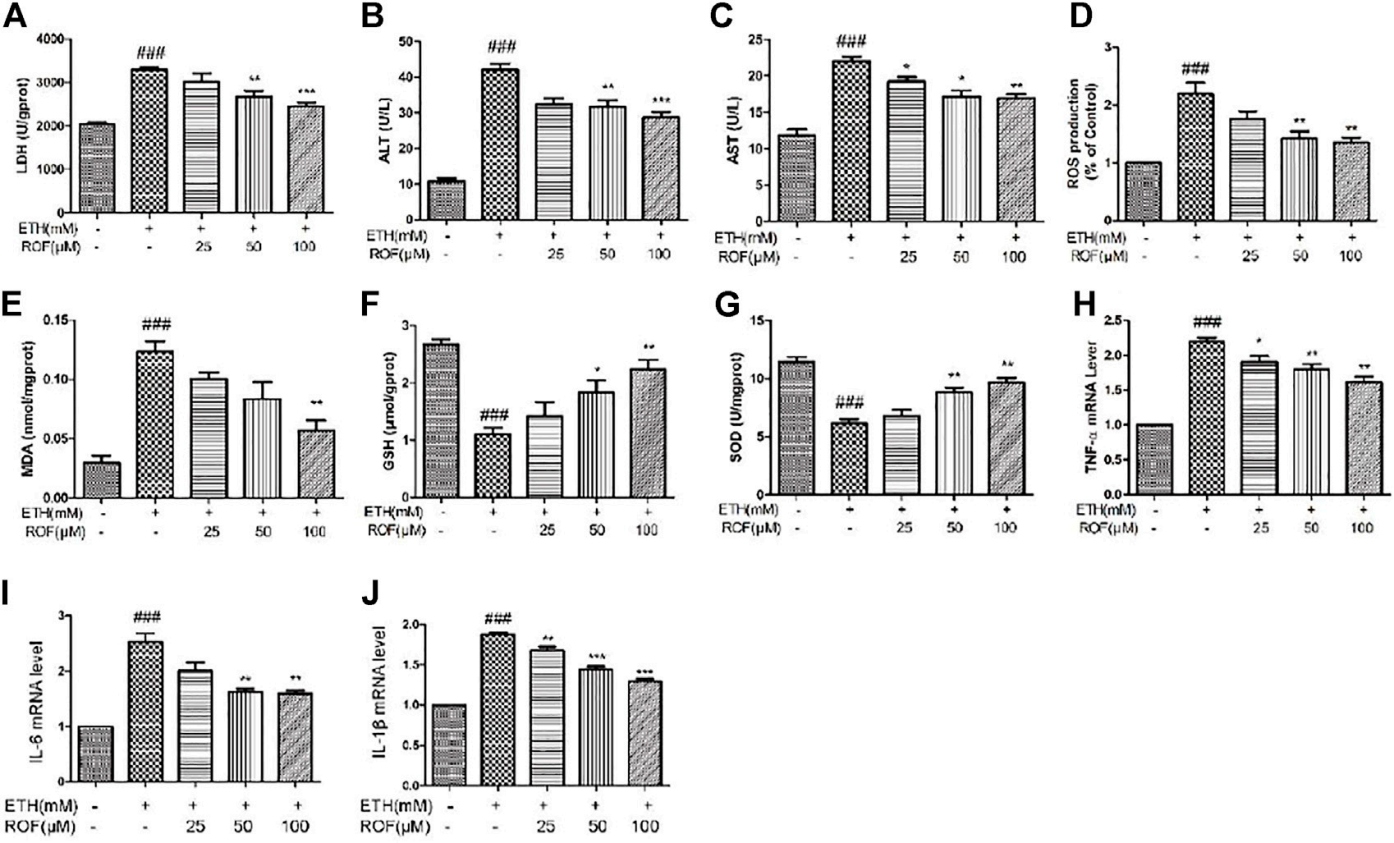
Effect of ROF on the production of LDH **(A)**, ALT **(B)**, AST **(C)**, ROS **(D)**, MDA **(E)**, GSH **(F)**, and SOD **(G)** in ETH-treated hepatocytes. The cells were exposed to ETH (200 mm) and ETH + ROF (25, 50, 100 μM) for 24 h. The expression levels of TNF-α **(H)**, IL-6 **(I)**, and IL-1β **(J)** in LO2 cells. Mean ± SEM (*n* = 3–4). *p < 0.05, **p < 0.01, and ***p < 0.001 vs. the ETH group. ##*p* < 0.01 and ###*p* < 0.001 vs. the CON group.

*In vitro*, we also detected the oxidative stress levels and expression of inflammatory cytokine in the LO2 cell supernatant. LDH leakage has been successfully used to measure cytotoxicity. ETH exposure induced LDH release into the culture medium, and ROF markedly decreased the content of LDH in the culture medium (Figure 5A). Exposure to ETH released a massive release of aminotransferases from LO2 cells. The concentrations of AST and ALT were higher in ETH-treated cells compared to control cells. ROF at 25, 50, and 100 µM could prevent the increase in AST and ALT levels in the culture supernatants (Figures 5B,C). The ROS level increased in the ETH groups compared with the CON group. The levels of ROS obviously reduced in the ROF group compared with the ETH group (Figure 5D).

The MDA level was remarkably elevated, and GSH and SOD activities were markedly reduced in the supernatant of the ETH group compared to that of the control group (Figures 5E–G). ROF decreased MDA content and reversed ETH-induced decreases in GSH and SOD activities. To assess whether ROF can exhibit anti-inflammatory effects, the expression levels of IL-1β, IL-6, and TNF-α were detected (Figures 5H–J). The expression levels of IL-1β, IL-6, and TNF-α were dramatically elevated in ETH-treated LO2 cells, and ROF could decrease the production of these inflammatory cytokines.

### Rhoifolin Downregulates CYP2E1 Protein Expression and TLR4/NF-κB Signaling Pathway in LO2 Cells

**FIGURE 6:**
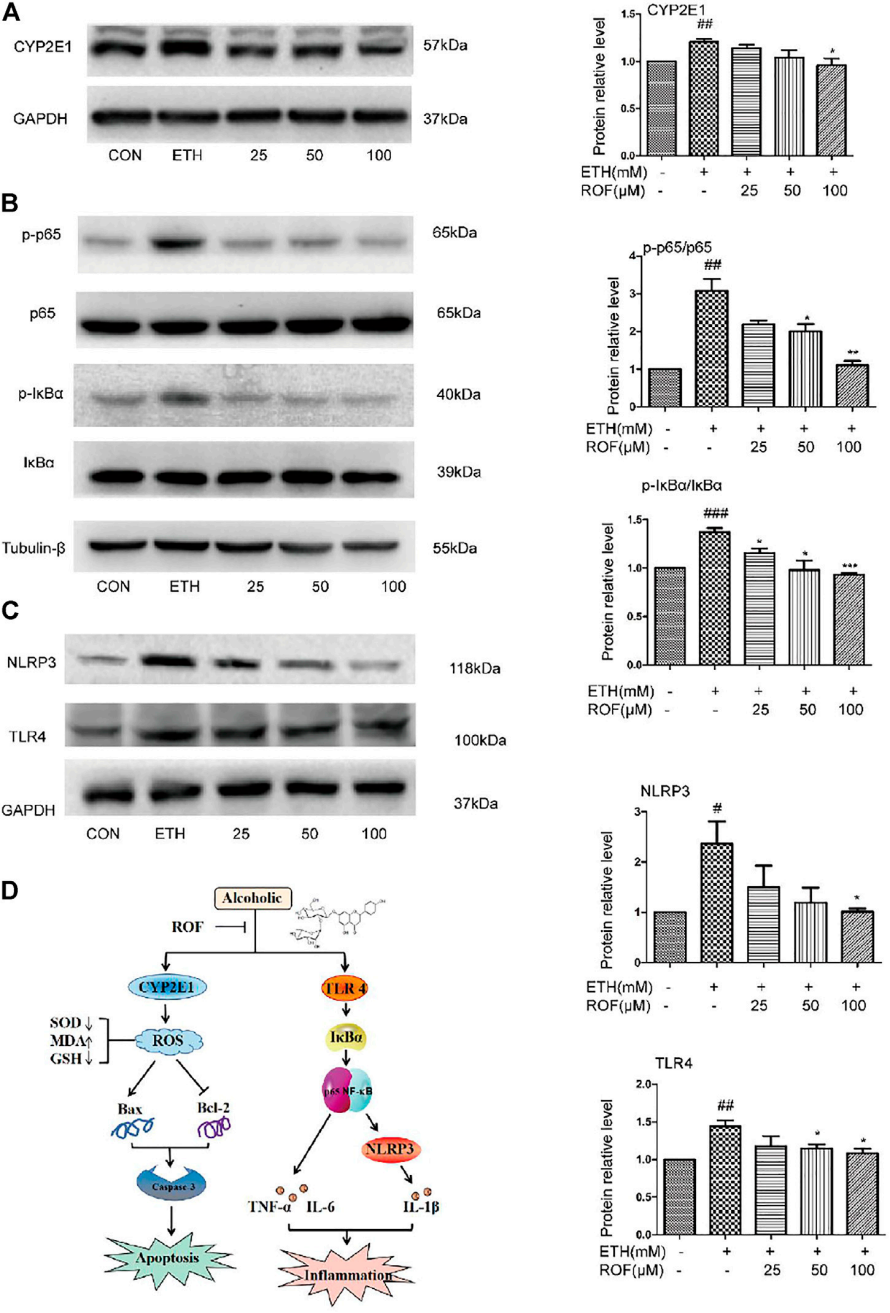
Effects of ROF on CYP2E1 protein expression and TLR4/NF-κB signaling pathway in LO2 cells. The cells were assigned to CON, ETH, and ROF (25, 50, 100 μmol L^−1^) groups. The expression levels of CYP2E1 **(A)**, and TLR4, NLRP3, IκBα, p65, and their phosphorylated proteins were evaluated by Western blotting **(B**, **C)**. The mechanism by which ROF alleviates ALD is shown in **(D)**. Mean ± SEM (*n* = 3). *p < 0.05, **p < 0.01, and ***p < 0.001 vs. the ETH group. #*p* < 0.05, ##*p* < 0.01, and ###*p* < 0.001 vs. the CON group.

To clarify the mechanisms underlying the hepatoprotective effects of ROF on ETH-induced oxidative stress, the protein expression of CYP2E1 was evaluated (Figure 6A). After ETH induction, the expression of CYP2E1 protein in LO2 cells was significantly increased, while ROF downregulated its expression. These results indicate that CYP2E downregulation is involved in the hepatoprotective effects of ROF against ETH-induced oxidative stress.

To clarify the molecular mechanism of ROF in ETH-induced LO2 cells inflammatory damage. We detected the expression and phosphorylation of NF-κB pathway-related proteins in LO2 cells (Figures 6B, C). The protein levels of TLR4, NLRP3, p65, and IκBα were much higher in the supernatant of ETH-treated cells than in that of control cells. Compared with the ETH group, ROF (50 and 100 µmol L^−1^) greatly downregulated the expression of these proteins in LO2 cells. These results indicate that ROF can alleviate hepatic inflammation via the regulation of the TLR4/NF-κB axis.

## Discussion

Heavy alcohol use is the main cause of ALD, which can progress to steatosis, hepatitis, fibrosis/cirrhosis, and even liver cancer (Xu et al., 2018). Although the mechanism remains inconclusive, inflammatory responses and oxidative stress have been implicated in the pathogenesis of ALD (Qu et al., 2019). Several drugs have been used to treat alcoholic hepatitis. Glucocorticoids (e.g., prednisolone) and hepatic protectants (e.g., bifendatatum) have been used for the treatment of alcoholic injury, and several experimental trials have been conducted to determine their efficiency (Farooq and Bataller, 2016; Deng et al., 2020), although they have side effects. Many other treatments for alcoholic liver injury, such as antioxidants, chelating agents, hepatotropic hormones, and TNF-α antagonists (Lee et al., 2017), have been considered controversial. In recent years, traditional Chinese medicine has shown good therapeutic effects for ALD. Herbal medicine monomers or formulas have gained increasing attention as potential agents for the treatment and prevention of ALD. Preparations of Chinese medicine such as Gegen powder (Taotao et al., 2017), Chinese herb extracts including total flavonoids from hovenia acerba (Jinglong et al., 2020), and monomers such as glycyrrhizic acid (Huo et al., 2018) have been shown to protect against experimental ALD. However, there are no reports pertaining to the application of TCM small molecule drugs for the clinical treatment of ALD. Modern pharmacological research has shown that flavonoids are the main active ingredients of *Citrus grandis* “Tomentosa,” with their anti-inflammation, antioxidant, analgesic, and antipyretic effects, which have a significant effect on ALD (Xiao et al., 2012a). ROF is one of the most important flavonoids in this plant. In comparison with other monomers’ active ingredients for the treatment of ALD, the extraction, and preparation of ROF arerelatively simple. However, there is no in-depth analysis on the hepatoprotective effects of ROF against ETH-induced liver damage. Thus, the present study was undertaken to explore the protective effects of ROF and its underlying mechanisms.

In the present study, the therapeutic mechanisms of ROF were investigated via molecular docking studies and experimental validation. The TCM database was applied to determine the potential targets of ROF and network analysis was used to identify the candidate targets for further exploration of the therapeutic mechanism in ALD (Fazi et al., 2015; Gong et al., 2019). TLR4, p65, Bcl-2, caspase 3, and CYP2E1 target proteins were docked by ROF (MOL000010). The five target proteins had good binding sites, with a binding energy of less than −6 kcal/mol. Thus, it is speculated that ROF can regulate the TLR4/NF-κB signaling pathway after binding to these protein targets. However, the findings of molecular docking need further biological validation.

In order to address the biological verification needs, the hepatoprotective effects of ROF on ETH-induced liver damage were assessed both *in vitro* and *in vivo*. Bifendatatum was treated as a positive control for assessing ROF *in vivo*, as it has previously been proven to be an anti-hepatitis drug via its mechanisms of reducing alanine transaminase secretion (Deng et al., 2020; Xu S. et al., 2021). Bifendatatum has been used as a positive control in many alcoholic liver injury animal studies (Wang et al., 2012; Bian et al., 2021). ROF at moderate or high doses could effectively protect against the mouse’s liver, which was comparable to bifendatatum in numerous studies. The results indicated that ROF attenuated ETH-induced liver damage via its antioxidant and anti-inflammatory effects, mainly through downregulating apoptosis-related genes and inflammatory cytokines as well as suppressing TLR4/NF-κB axis. This was consistent with the molecular docking results.

Following the investigations into the mechanisms, the causes of alcoholic liver injury were explored in detail. Previous studies have shown that CYP2E1 is involved in alcohol-induced oxidative stress injury (Chen et al., 2016; Liu J et al., 2020). When various pathogenic factors lead to oxidation and antioxidant imbalance, excessive ROS can be produced, resulting in oxidative stress injury (Cao et al., 2015; Lu et al., 2015; Ma et al., 2020). In our results, ROF improves oxidative stress by regulating the expression levels of GSH, SOD, and MDA. The inflammatory response also plays an important role in the development of ALD (Xu J. J. et al., 2021). This study shows that ROF significantly reduces the production of inflammatory cytokines. It is speculated that anti-inflammatory is also the main factor for ROF to play a therapeutic role.

Some scholars further studied the relationship between CYP2E1 protein and the NF-κB signaling pathway (Jia et al., 2015). NF-κB is an important transcription factor that regulates a wide range of genes, including the drug-metabolizing enzyme, CYP2E1 family (Qin et al., 2014). CYP2E1 overexpression promotes the sensitivity of Kupffer cells (Ye et al., 2013). At the same time, CYP2E1 inducer (pyrazole) can activate the NF-κB signaling pathway, promote oxidative stress and inflammation, and aggravate liver injury, while the effect of an enzyme inhibitor (clomethiazole) is just the opposite (Jiang et al., 2021). Our results show that ROF can downregulate abnormal CYP2E1 and inhibit the activity of the NF-κB signaling pathway, but whether ROF plays a therapeutic effect by affecting the interaction between CYP2E1 and NF-κB signaling pathway needs to be further studied.

Overall, ROF plays major roles in anti-inflammation, antioxidative, and antiapoptosis through the inhibition of CYP2E1 protein expression and regulation of TLR4/NF-κB signaling pathways, thus protecting against ETH-induced liver damage. In this article, the effects and mechanisms of ROF on ALD were discussed. Our next research will focus on the role of ROF in improving alcohol metabolism and liver fibrosis.

## Conclusion

In summary, ROF displayed protective effects on an ETH-induced ALD mouse model and ETH-treated LO2 cells. The results demonstrate that ROF can be applied as a promising herbal drug for ALD and provide an experimental basis for the rational use of *Citrus grandis* “Tomentosa.” ROF may serve as a candidate target for ALD treatment, and therefore, more clinical trials should be carried out to validate the clinical effectiveness and safety of this herbal medicine in ALD patients.

## Data Availability

The original contributions presented in the study are included in the article/Supplementary Material, further inquiries can be directed to the corresponding author.
