# Supplementary material for: Rhoifolin Alleviates Alcoholic Liver Disease In Vivo and In Vitro via Inhibition of the TLR4/NF-κB Signaling Pathway
Source: Front Pharmacol. 2022 May 24;13:878898. doi: 10.3389/fphar.2022.878898 (PMC9171502; doi:10.3389/fphar.2022.878898)
Supplement: Supplementary file 1 [file DataSheet2.pdf]

Supplementary Figure

Original Gels

In Figure.3

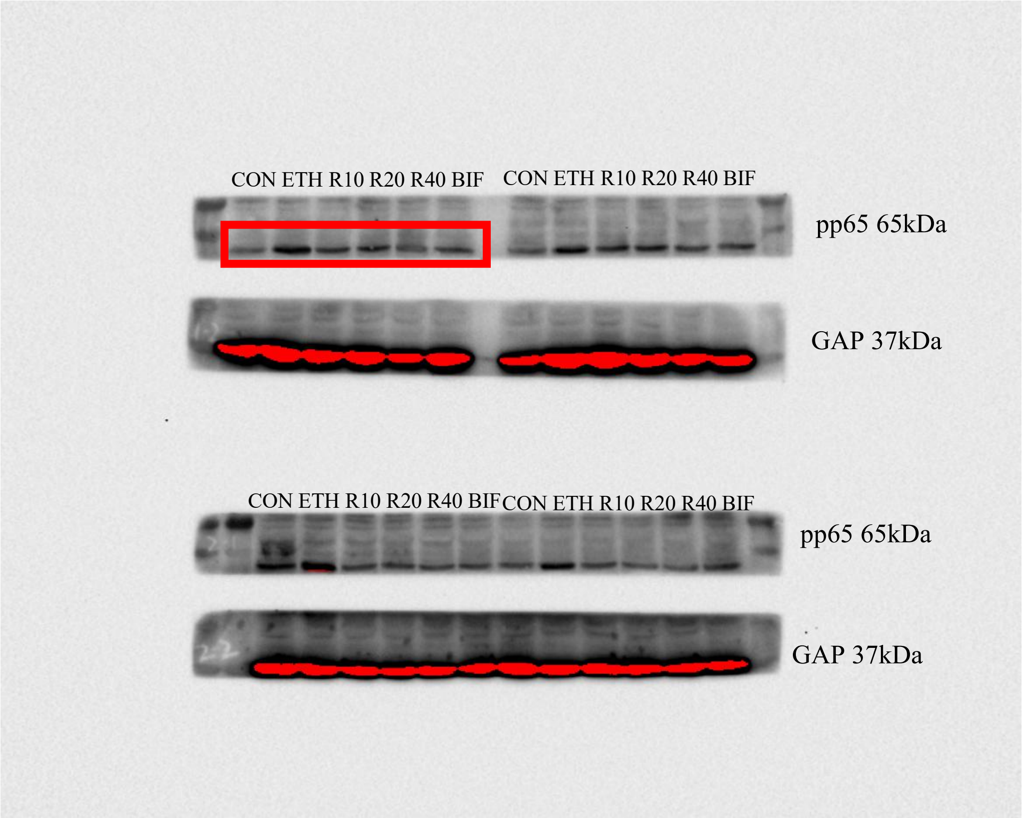

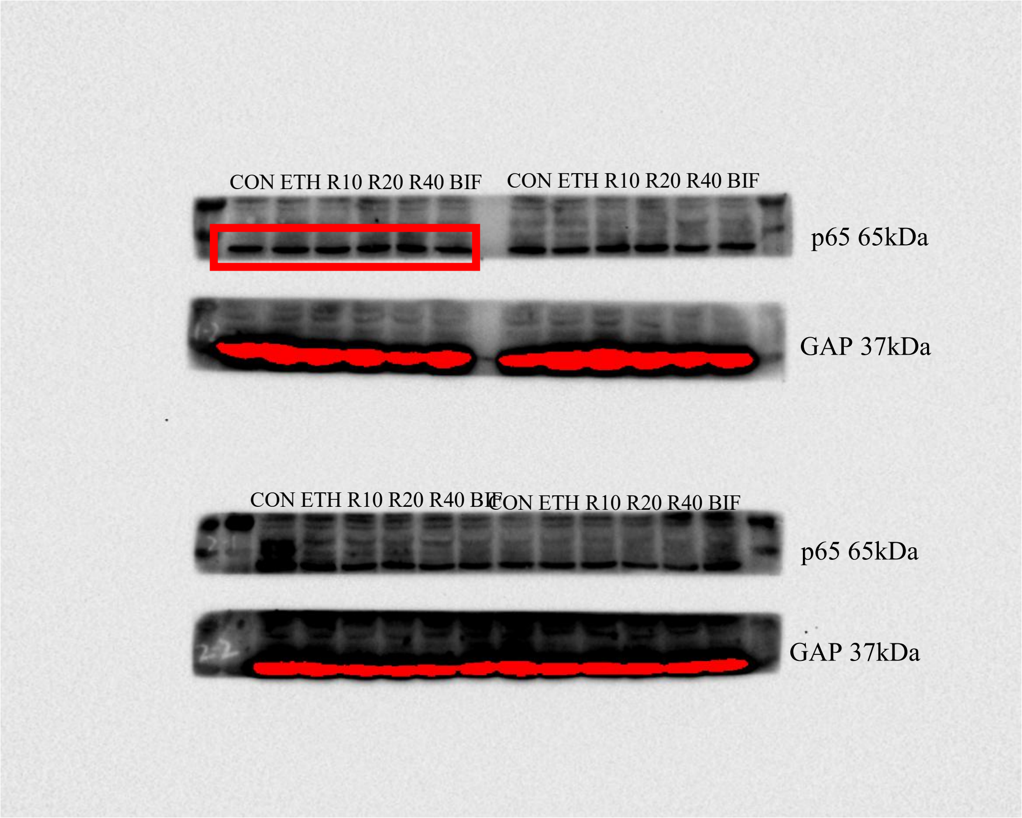
CON ETH R10 R20 R40 BIF CON ETH R10 R20 R40 BIF
p65 65kDa
GAP 37kDa
CON ETH R10 R20 R40 BIF CON ETH R10 R20 R40 BIF
p65 65kDa
GAP 37kDa

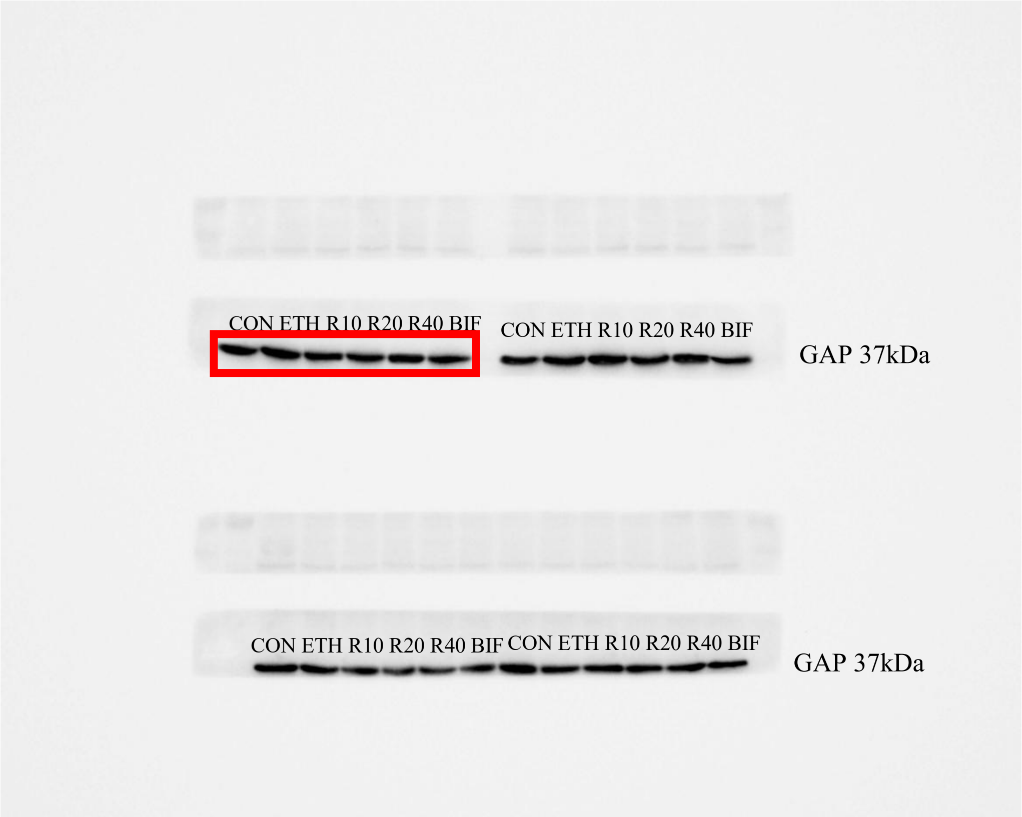
CON ETH R10 R20 R40 BIF CON ETH R10 R20 R40 BIF
GAP 37kDa
CON ETH R10 R20 R40 BIF CON ETH R10 R20 R40 BIF
GAP 37kDa

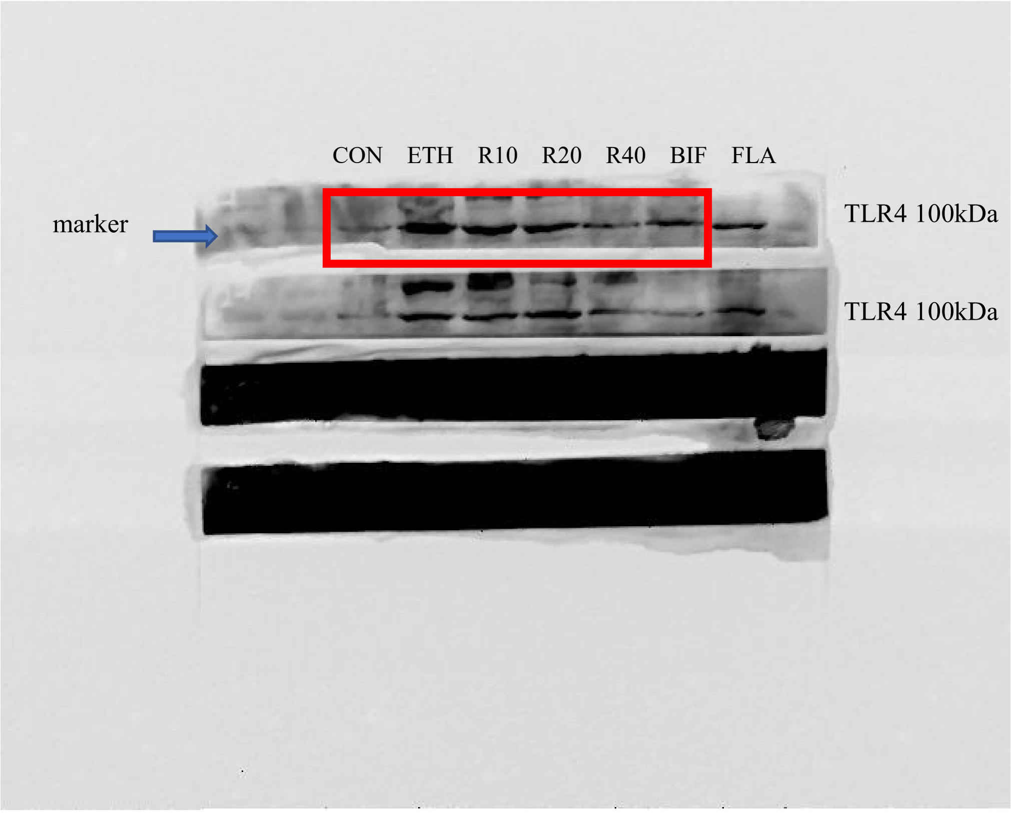
CON ETH R10 R20 R40 BIF FLA
marker
TLR4 100kDa
TLR4 100kDa

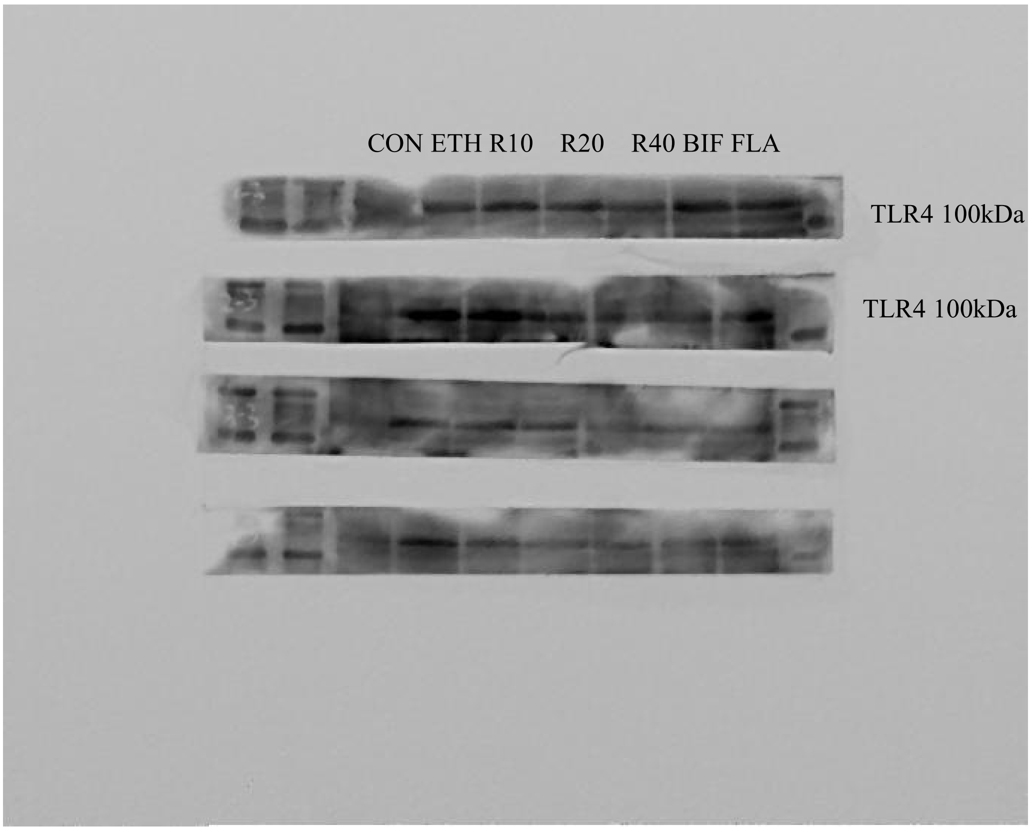
CON ETH R10 R20 R40 BIF FLA
TLR4 100kDa
TLR4 100kDa

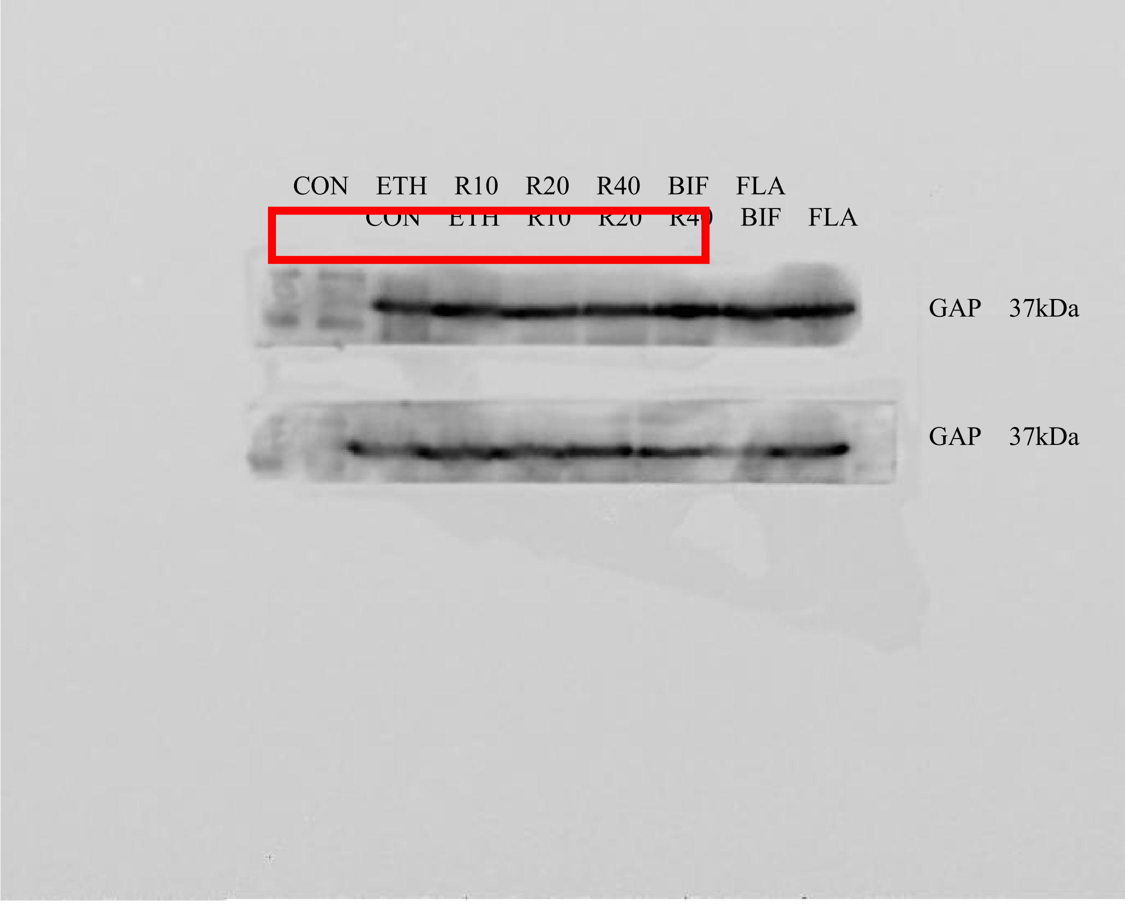
CON ETH R10 R20 R40 BIF FLA
CON ETH R10 R20
BIF FLA
GAP 37kDa
GAP 37kDa

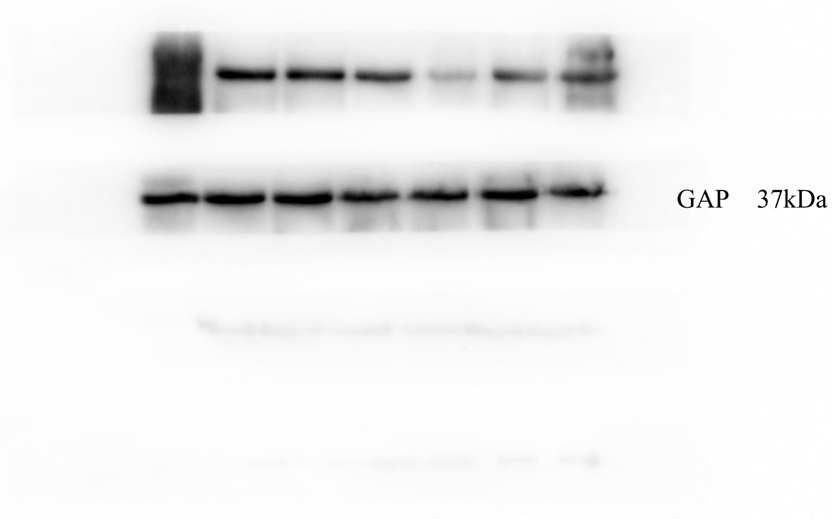
GAP 37kDa

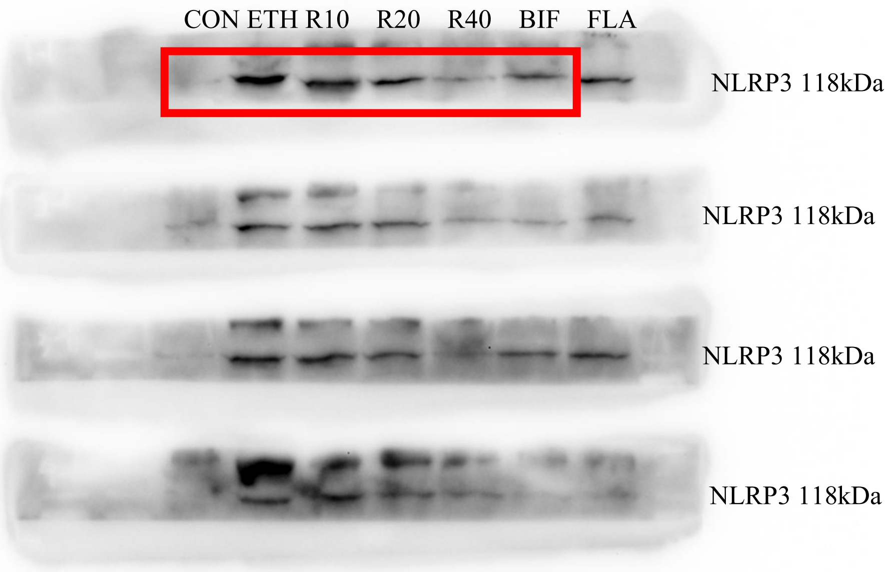
CON ETH R10 R20 R40 BIF FLA
NLRP3 118kDa
NLRP3 118kDa
NLRP3 118kDa
NLRP3 118kDa

In Figure.4

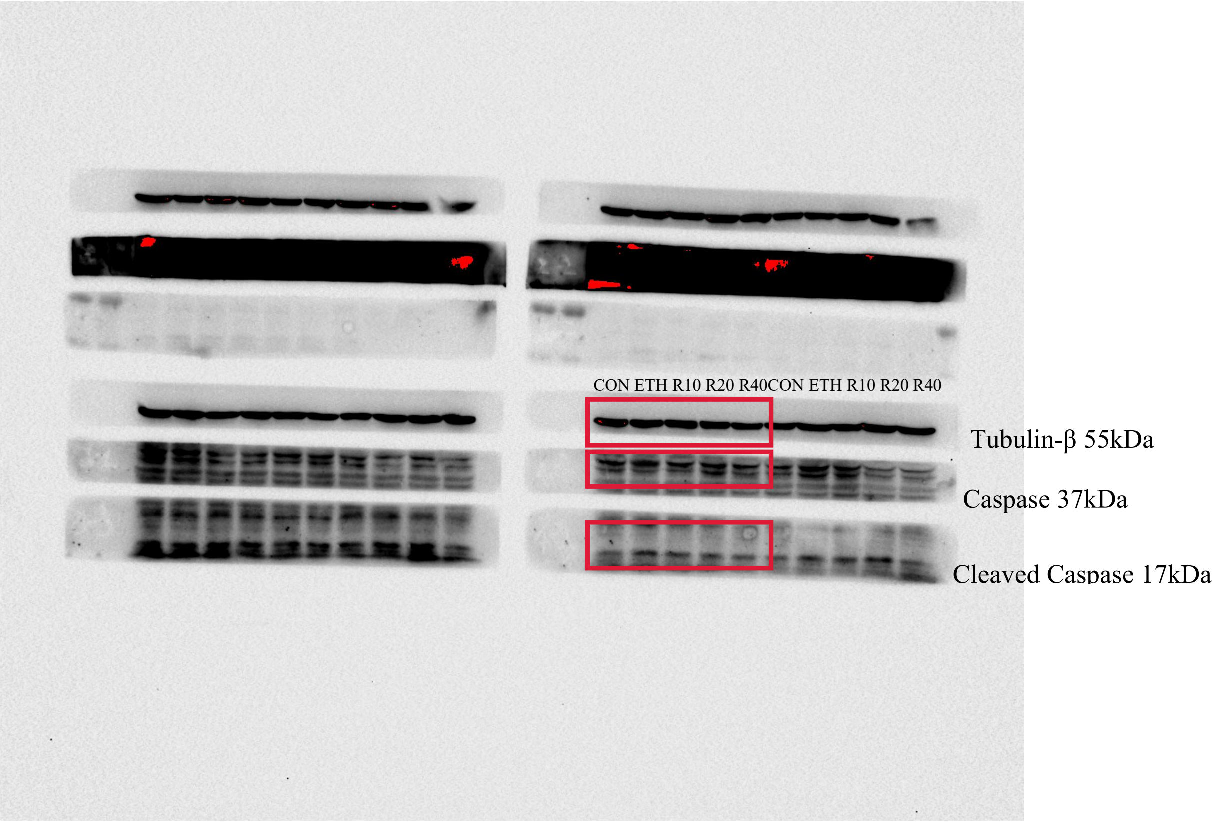
CON ETH R10 R20 R40CON ETH R10 R20 R40
Tubulin-β 55kDa
Caspase 37kDa
Cleaved Caspase 17kDa

In Figure.6

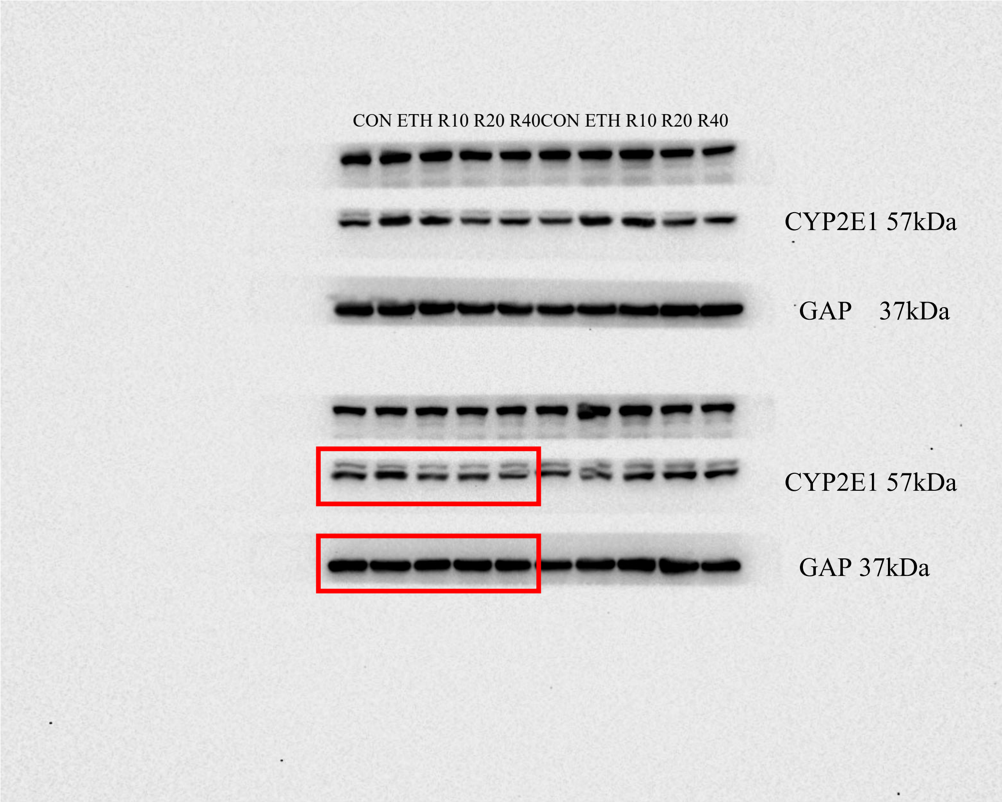

CON ETH R10 R20 R40CON ETH R10 R20 R40
CYP2E1 57kDa
GAP 37kDa
CYP2E1 57kDa
GAP 37kDa

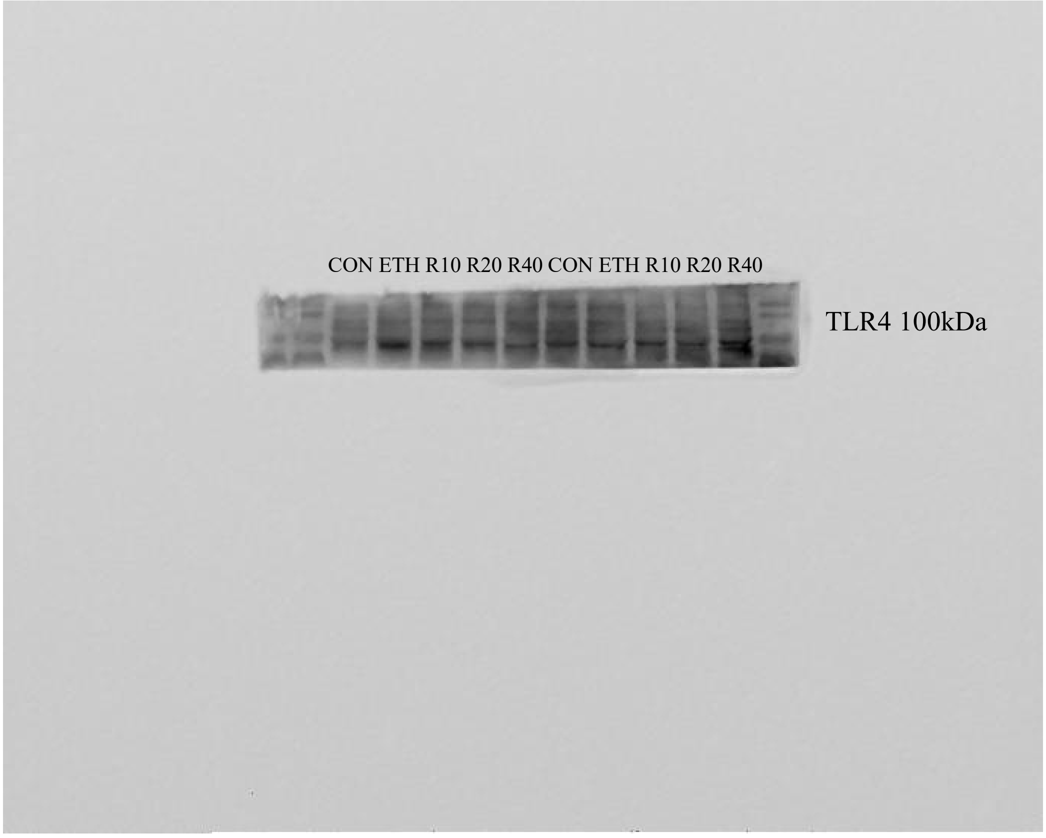

CON ETH R10 R20 R40 CON ETH R10 R20 R40
TLR4 100kDa

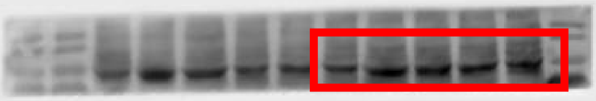

TLR4 100kDa

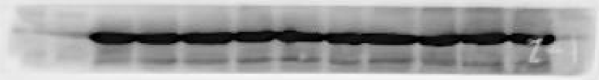

GAP 37kDa

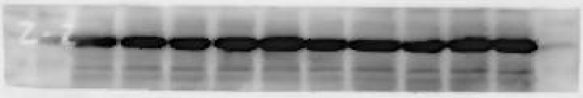

GAP 37kDa

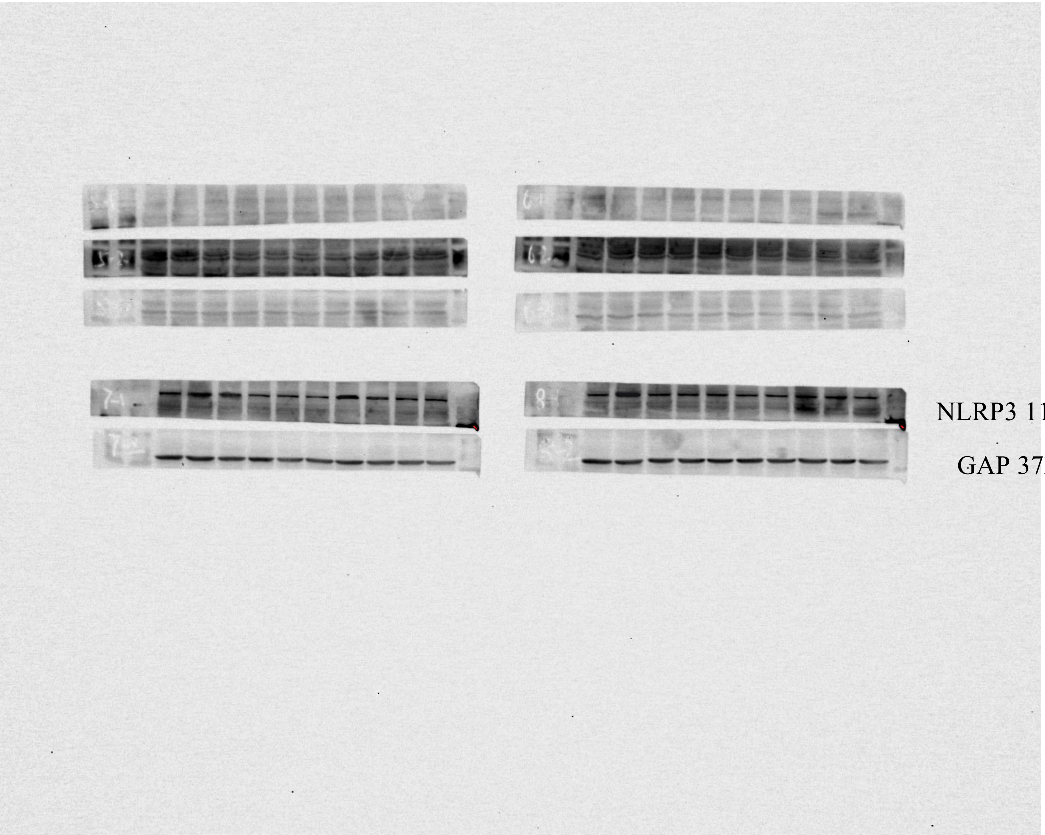

NLRP3 118kDa

GAP 37kDa

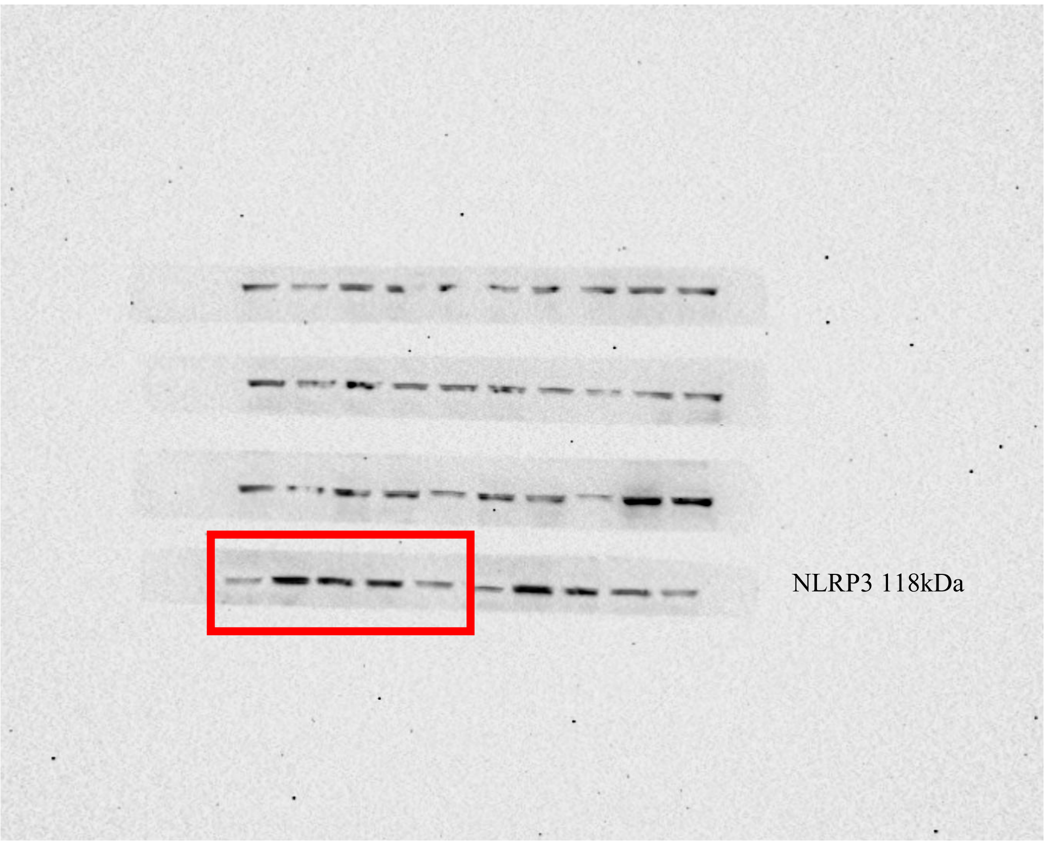

NLRP3 118kDa

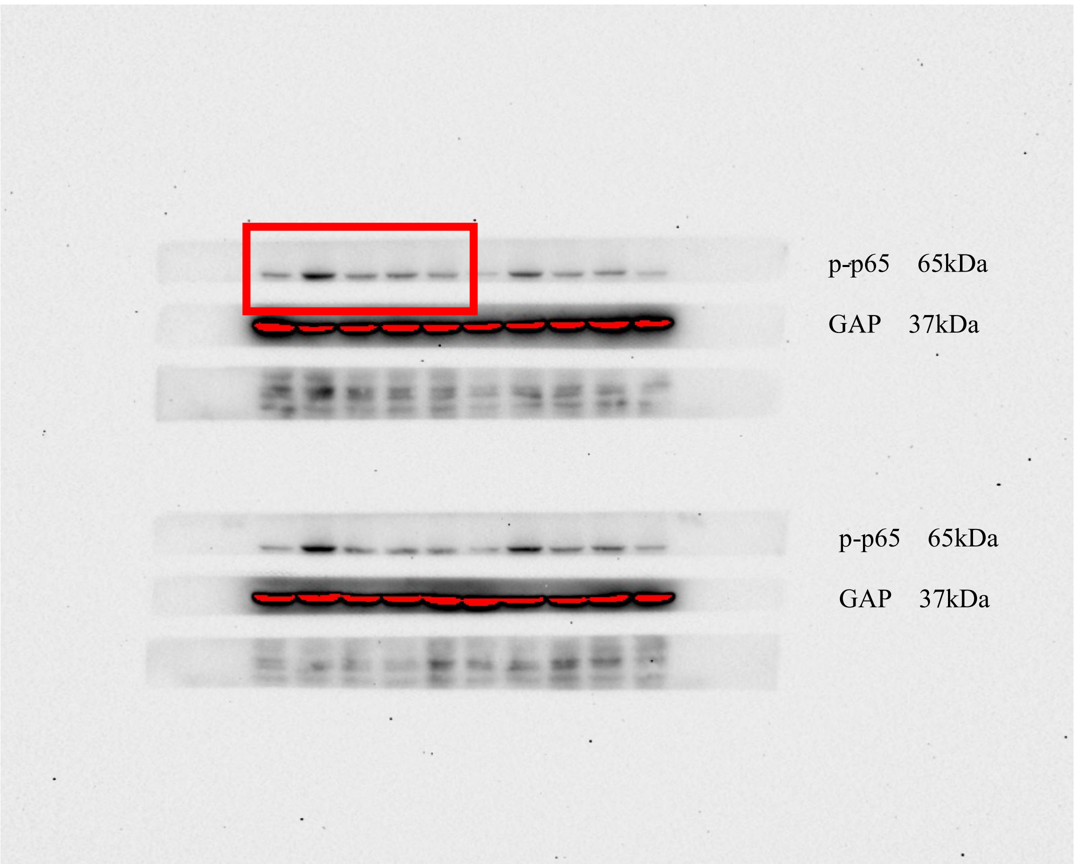
p-p65 65kDa
GAP 37kDa
p-p65 65kDa
GAP 37kDa

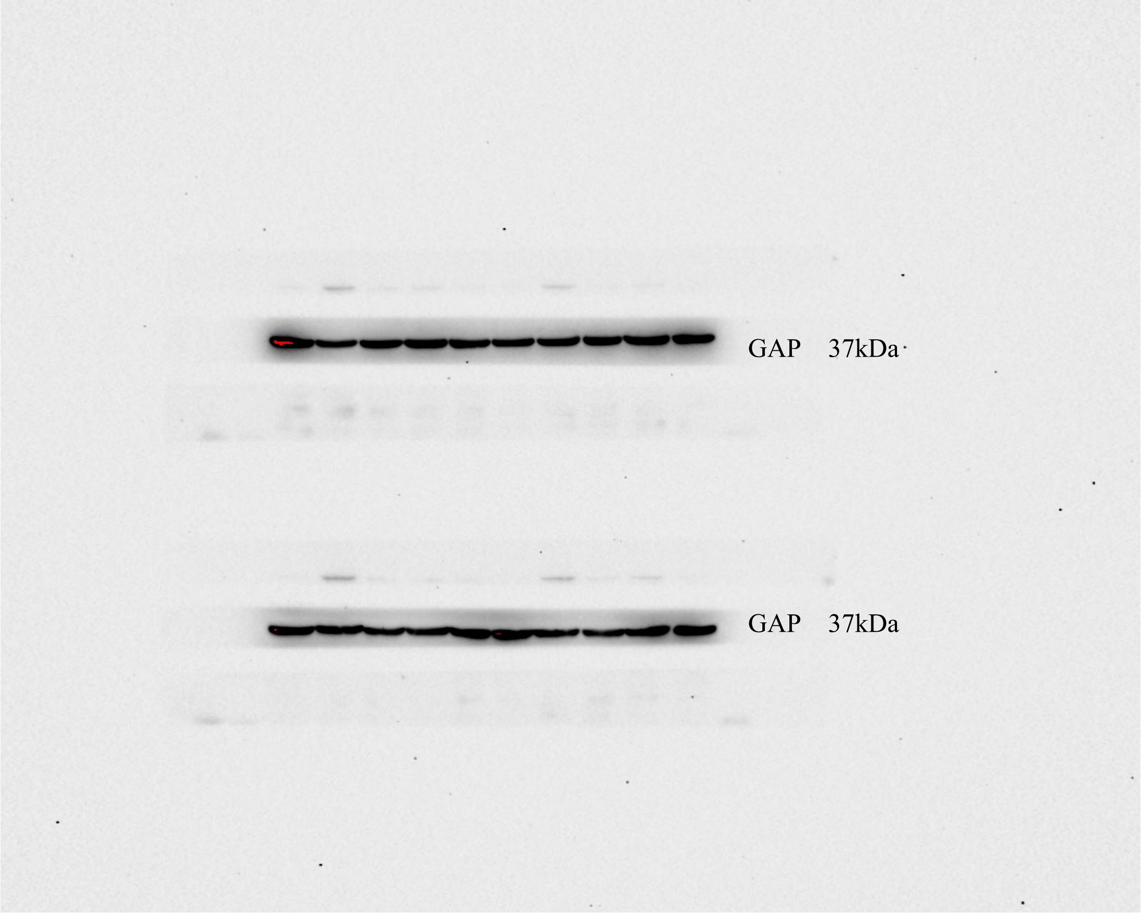
GAP 37kDa
GAP 37kDa

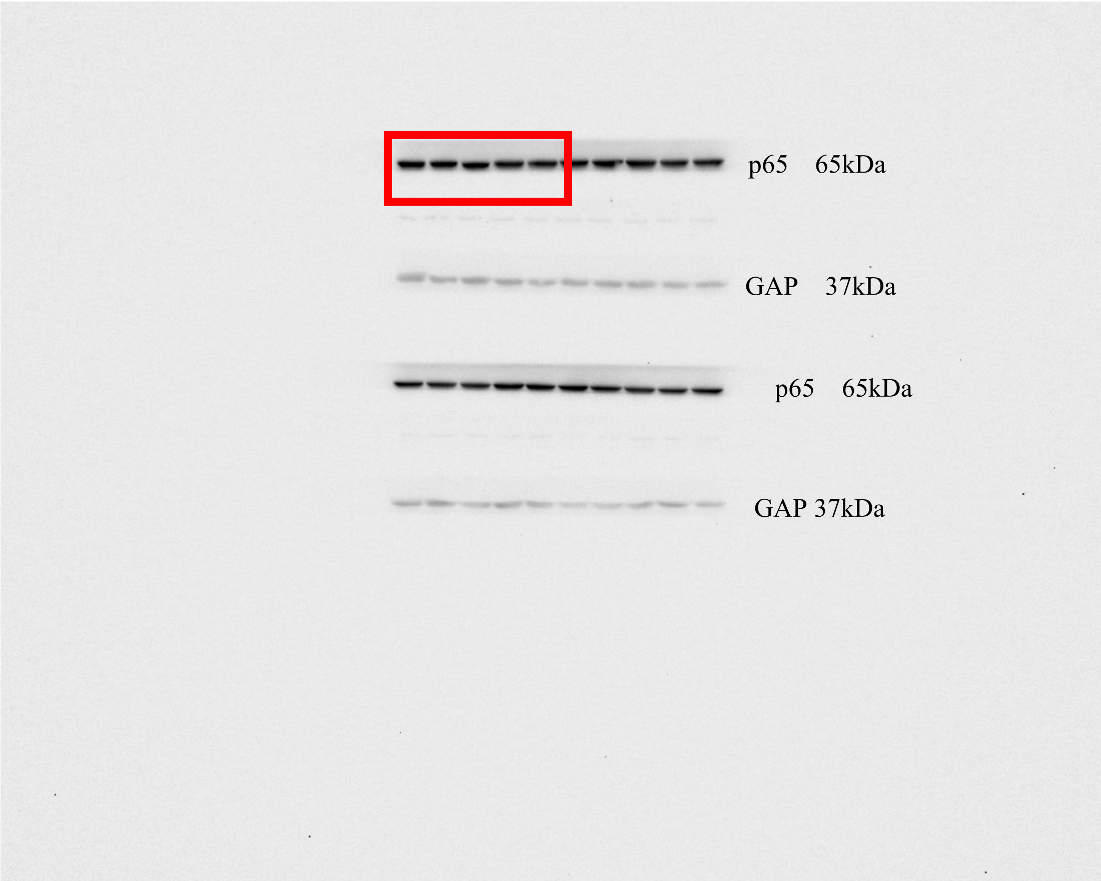
p65 65kDa
GAP 37kDa
p65 65kDa
GAP 37kDa

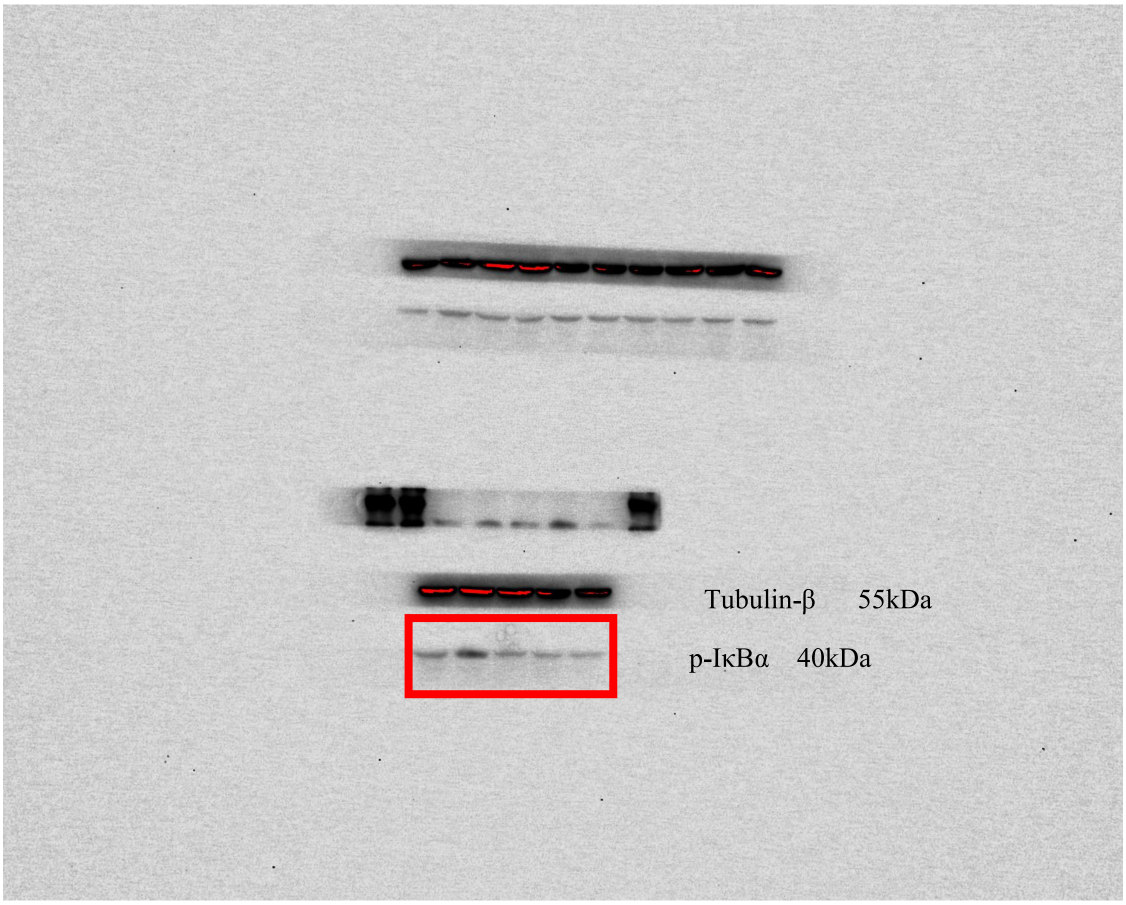
Tubulin-β 55kDa
p-IκBα 40kDa

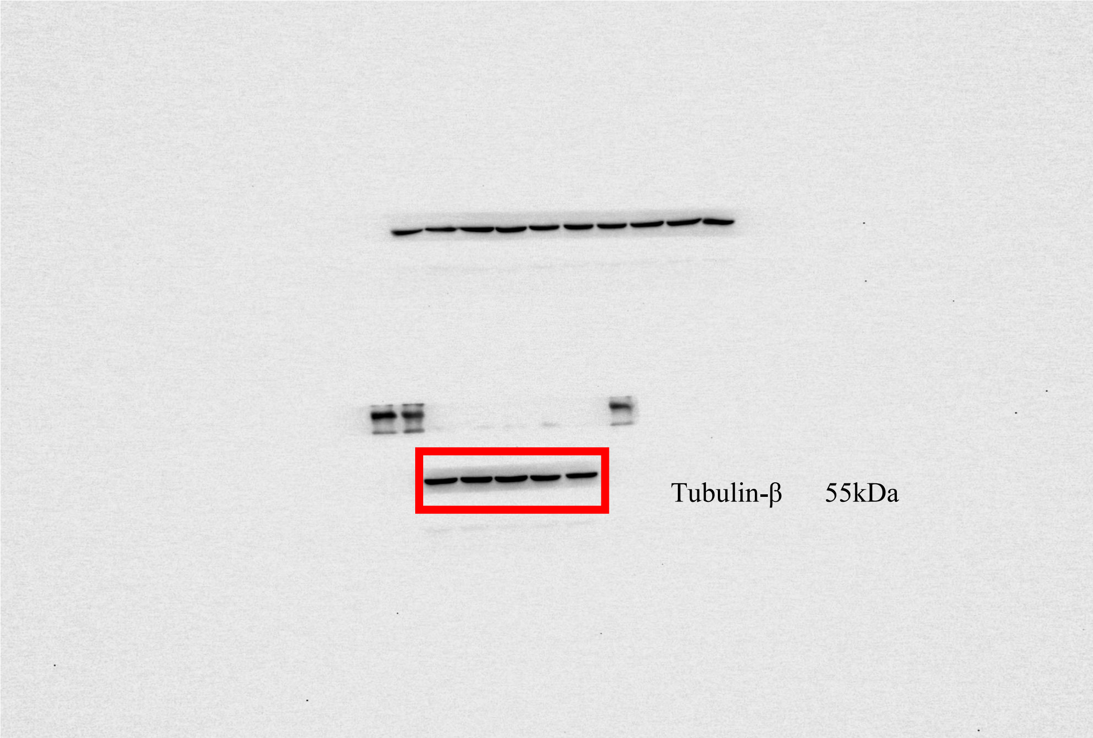
Tubulin-β 55kDa

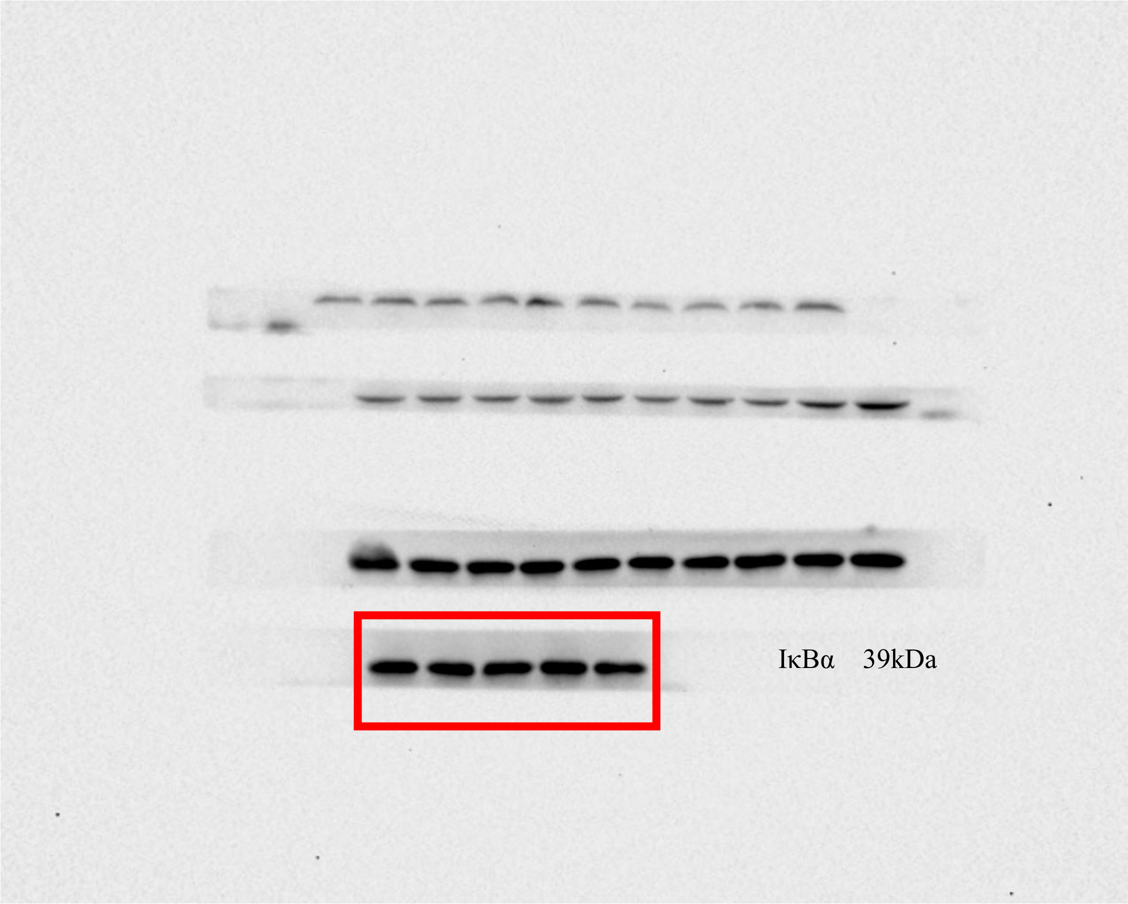
IκBα 39kDa

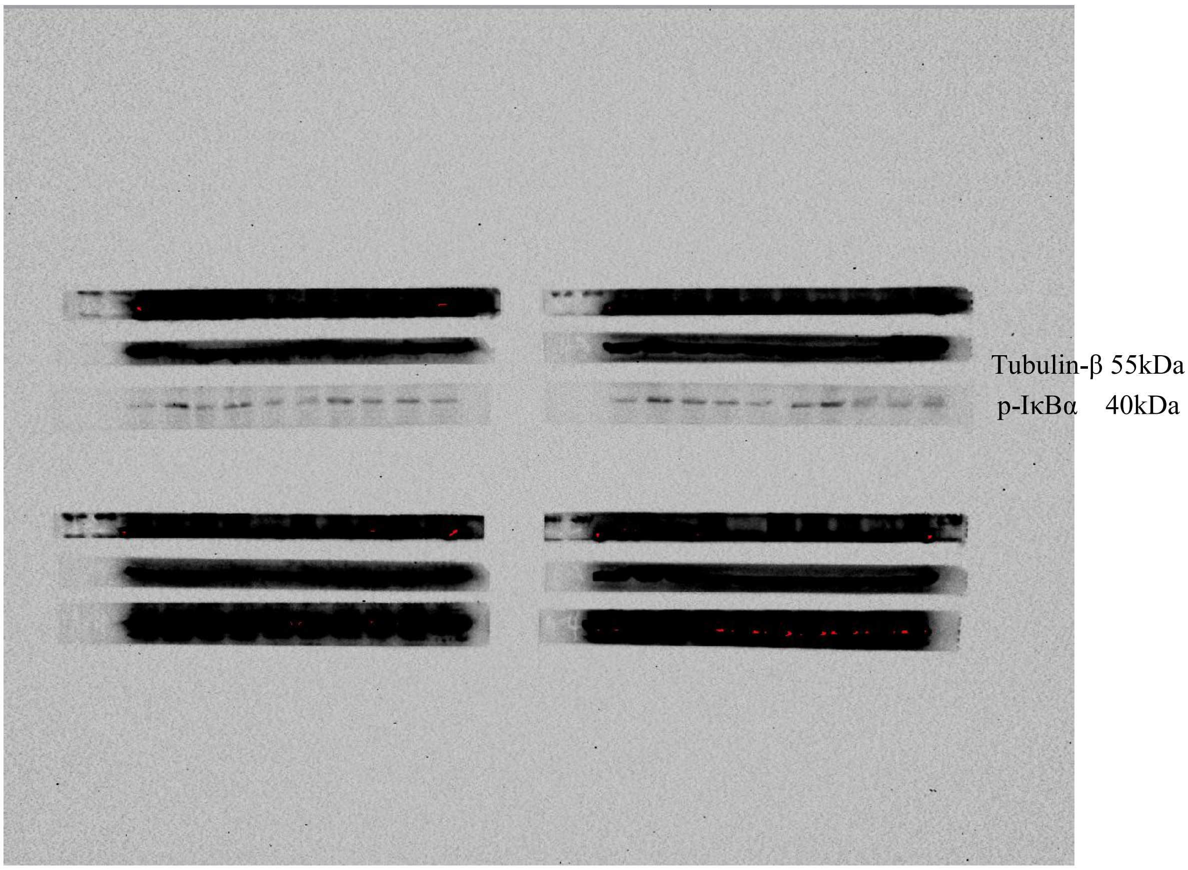
Tubulin-β 55kDa
p-IκBα 40kDa

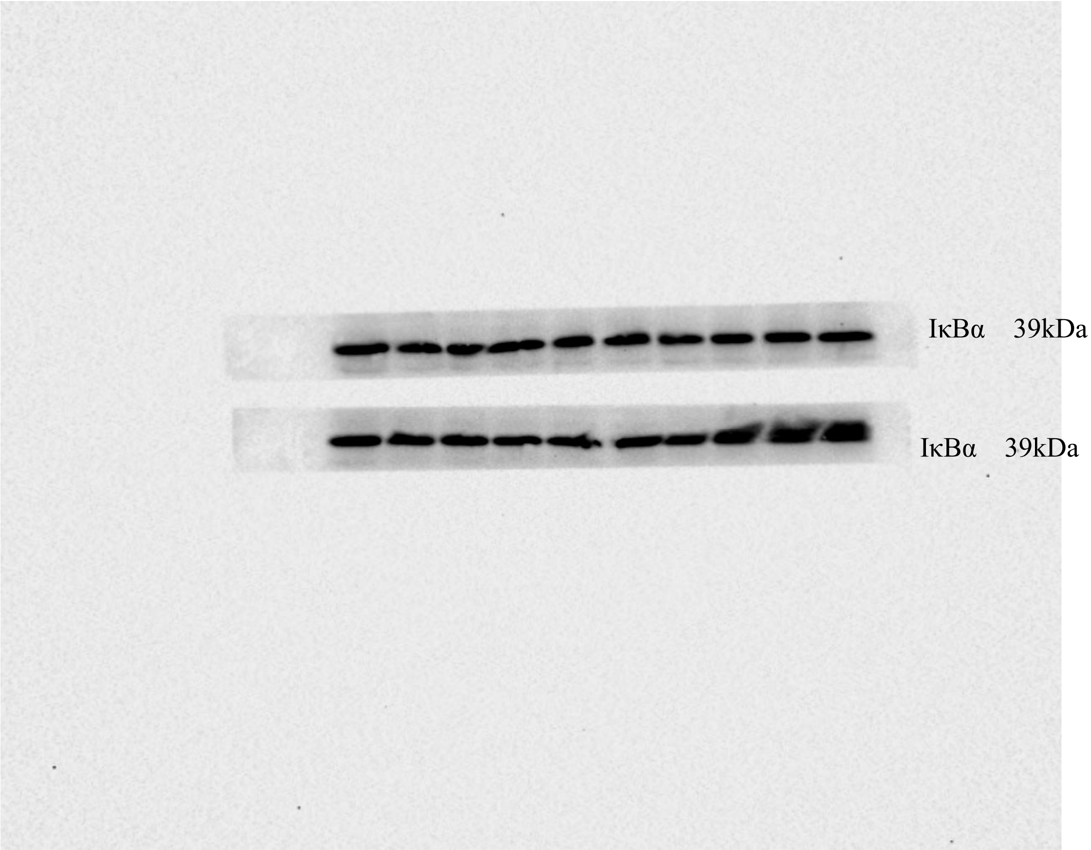
IκBα 39kDa
IκBα 39kDa

Original Files for Microscopy Images

HE (X200)

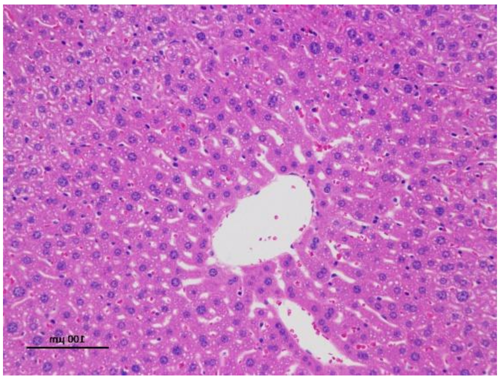

CON

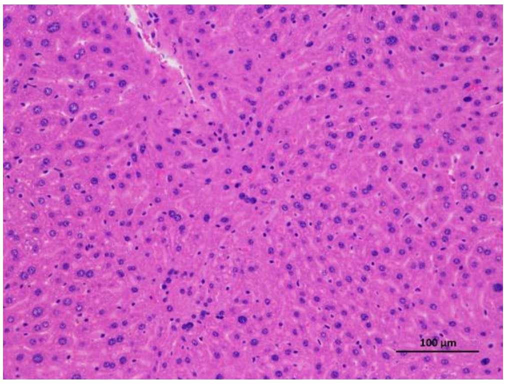

ETH

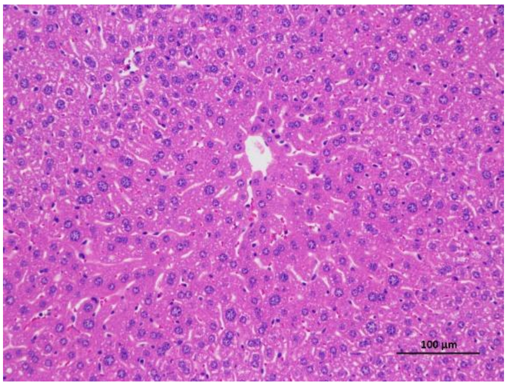

ROF10

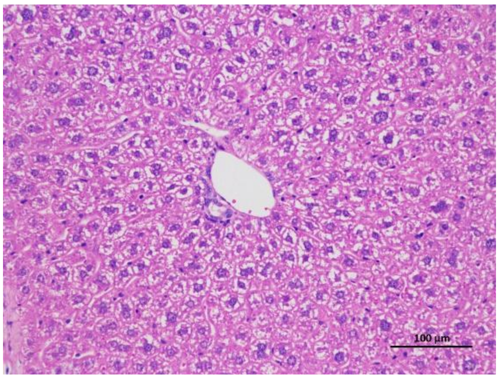

ROF20

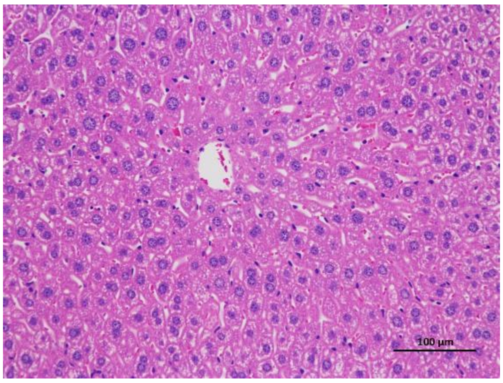

ROF40

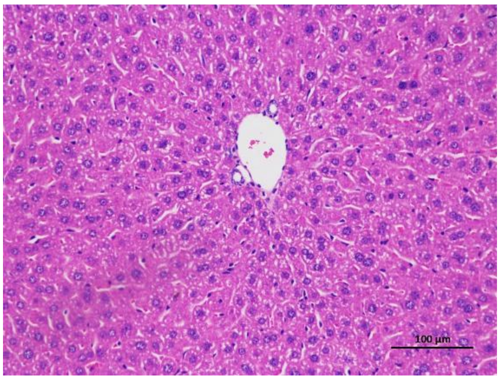

BIF

HE (X400)

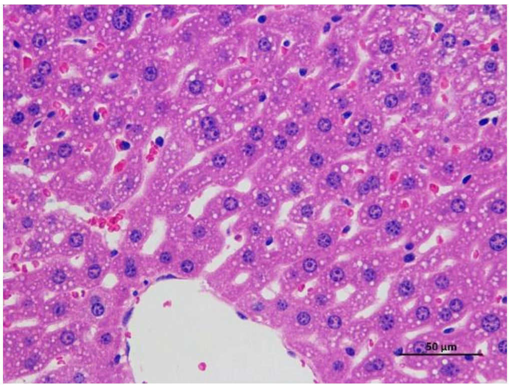

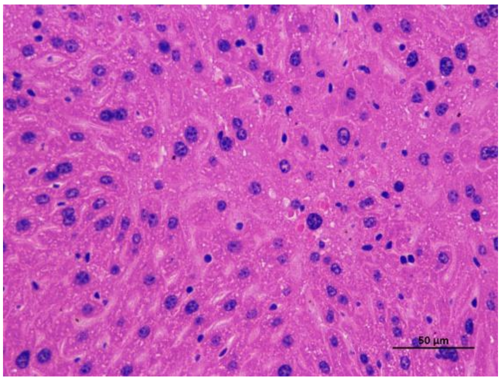

CON ETH

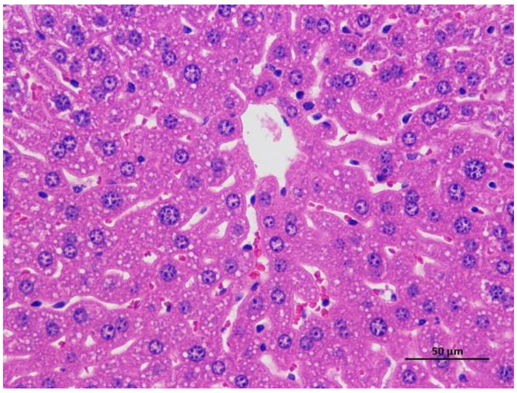

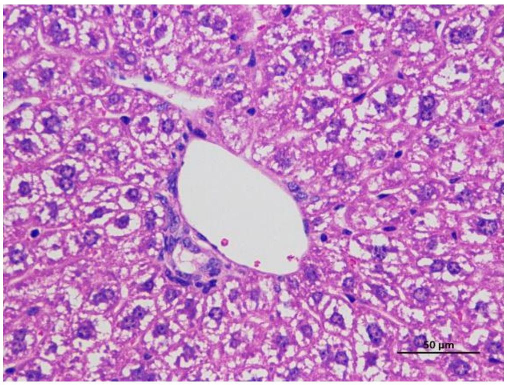

ROF10 ROF20

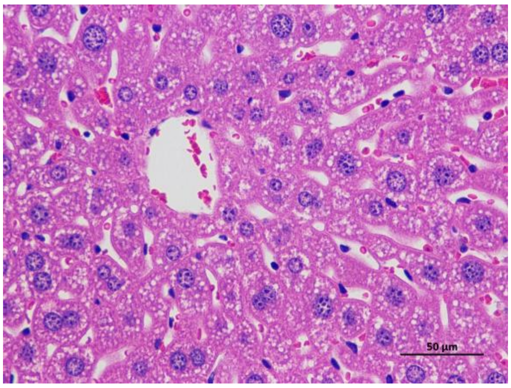

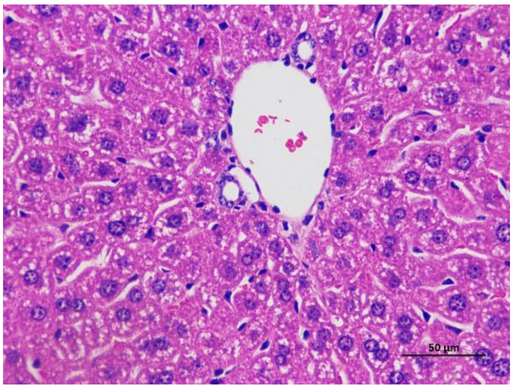

ROF40 BIF

IHC-CYP2E1 (X200)

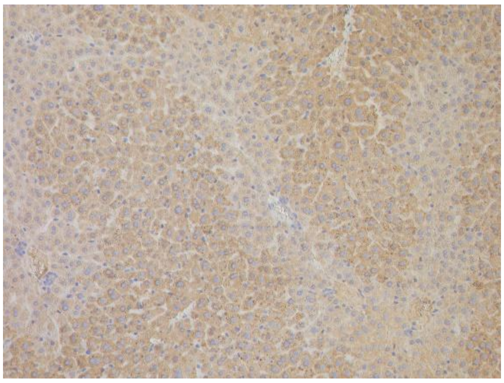

CON

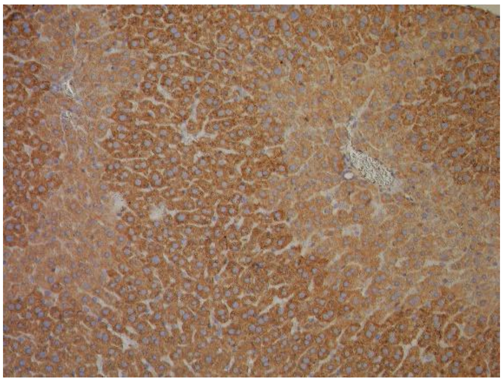

ETH

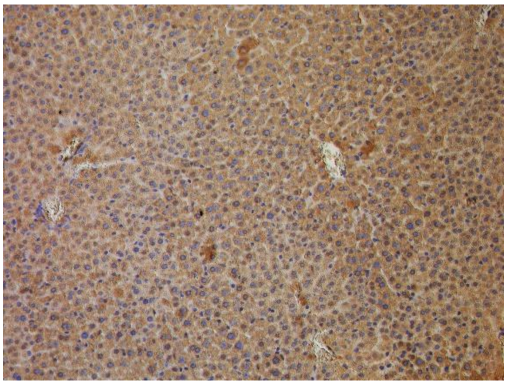

ROF10

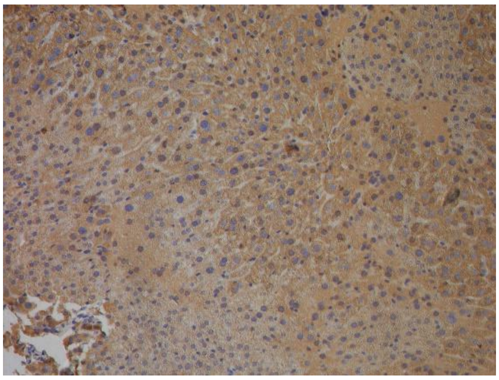

ROF20

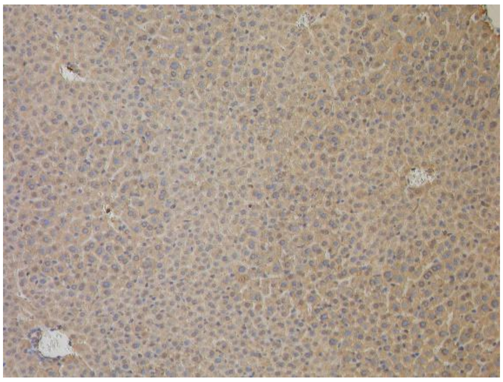

ROF40

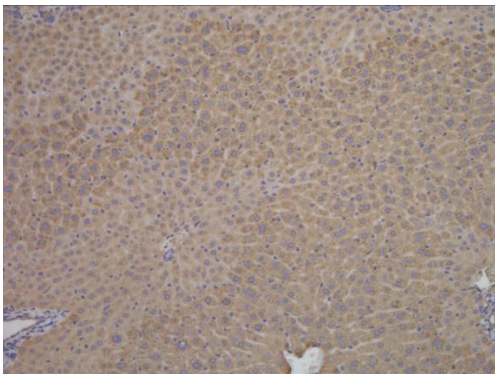

BIF

IHC-p65 (X200)

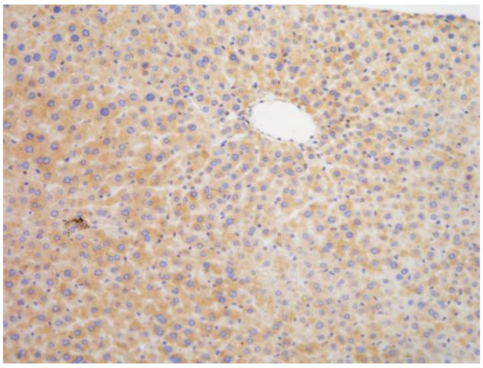

CON

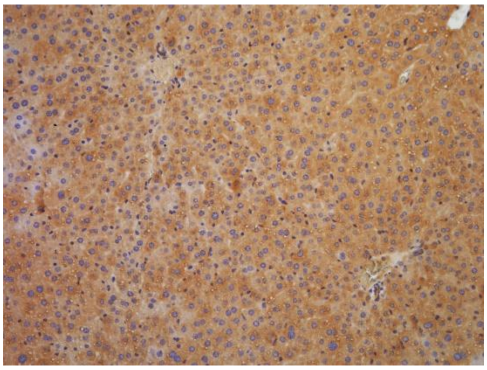

ETH

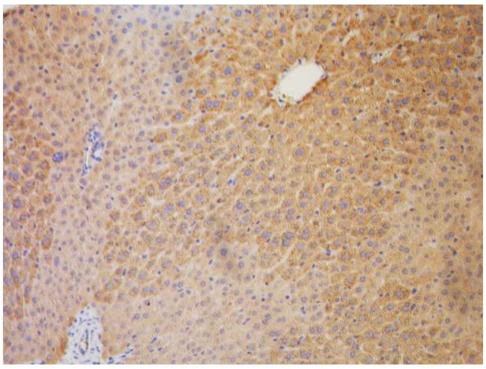

ROF10

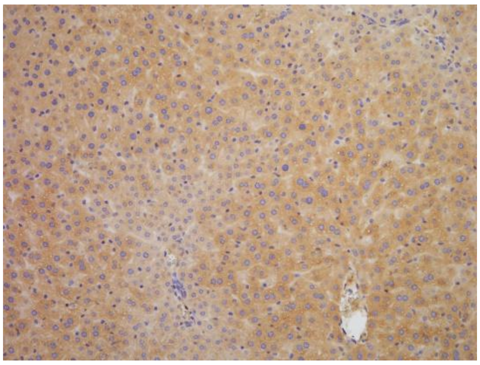

ROF20

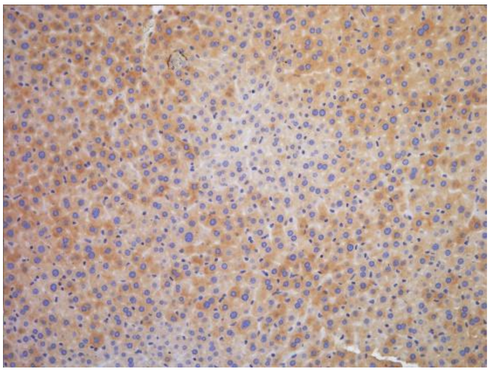

ROF40

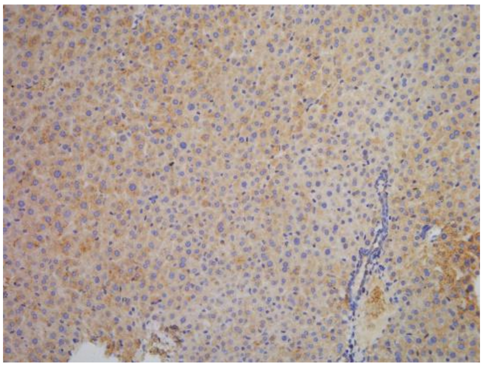

BIF

Institution: FC500
Protocol: p i user2018 00008640 882.PRO
Listmode Replay: Runtime Protocol
Analysis Date: 19-Nov-2019, 17:07:48
Settings File: PC9_Annexin V-Pi.PRO, 19-Nov-2019, 16:09:34
Listmode File: lo2-C user2018 00008647 889.LMD

Run Date: 19-Nov-19, 16:10:29
Sample ID: lo2-C
User ID: user2018
Acquisition Time/Events: 15.4s / 10000 (PROTOCOL)

Instrument SN: AM18110 Software Version: CXP 2.3

(F1)[Ungated] lo2-C user2018 00008647 889.LMD : SS Lin/FS Li

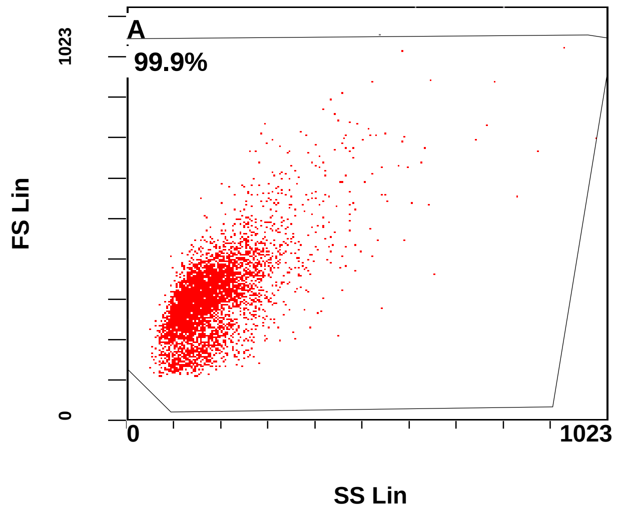

(F1)[A] lo2-C user2018 00008647 889.LMD : FL3 Log - ADC

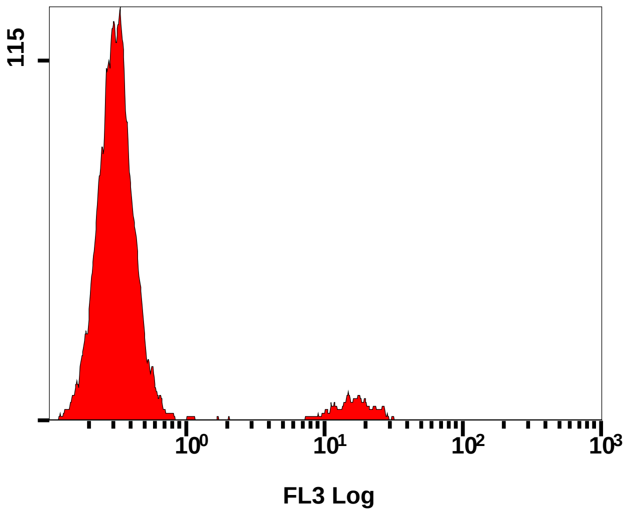

(F1)[A] lo2-C user2018 00008647 889.LMD : FL1 Log - AD

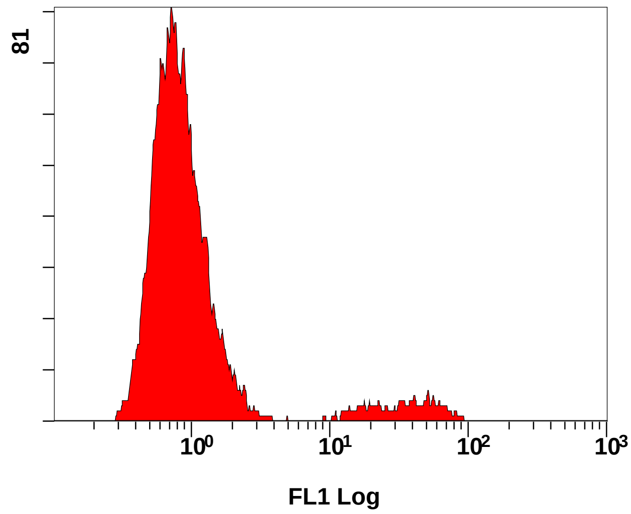

(F1)[A] lo2-C user2018 00008647 889.LMD : FL1 Log/FL3 Log - ADC

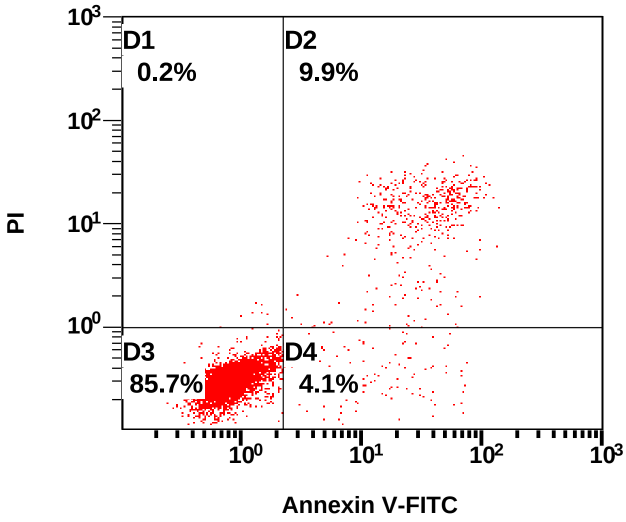

Institution: FC500
Protocol: p i user2018 00008640 882.PRO
Listmode Replay: Runtime Protocol
Analysis Date: 19-Nov-2019, 17:08:41
Settings File: PC9_Annexin V-Pi.PRO, 19-Nov-2019, 16:10:44
Listmode File: lo2-M user2018 00008648 890.LMD

Run Date: 19-Nov-19, 16:11:36
Sample ID: lo2-M
User ID: user2018
Acquisition Time/Events: 18.2s / 10000 (PROTOCOL)

Instrument SN: AM18110 Software Version: CXP 2.3

(F1)[Ungated] lo2-M user2018 00008648 890.LMD : SS Lin/FS Li

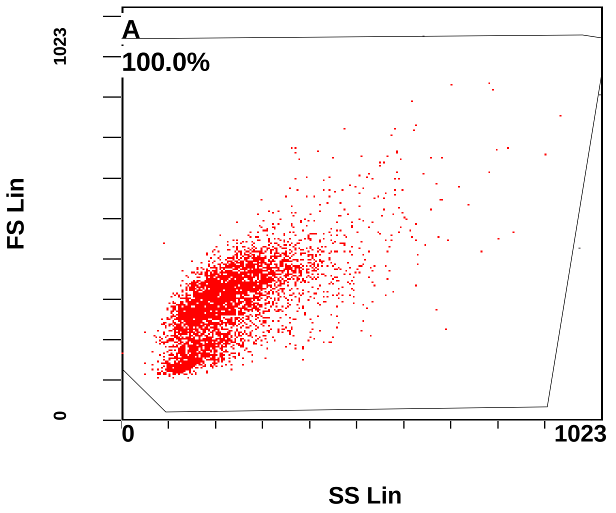

(F1)[A] lo2-M user2018 00008648 890.LMD : FL3 Log - ADC

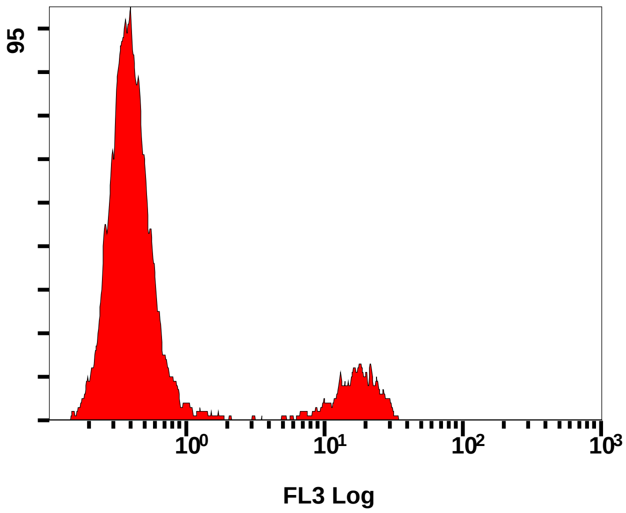

(F1)[A] lo2-M user2018 00008648 890.LMD : FL1 Log - A

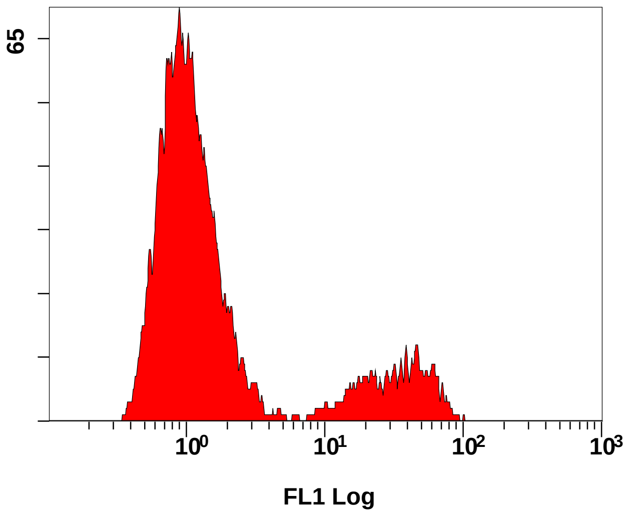

(F1)[A] lo2-M user2018 00008648 890.LMD : FL1 Log/FL3 Log - ADC

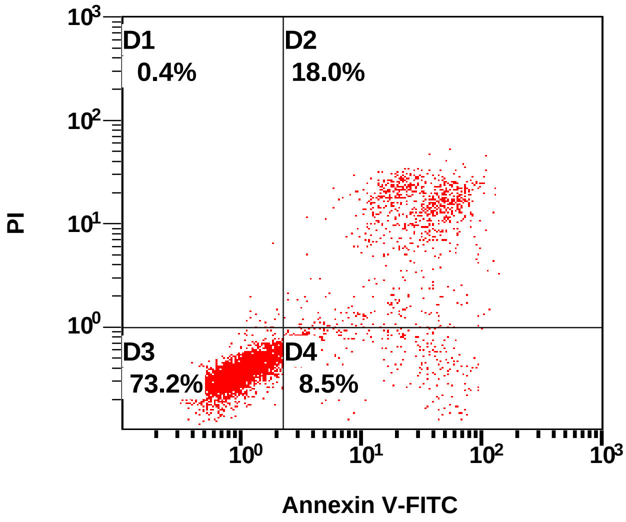

Institution: FC500
Protocol: p i user2018 00008640 882.PRO
Listmode Replay: Runtime Protocol
Analysis Date: 19-Nov-2019, 17:09:21
Settings File: PC9_Annexin V-Pi.PRO, 19-Nov-2019, 16:11:55
Listmode File: LO2-25 user2018 00008649 891.LMD

Run Date: 19-Nov-19, 16:12:49
Sample ID: LO2-25
User ID: user2018
Acquisition Time/Events: 20.3s / 10000 (PROTOCOL)

Instrument SN: AM18110 Software Version: CXP 2.3

(F1)[Ungated] LO2-25 user2018 00008649 891.LMD : SS Lin/FS

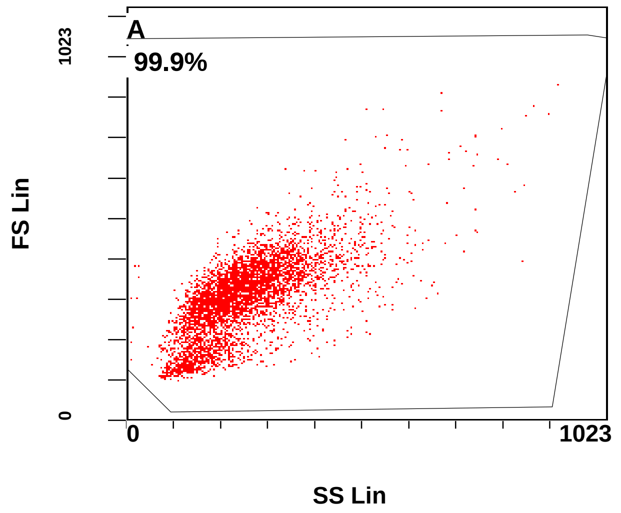

(F1)[A]LO2-25 user2018 00008649 891.LMD : FL3 Log - ADC

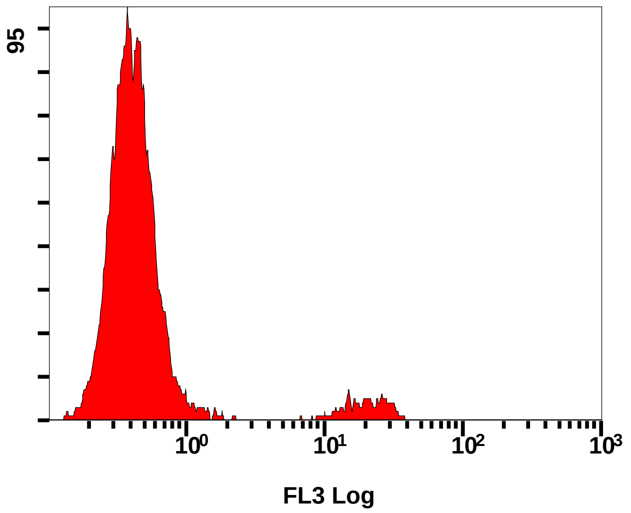

(F1)[A] LO2-25 user2018 00008649 891.LMD : FL1 Lo

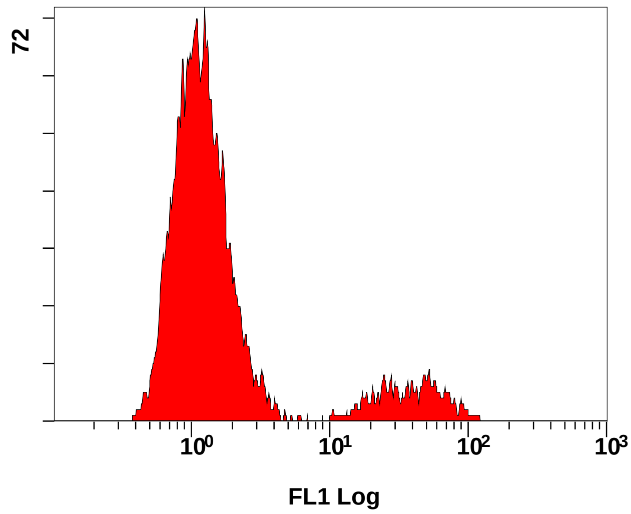

LO2-25 user2018 00008649 891.LMD : FL1 Log/FL3 Log - ADC

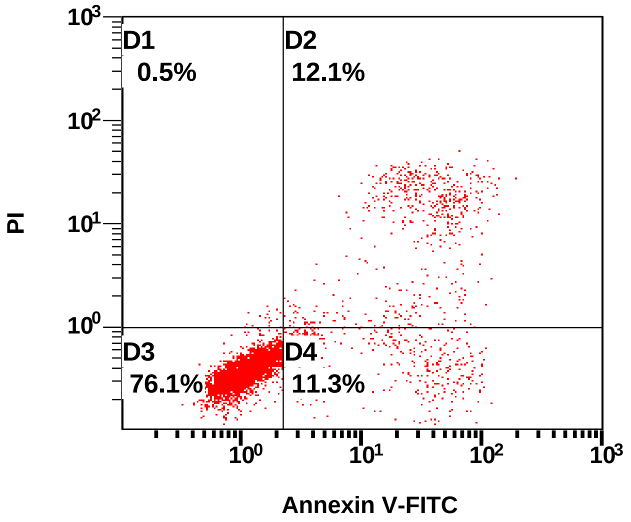

Institution: FC500
Protocol: p i user2018 00008640 882.PRO
Listmode Replay: Runtime Protocol
Analysis Date: 19-Nov-2019, 17:10:20
Settings File: PC9_Annexin V-Pi.PRO, 19-Nov-2019, 16:14:16
Listmode File: LO2-50 user2018 00008651 893.LMD

Run Date: 19-Nov-19, 16:15:02
Sample ID: LO2-50
User ID: user2018
Acquisition Time/Events: 16.5s / 10000 (PROTOCOL)

Instrument SN: AM18110 Software Version: CXP 2.3

(F1)[Ungated] LO2-50 user2018 00008651 893.LMD : SS Lin/FS L

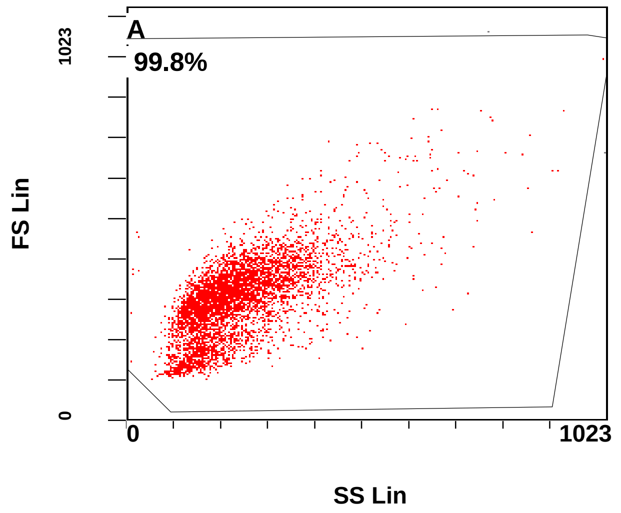

(F1)[A] LO2-50 user2018 00008651 893.LMD : FL3 Log - ADC

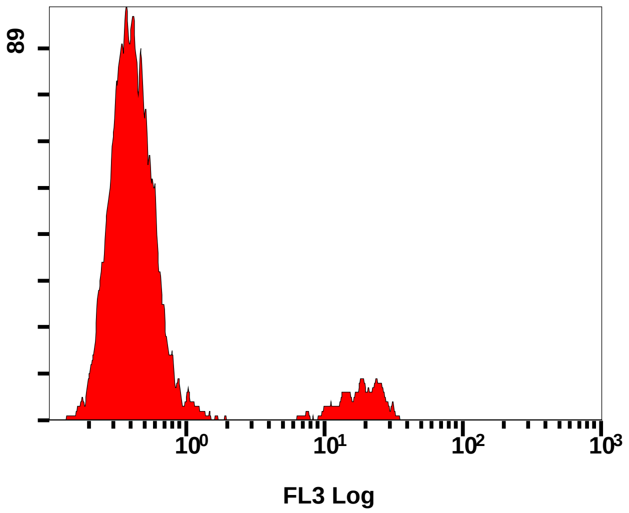

(F1)[A] LO2-50 user2018 00008651 893.LMD : FL1 Log - /

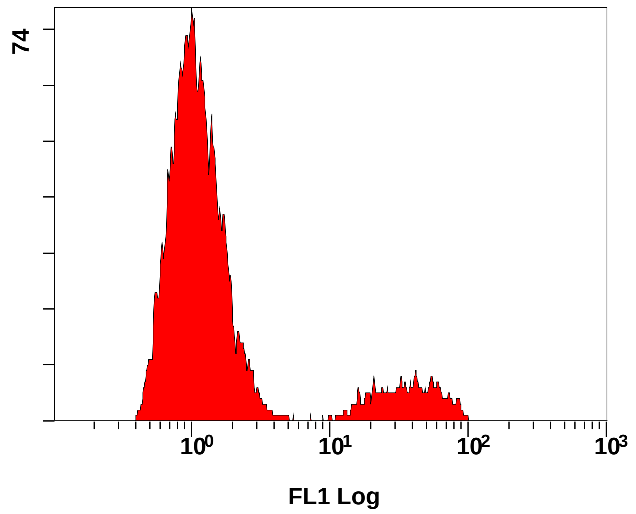

(F1)[A] LO2-50 user2018 00008651 893.LMD : FL1 Log/FL3 Log - ADC

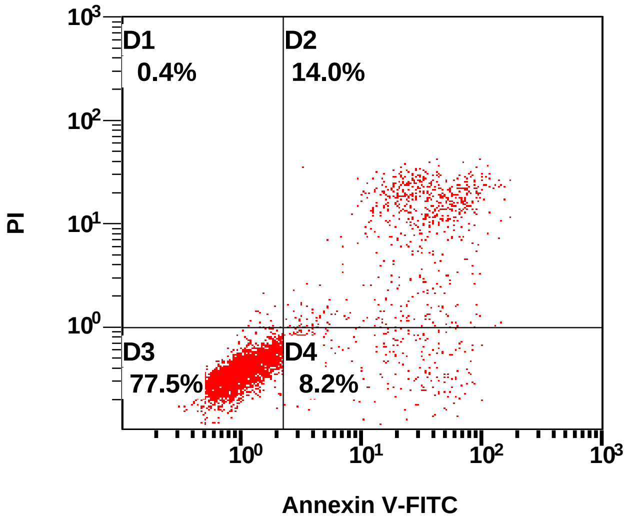

Institution: FC500
Protocol: p i user2018 00008640 882.PRO
Listmode Replay: Runtime Protocol
Analysis Date: 19-Nov-2019, 17:09:50
Settings File: PC9_Annexin V-Pi.PRO, 19-Nov-2019, 16:13:10
Listmode File: LO2-100 user2018 00008650 892.LMD

Run Date: 19-Nov-19, 16:13:59
Sample ID: LO2-100
User ID: user2018
Acquisition Time/Events: 16.6s / 10000 (PROTOCOL)

Instrument SN: AM18110 Software Version: CXP 2.3

(F1)[Ungated] LO2-100 user2018 00008650 892.LMD : SS Lin/FS l

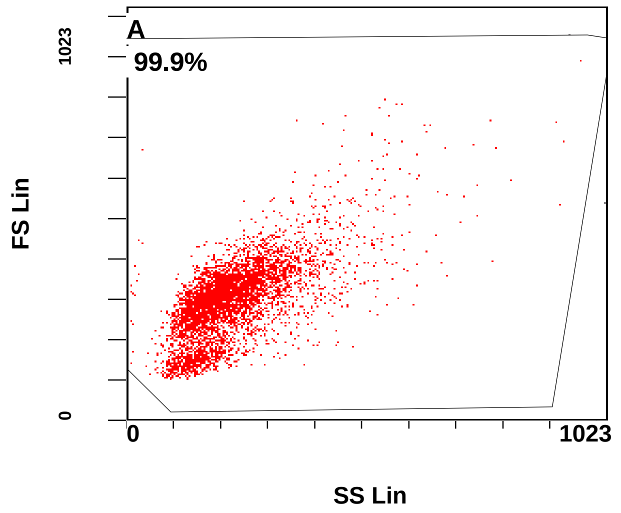

(F1)[A] LO2-100 user2018 00008650 892.LMD : FL3 Log - ADC

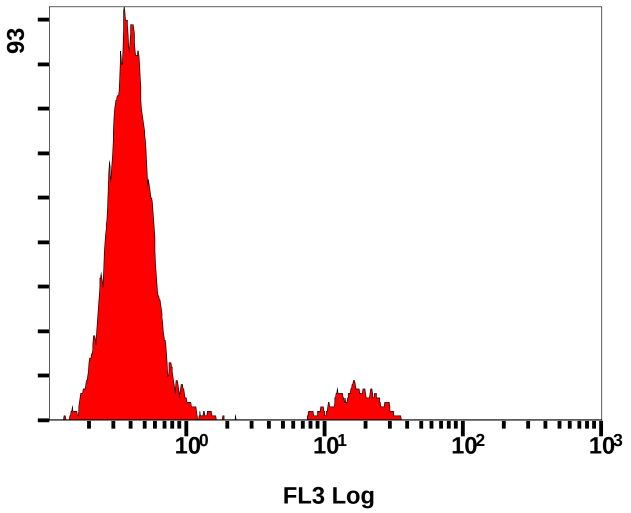

(F1)[A] LO2-100 user2018 00008650 892.LMD : FL1 Log -

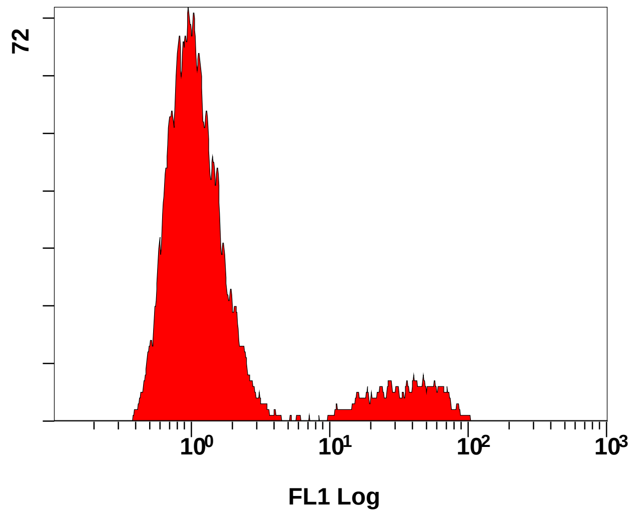

(F1)[A] LO2-100 user2018 00008650 892.LMD : FL1 Log/FL3 Log - ADC

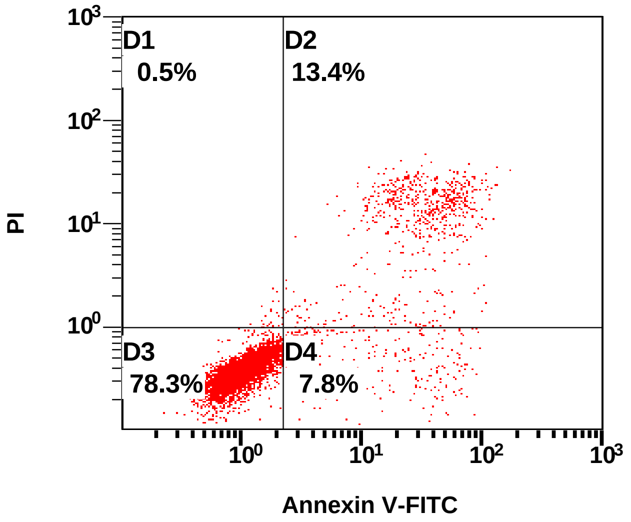

Institution: FC500
Protocol: LO2-50 user2018 00008652 894.PRO
Listmode Replay: New Protocol
Analysis Date: 29-Nov-2019, 15:30:32
Settings File: PC9_Annexin V-Pi.PRO, 19-Nov-2019, 16:09:34
Listmode File: lo2-C user2018 00008647 889.LMD

Run Date: 19-Nov-19, 16:10:29
Sample ID: lo2-C
User ID: user2018
Acquisition Time/Events: 15.4s / 10000 (PROTOCOL)

Instrument SN: AM18110 Software Version: CXP 2.3

(F1)[Ungated] lo2-C user2018 00008647 889.LMD : SS Lin/FS Li

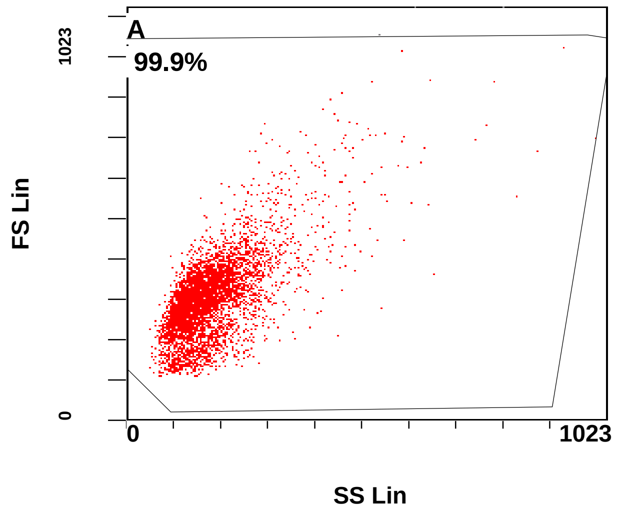

(F1)[A] lo2-C user2018 00008647 889.LMD : FL3 Log - ADC

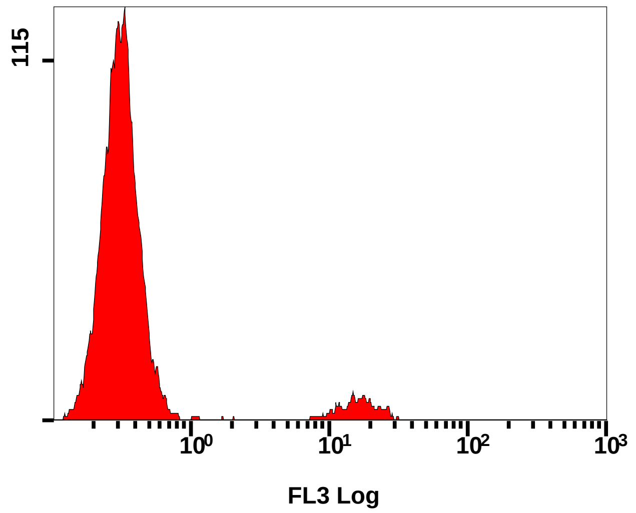

(F1)[A] lo2-C user2018 00008647 889.LMD : FL1 Log - AD

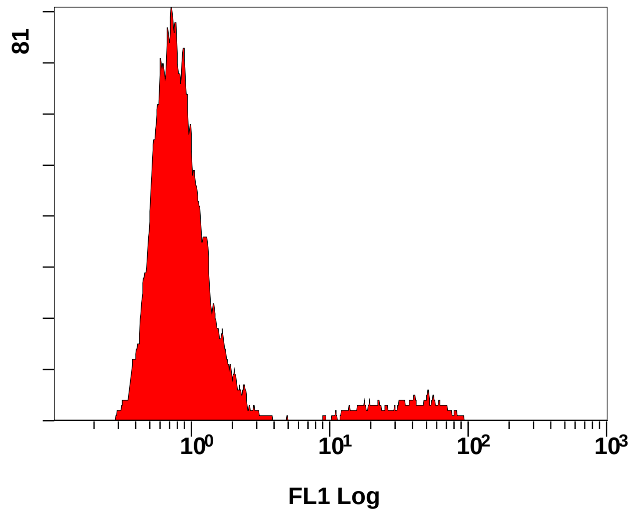

(F1)[A] lo2-C user2018 00008647 889.LMD : FL1 Log/FL3 Log - ADC

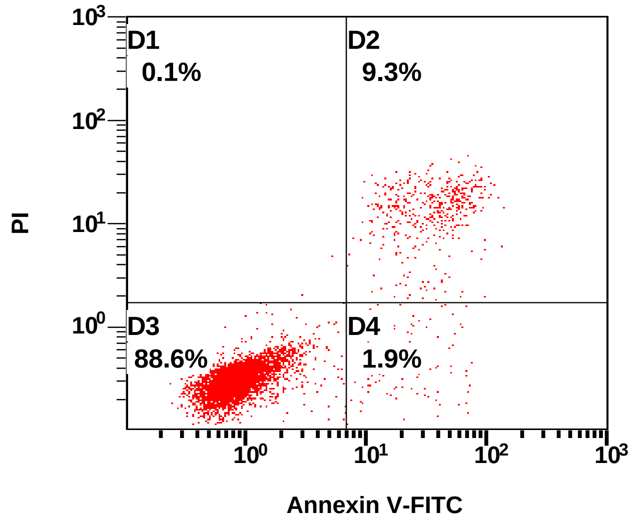

LO2-50 user2018 00008652 894.PRO : CXP

Institution: FC500
Protocol: LO2-50 user2018 00008652 894.PRO
Listmode Replay: New Protocol
Analysis Date: 29-Nov-2019, 15:31:08
Settings File: PC9_Annexin V-Pi.PRO, 19-Nov-2019, 16:10:44
Listmode File: lo2-M user2018 00008648 890.LMD

Run Date: 19-Nov-19, 16:11:36
Sample ID: lo2-M
User ID: user2018
Acquisition Time/Events: 18.2s / 10000 (PROTOCOL)

Instrument SN: AM18110 Software Version: CXP 2.3

(F1)[Ungated] lo2-M user2018 00008648 890.LMD : SS Lin/FS Li

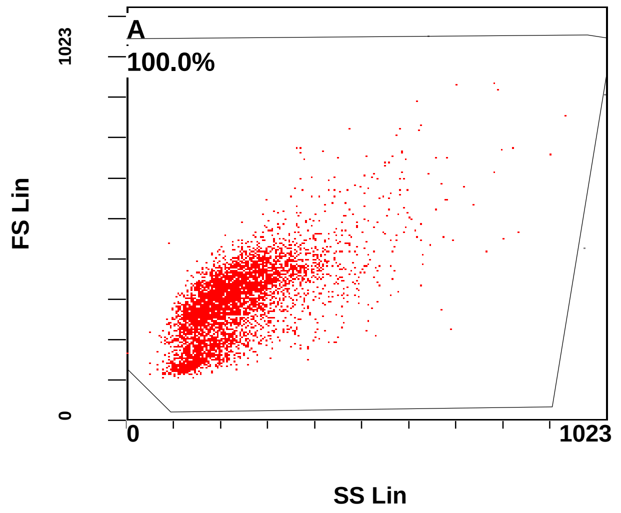

(F1)[A] lo2-M user2018 00008648 890.LMD : FL3 Log - ADC

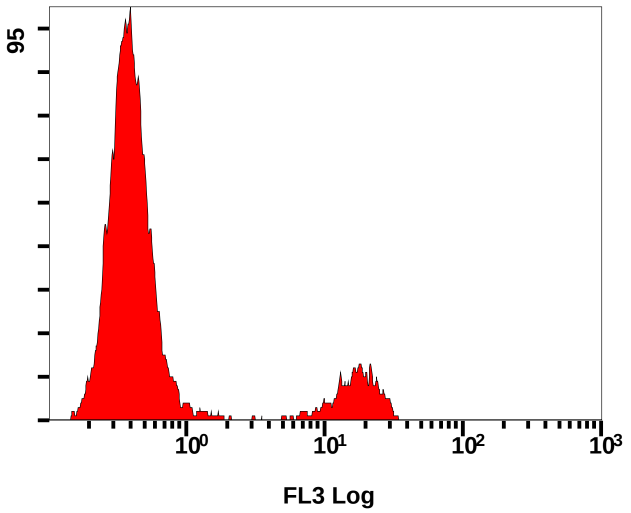

(F1)[A] lo2-M user2018 00008648 890.LMD : FL1 Log - AI

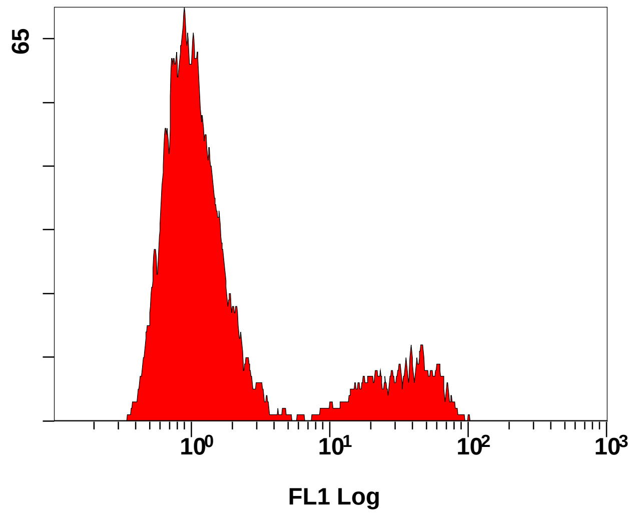

(F1)[A] lo2-M user2018 00008648 890.LMD : FL1 Log/FL3 Log - ADC

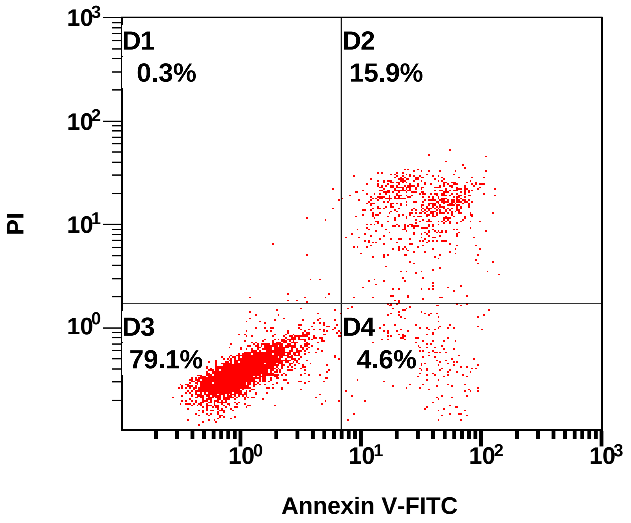

LO2-50 user2018 00008652 894.PRO : CXP

Institution: FC500
Protocol: LO2-50 user2018 00008652 894.PRO
Listmode Replay: New Protocol
Analysis Date: 29-Nov-2019, 15:32:18
Settings File: PC9_Annexin V-Pi.PRO, 19-Nov-2019, 16:16:42
Listmode File: LO2-25 user2018 00008653 895.LMD

Run Date: 19-Nov-19, 16:17:25
Sample ID: LO2-25
User ID: user2018
Acquisition Time/Events: 18.7s / 10000 (PROTOCOL)

Instrument SN: AM18110 Software Version: CXP 2.3

(F1)[Ungated] LO2-25 user2018 00008653 895.LMD : SS Lin/FS L

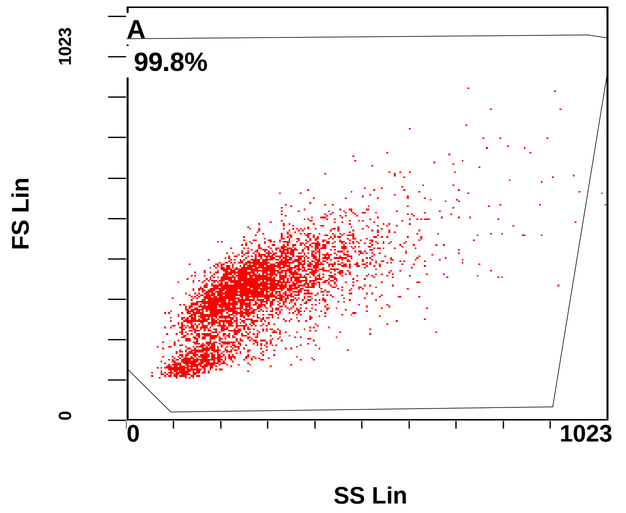

(F1)[A] LO2-25 user2018 00008653 895.LMD : FL3 Log - ADC

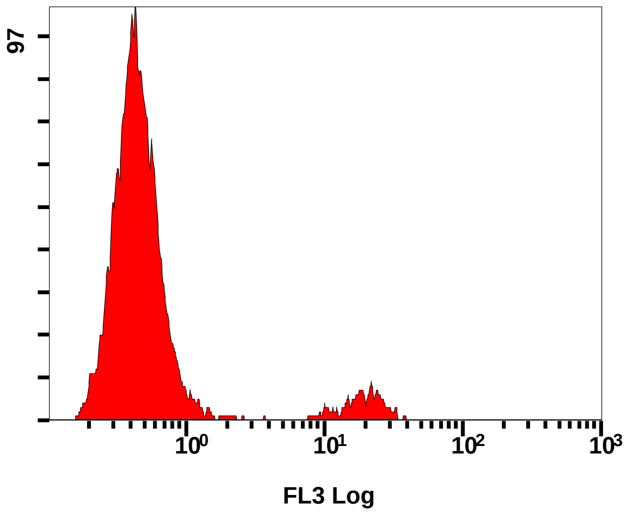

(F1)[A] LO2-25 user2018 00008653 895.LMD : FL1 Log - A

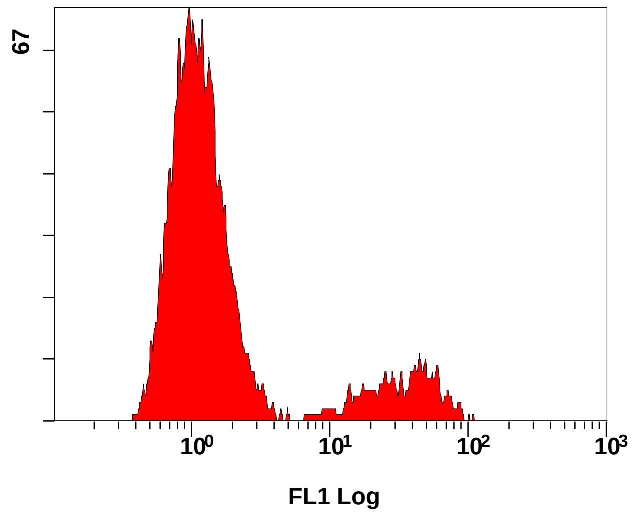

(F1)[A] LO2-25 user2018 00008653 895.LMD : FL1 Log/FL3 Log - ADC

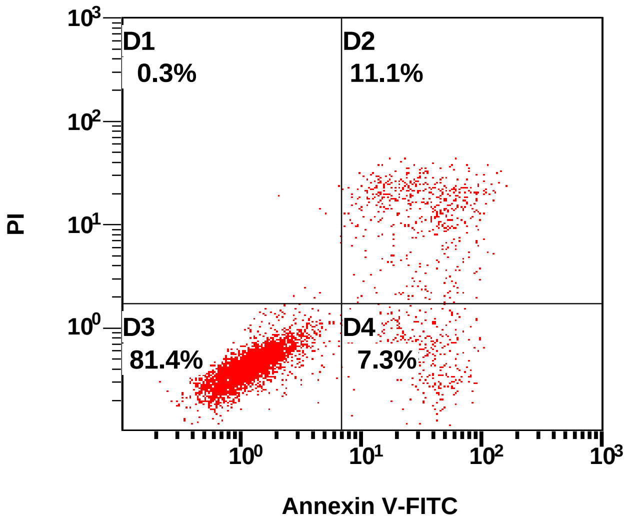

LO2-50 user2018 00008652 894.PRO : CXP

Institution: FC500
Protocol: LO2-50 user2018 00008652 894.PRO
Listmode Replay: New Protocol
Analysis Date: 29-Nov-2019, 15:33:22
Settings File: PC9_Annexin V-Pi.PRO, 19-Nov-2019, 16:14:16
Listmode File: LO2-50 user2018 00008651 893.LMD

Run Date: 19-Nov-19, 16:15:02
Sample ID: LO2-50
User ID: user2018
Acquisition Time/Events: 16.5s / 10000 (PROTOCOL)

Instrument SN: AM18110 Software Version: CXP 2.3

(F1)[Ungated] LO2-50 user2018 00008651 893.LMD : SS Lin/FS L

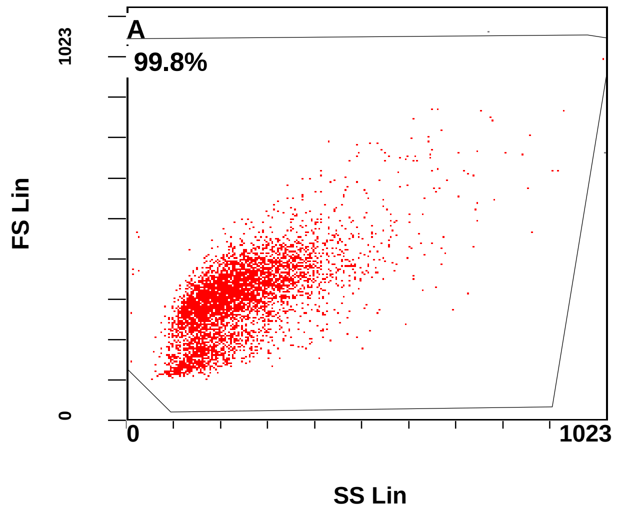

(F1)[A] LO2-50 user2018 00008651 893.LMD : FL3 Log - ADC

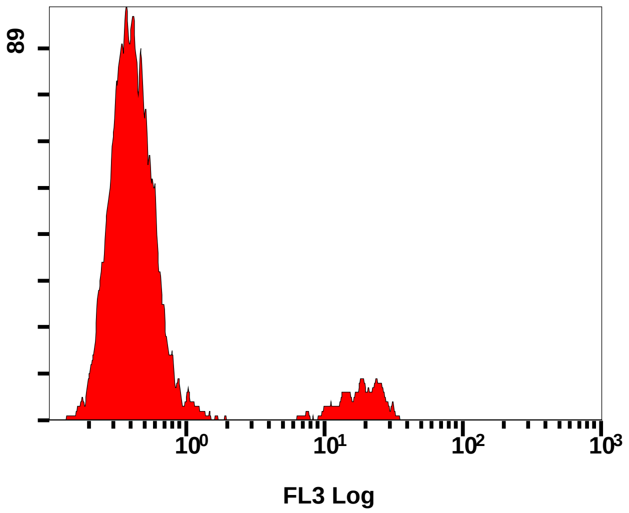

(F1)[A] LO2-50 user2018 00008651 893.LMD : FL1 Log - /

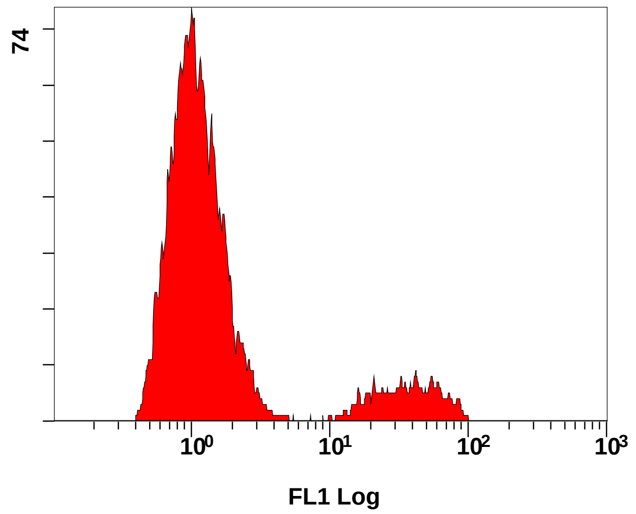

(F1)[A] LO2-50 user2018 00008651 893.LMD : FL1 Log/FL3 Log - ADC

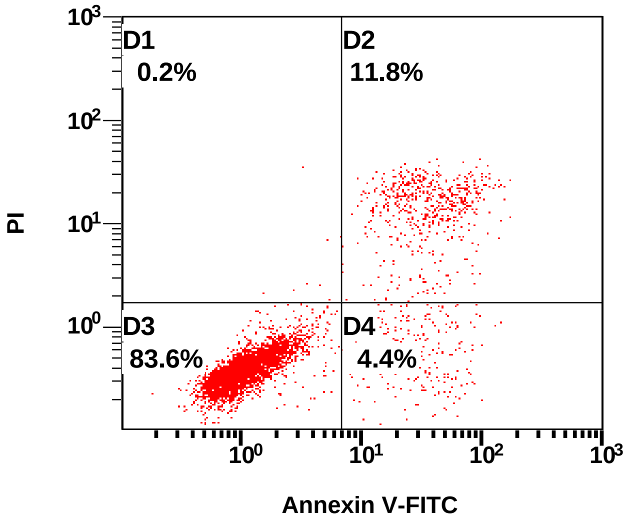

Institution: FC500
Protocol: LO2-50 user2018 00008652 894.PRO
Listmode Replay: New Protocol
Analysis Date: 29-Nov-2019, 15:33:04
Settings File: PC9_Annexin V-Pi.PRO, 19-Nov-2019, 16:13:10
Listmode File: LO2-100 user2018 00008650 892.LMD

Run Date: 19-Nov-19, 16:13:59
Sample ID: LO2-100
User ID: user2018
Acquisition Time/Events: 16.6s / 10000 (PROTOCOL)

Instrument SN: AM18110 Software Version: CXP 2.3

(F1)[Ungated] LO2-100 user2018 00008650 892.LMD : SS Lin/FS l

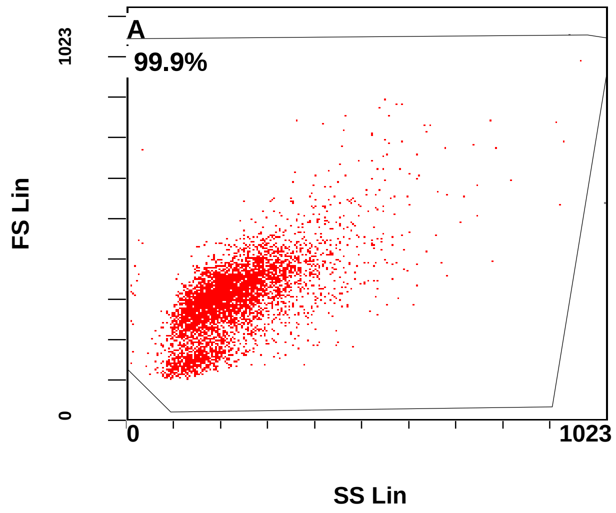

(F1)[A] LO2-100 user2018 00008650 892.LMD : FL3 Log - ADC

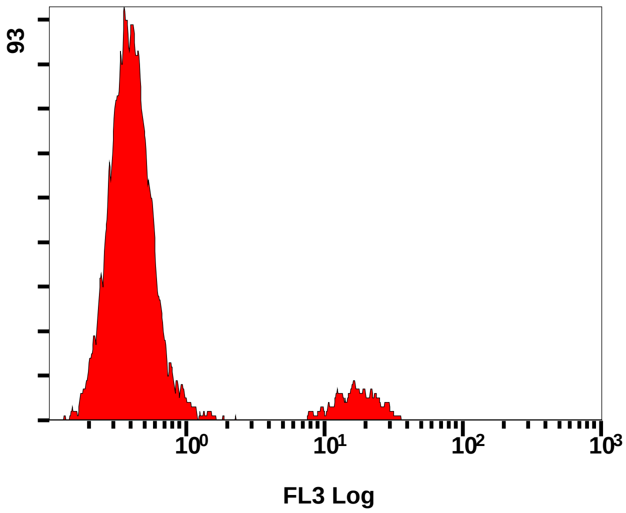

(F1)[A] LO2-100 user2018 00008650 892.LMD : FL1 Log -

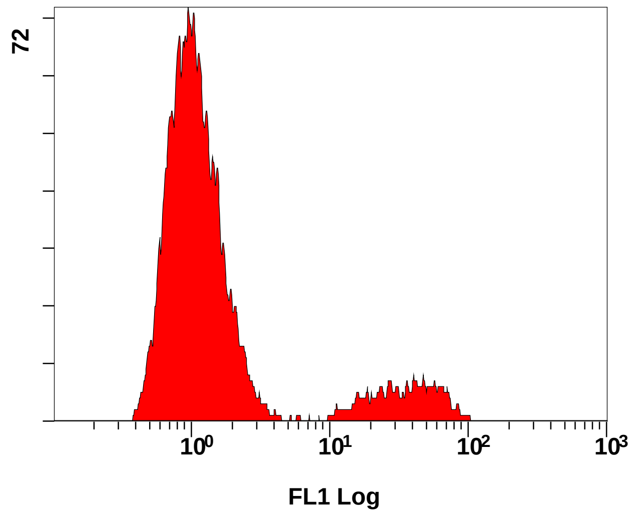

(F1)[A] LO2-100 user2018 00008650 892.LMD : FL1 Log/FL3 Log - ADC

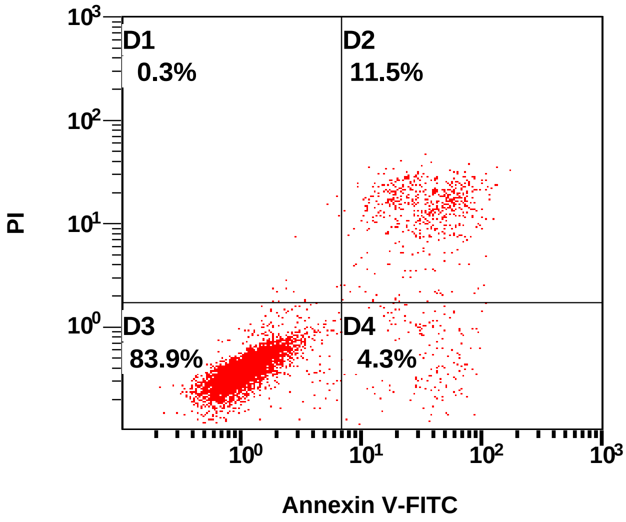

标本1 报告

标本名：标本1　　　　检验时间：2019/12/5 13:05

仪器：NovoCyte Quanteon 622190610647　　　　软件：NovoExpress 1.4.0

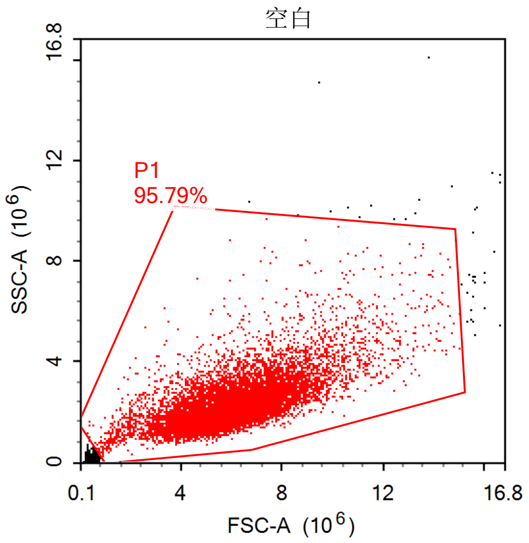

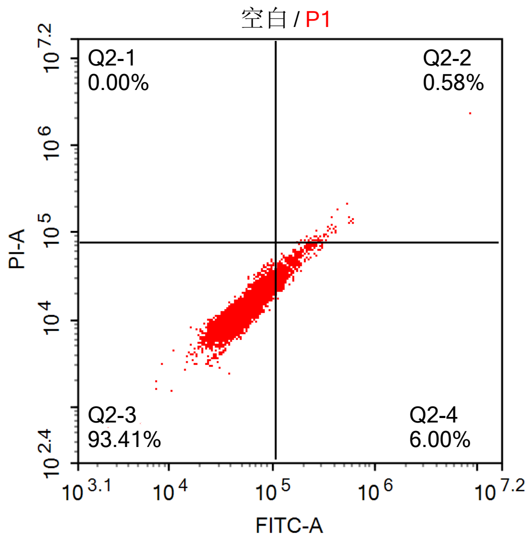

样本统计表格 - 空白

Gate	Count	% Parent	X	Y	Median X	Median Y
All	10,000					
P1	9,579	95.79%	FSC-A	SSC-A	6,015,682	2,233,237
Q2-1	0	0.00%	FITC-A	PI-A	0	0
Q2-2	56	0.58%	FITC-A	PI-A	294,045	90,263
Q2-3	8,948	93.41%	FITC-A	PI-A	53,823	13,921
Q2-4	575	6.00%	FITC-A	PI-A	130,250	37,521

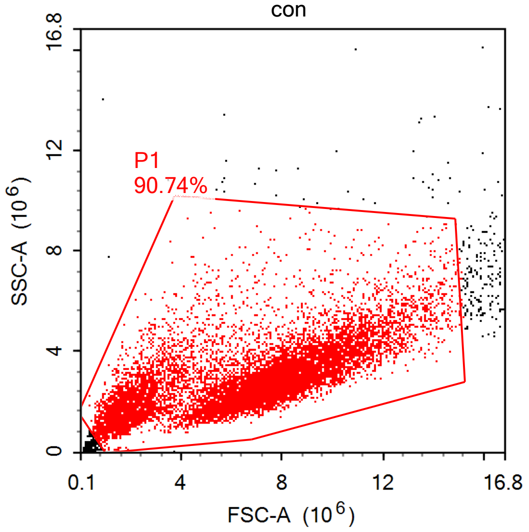

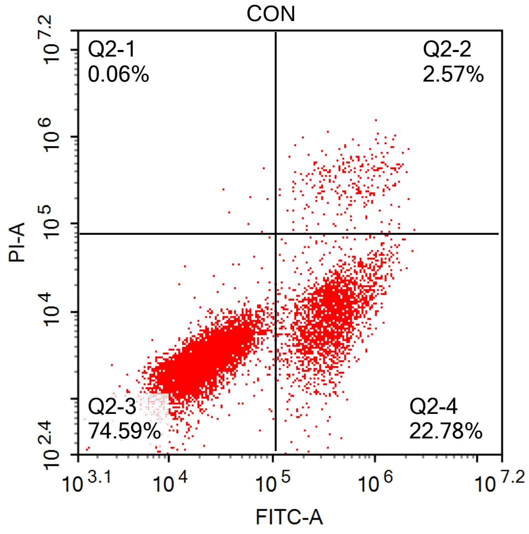

样本统计表格 - con

Gate	Count	% Parent	X	Y	Median X	Median Y
All	11,755					
P1	10,667	90.74%	FSC-A	SSC-A	6,949,314	2,746,007
Q2-1	7	0.06%	FITC-A	PI-A	60,062	195,317
Q2-2	274	2.57%	FITC-A	PI-A	580,584	328,866
Q2-3	7,956	74.59%	FITC-A	PI-A	22,069	2,861
Q2-4	2,430	22.78%	FITC-A	PI-A	368,716	9,315

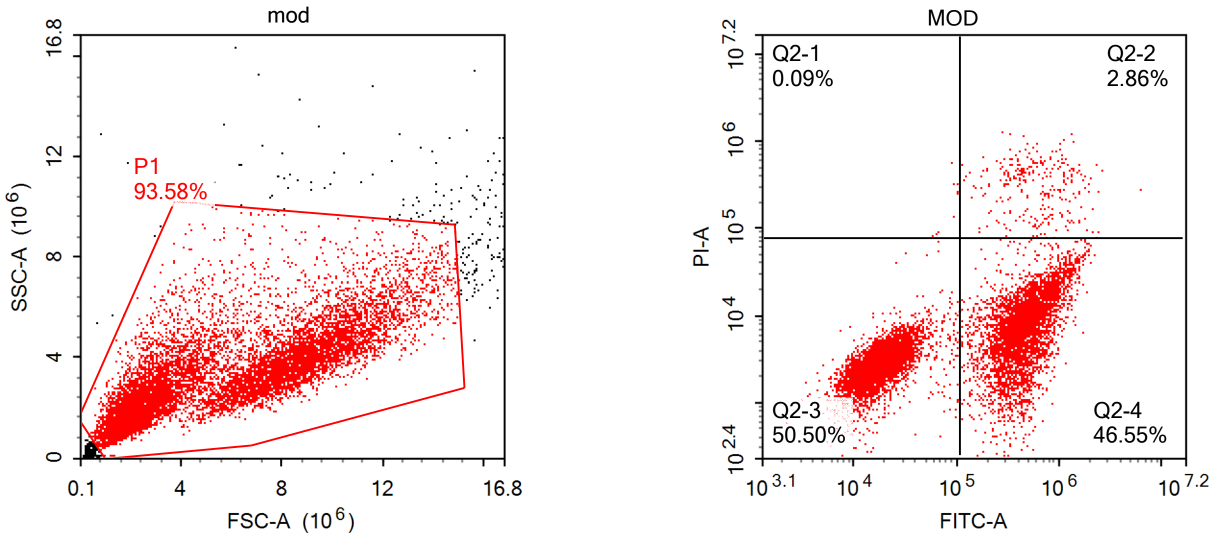

样本统计表格 - mod

Gate	Count	% Parent	X	Y	Median X	Median Y
All	10,686					
P1	10,000	93.58%	FSC-A	SSC-A	5,523,292	3,228,400
Q2-1	9	0.09%	FITC-A	PI-A	93,400	251,878
Q2-2	286	2.86%	FITC-A	PI-A	601,001	359,940
Q2-3	5,050	50.50%	FITC-A	PI-A	17,696	2,753
Q2-4	4,655	46.55%	FITC-A	PI-A	447,161	7,840

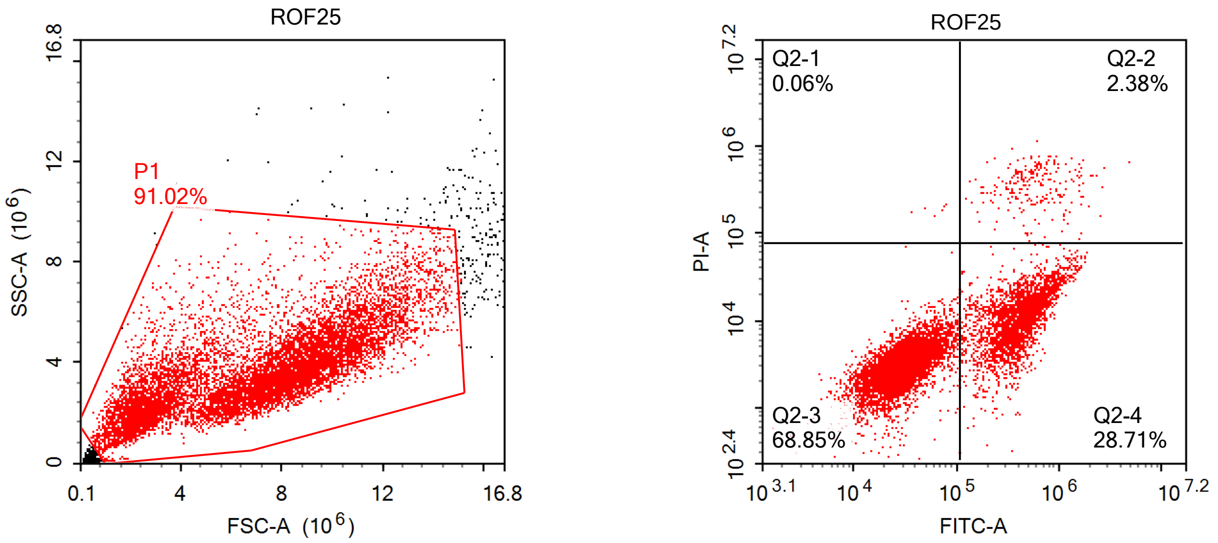

样本统计表格 - ROF25

Gate	Count	% Parent	X	Y	Median X	Median Y
All	10,986					
P1	10,000	91.02%	FSC-A	SSC-A	7,368,979	3,472,919
Q2-1	6	0.06%	FITC-A	PI-A	65,896	187,018
Q2-2	238	2.38%	FITC-A	PI-A	563,421	375,826
Q2-3	6,885	68.85%	FITC-A	PI-A	29,442	3,088
Q2-4	2,871	28.71%	FITC-A	PI-A	405,093	11,118

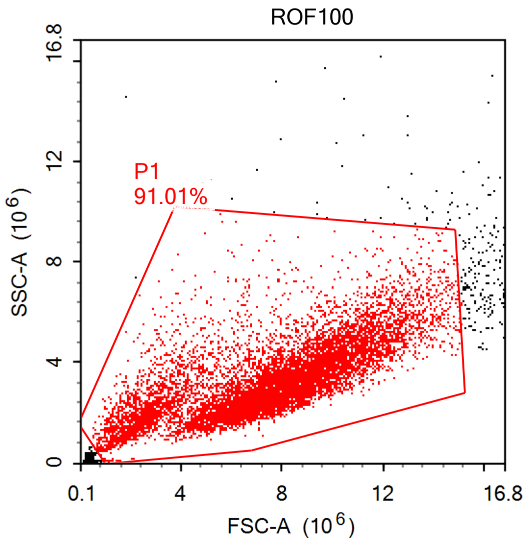

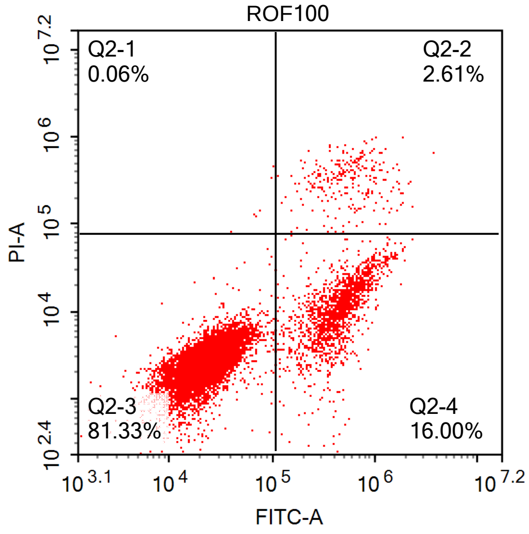

样本统计表格 - ROF100

Gate	Count	% Parent	X	Y	Median X	Median Y
All	10,988					
P1	10,000	91.01%	FSC-A	SSC-A	7,703,932	3,122,484
Q2-1	6	0.06%	FITC-A	PI-A	71,526	137,388
Q2-2	261	2.61%	FITC-A	PI-A	536,729	350,078
Q2-3	8,133	81.33%	FITC-A	PI-A	22,213	2,645
Q2-4	1,600	16.00%	FITC-A	PI-A	435,811	10,773

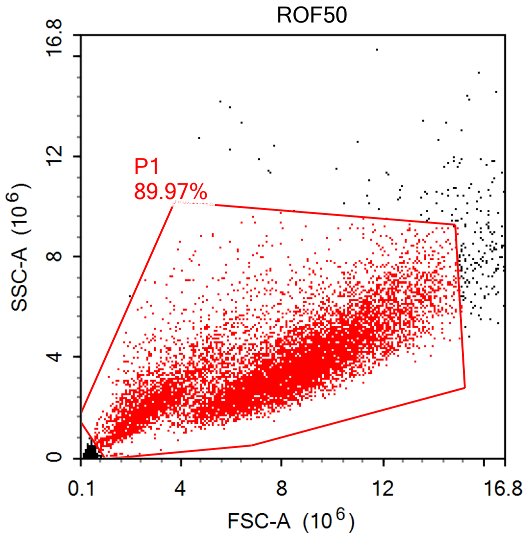

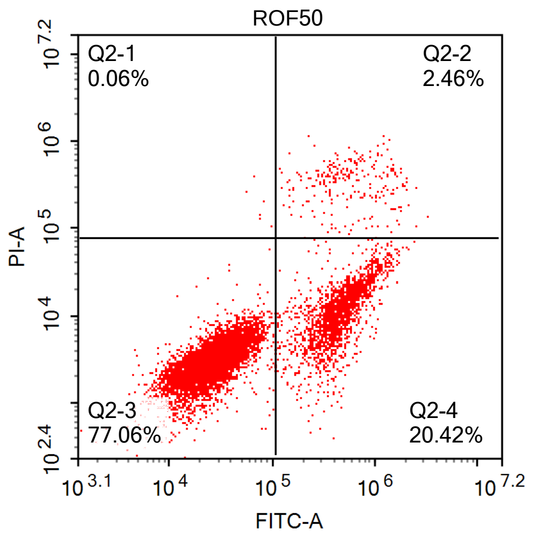

样本统计表格 - ROF50

Gate	Count	% Parent	X	Y	Median X	Median Y
All	11,115					
P1	10,000	89.97%	FSC-A	SSC-A	7,875,686	3,476,606
Q2-1	6	0.06%	FITC-A	PI-A	75,701	174,744
Q2-2	246	2.46%	FITC-A	PI-A	540,603	371,285
Q2-3	7,706	77.06%	FITC-A	PI-A	26,291	3,126
Q2-4	2,042	20.42%	FITC-A	PI-A	458,609	12,447

EDITSPRINGS

EDITORIAL CERTIFICATE

This is to certify that the manuscript detailed below was edited by one or more of our highly qualified, native English speakers at EditSprings, to assure compliance with Anglophone academic standards in terms of style, punctuation, grammar, and spelling.

Manuscript title:

Rhoifolin Alleviates Alcoholic Liver Disease in vivo and in vitro via Inhibition of the TLR4/NF-κB Signaling Pathway

Authors:

Mai Baoyu

Date Issued:

Mar 27 2022

Certificate Number:

ES-202203201651366647

This certificate can be verified on https://www.editsprings.cn/QueryCertificate.html EditSprings hereby certifies that neither content nor the author's intentions were altered in any way during the editing process. Documents in receipt of this certification should be ready for publication as far as style and language are concerned, provided that the author(s) accepted our suggestions and changes (which remains their right and responsibility).

EditSprings offers a wide range of editing, translation, for researchers and publishers across the world. Our highly skilled editors are all established academics based in Anglophone Higher Education institutions across the world (U.K., U.S.A., Canada, Australia, and elsewhere), are experts in their respective fields, and are qualified to edit research papers authored by non-Anglophone scholars.
