# Supplementary figures and images for: Rhoifolin Alleviates Alcoholic Liver Disease In Vivo and In Vitro via Inhibition of the TLR4/NF-κB Signaling Pathway
Source: Front Pharmacol. 2022 May 24;13:878898. doi: 10.3389/fphar.2022.878898 (PMC9171502; doi:10.3389/fphar.2022.878898)

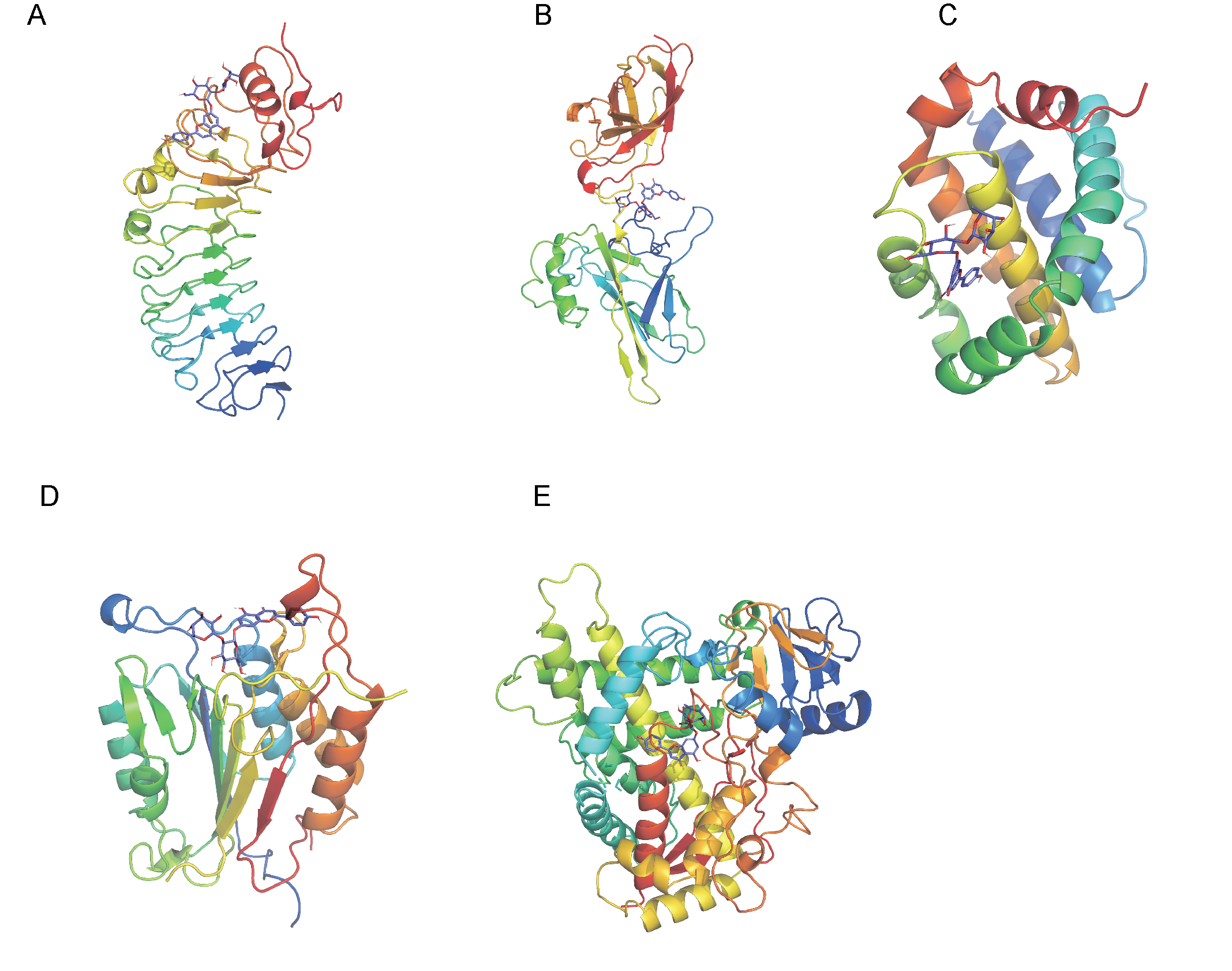

Supplement: Supplementary file 2 [file Image3.tif]

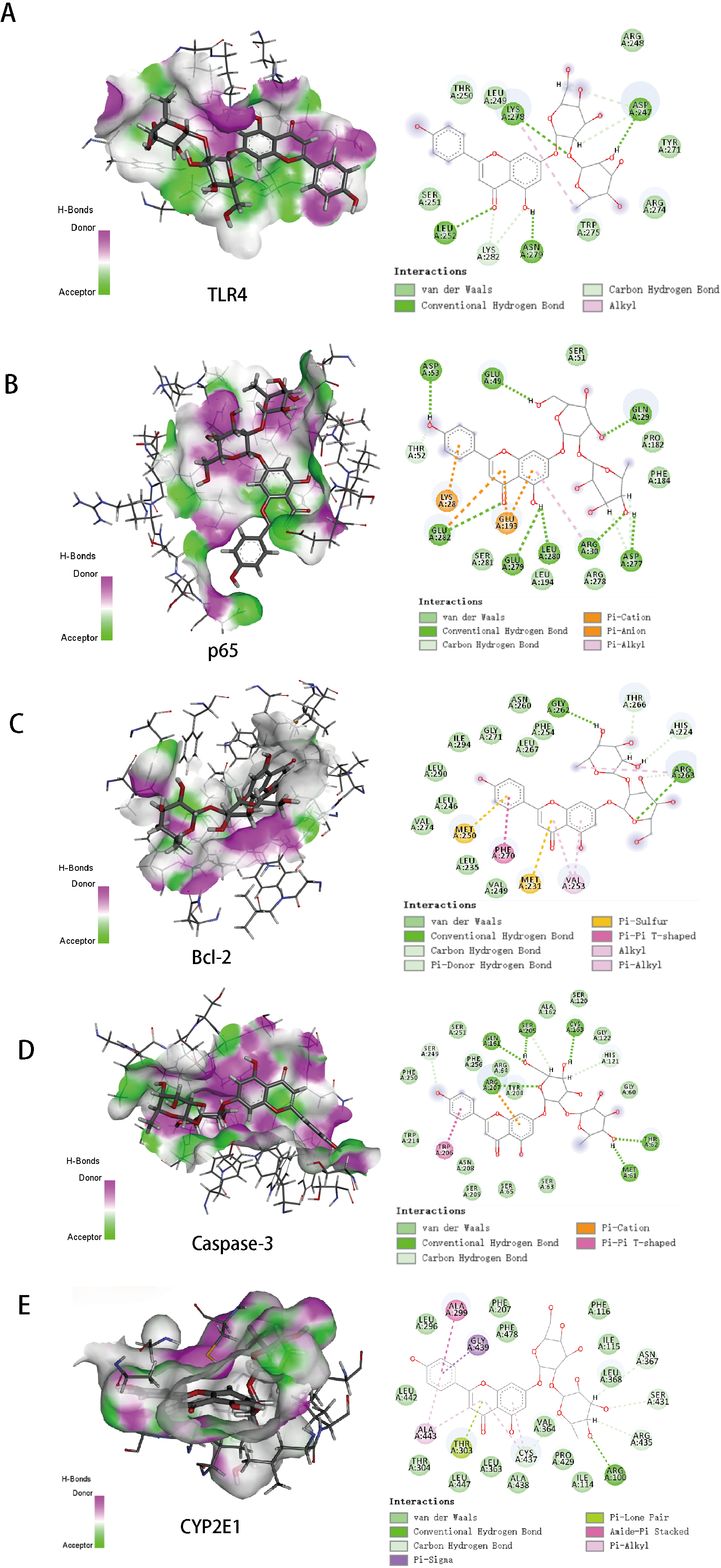

Supplement: Supplementary file 4 [file Image2.tif]

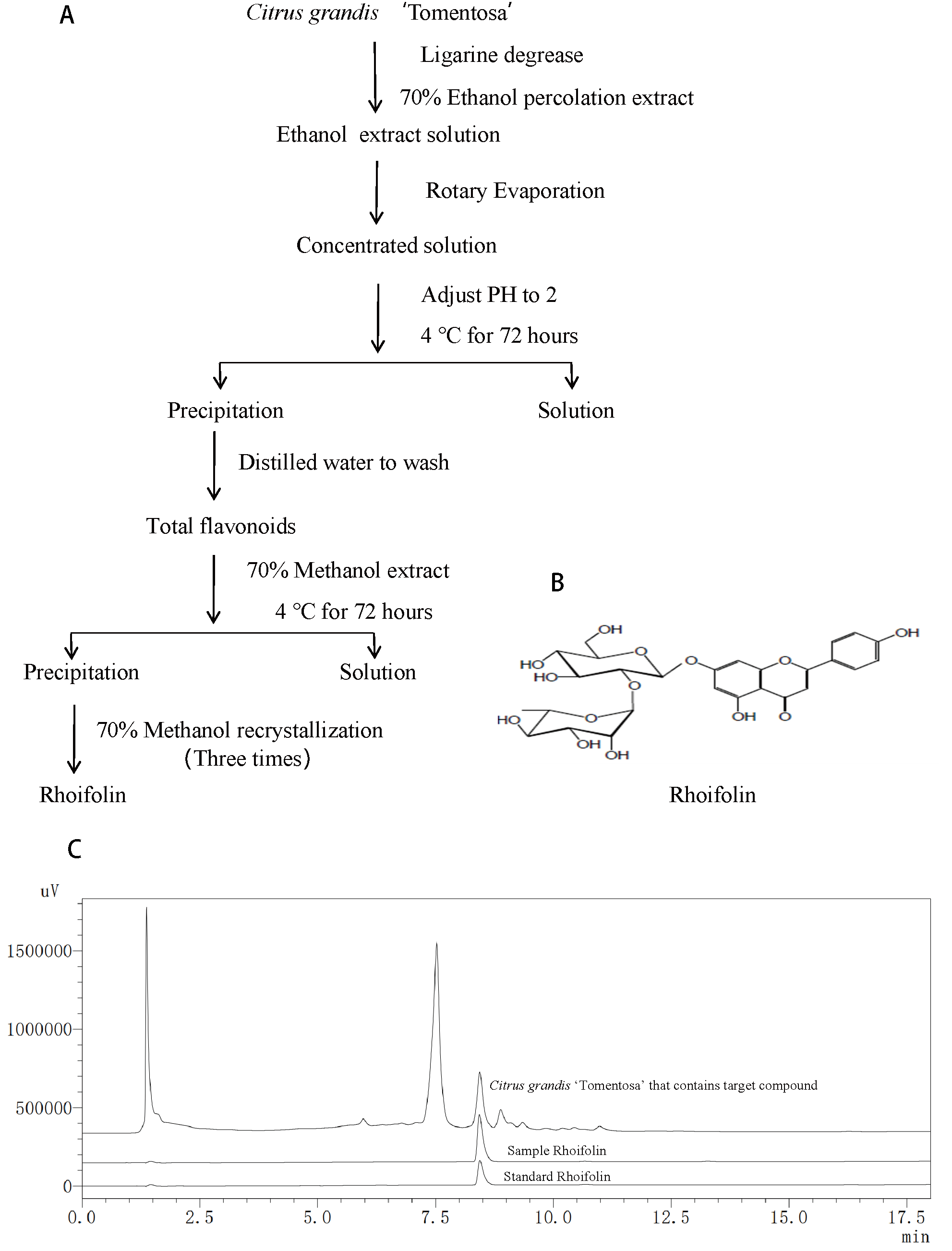

Supplement: Supplementary file 5 [file Image1.tif]
