# Supplementary material for: Rhoifolin Alleviates Alcoholic Liver Disease In Vivo and In Vitro via Inhibition of the TLR4/NF-κB Signaling Pathway
Source: Front Pharmacol. 2022 May 24;13:878898. doi: 10.3389/fphar.2022.878898 (PMC9171502; doi:10.3389/fphar.2022.878898)
Supplement: Supplementary file 6 [file DataSheet1.pdf]

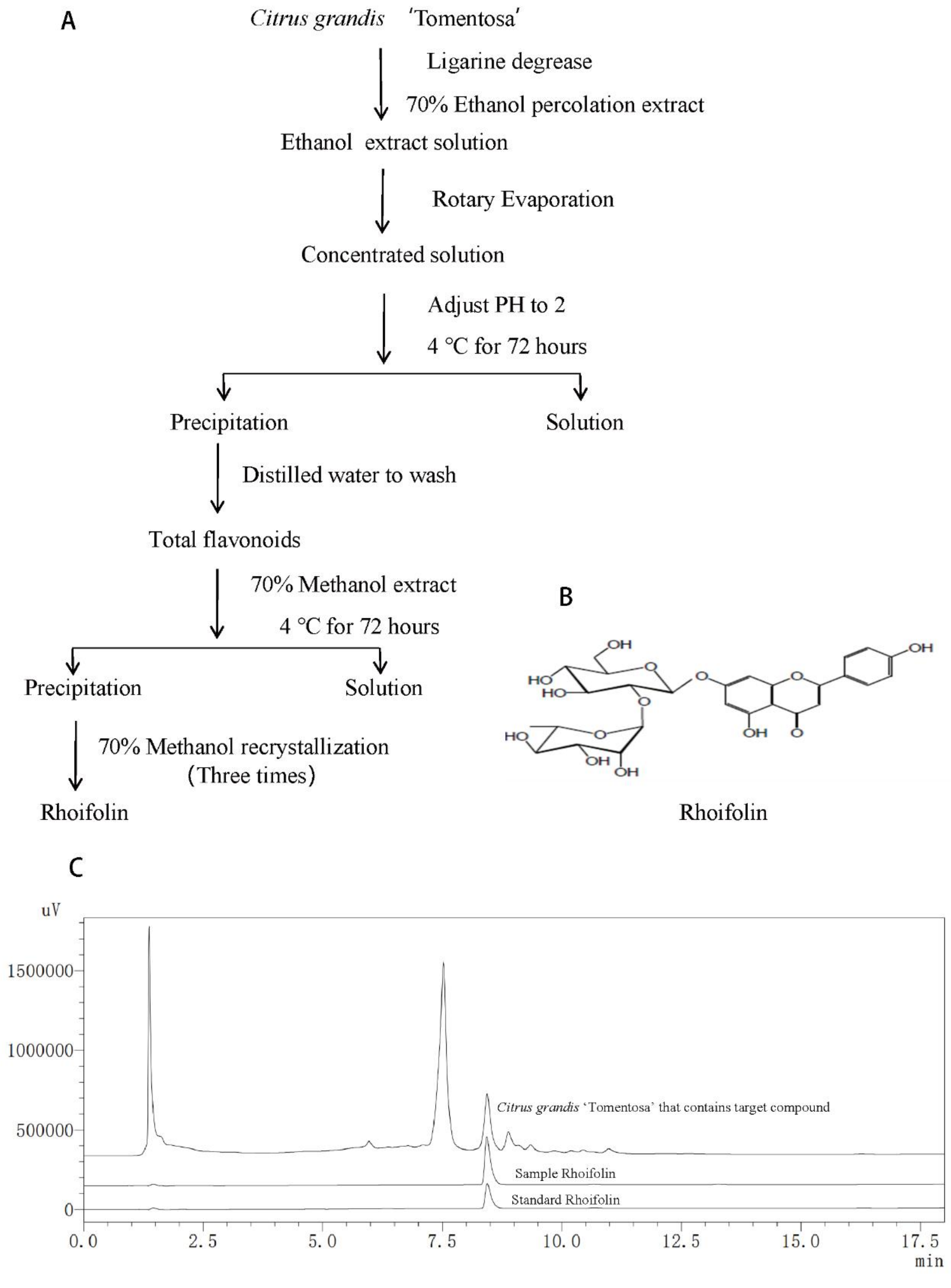

Supplementary Figure 1. Isolation of ROF from *Citrus grandis* 'Tomentosa': The flow chart of extraction and isolation of ROF (A), the chemical structure of ROF (B), and the Chromatograms of a standard ROF, sample ROF and *Citrus grandis* 'Tomentosa' that contains target compound (254 nm) (C).

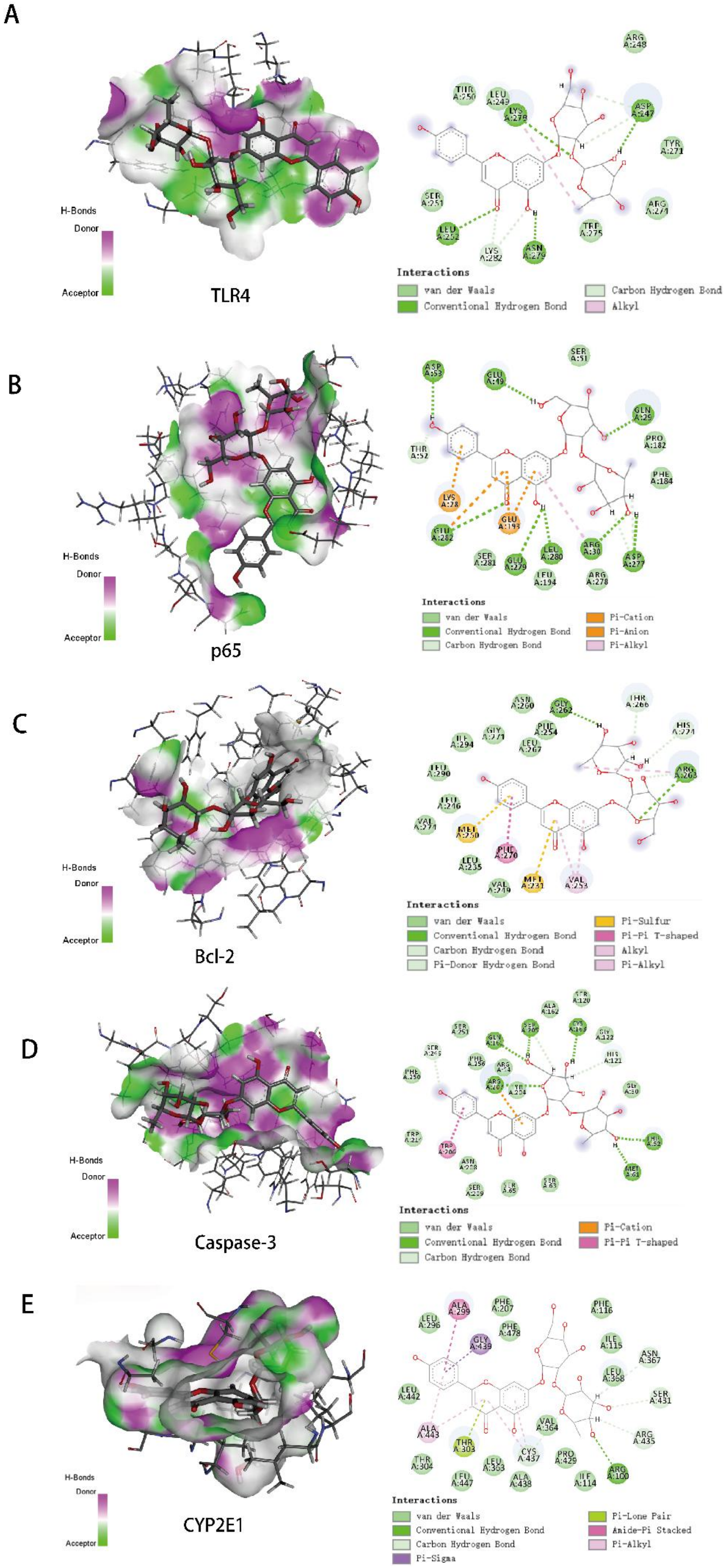

A
H-Bonds
Donor
Acceptor
TLR4
Interactions
van der Waals
Conventional Hydrogen Bond
Carbon Hydrogen Bond
Alkyl
B
H-Bonds
Donor
Acceptor
p65
Interactions
van der Waals
Conventional Hydrogen Bond
Carbon Hydrogen Bond
Pi-Cation
Pi-Anion
Pi-Alkyl
C
H-Bonds
Donor
Acceptor
Bcl-2
Interactions
van der Waals
Conventional Hydrogen Bond
Carbon Hydrogen Bond
Pi-Donor Hydrogen Bond
Pi-Sulfur
Pi-Pi T-shaped
Alkyl
Pi-Alkyl
D
H-Bonds
Donor
Acceptor
Caspase-3
Interactions
van der Waals
Conventional Hydrogen Bond
Carbon Hydrogen Bond
Pi-Cation
Pi-Pi T-shaped
E
H-Bonds
Donor
Acceptor
CYP2E1
Interactions
van der Waals
Conventional Hydrogen Bond
Carbon Hydrogen Bond
Pi-Sigma
Pi-Lone Pair
Amide-Pi Stacked
Pi-Alkyl

Supplementary Figure 2. Bioactive compound–protein docking combination: (A)TLR4, (B)p65, (C)Bcl-2, (D)Caspase-3, (E) CYP2E1.

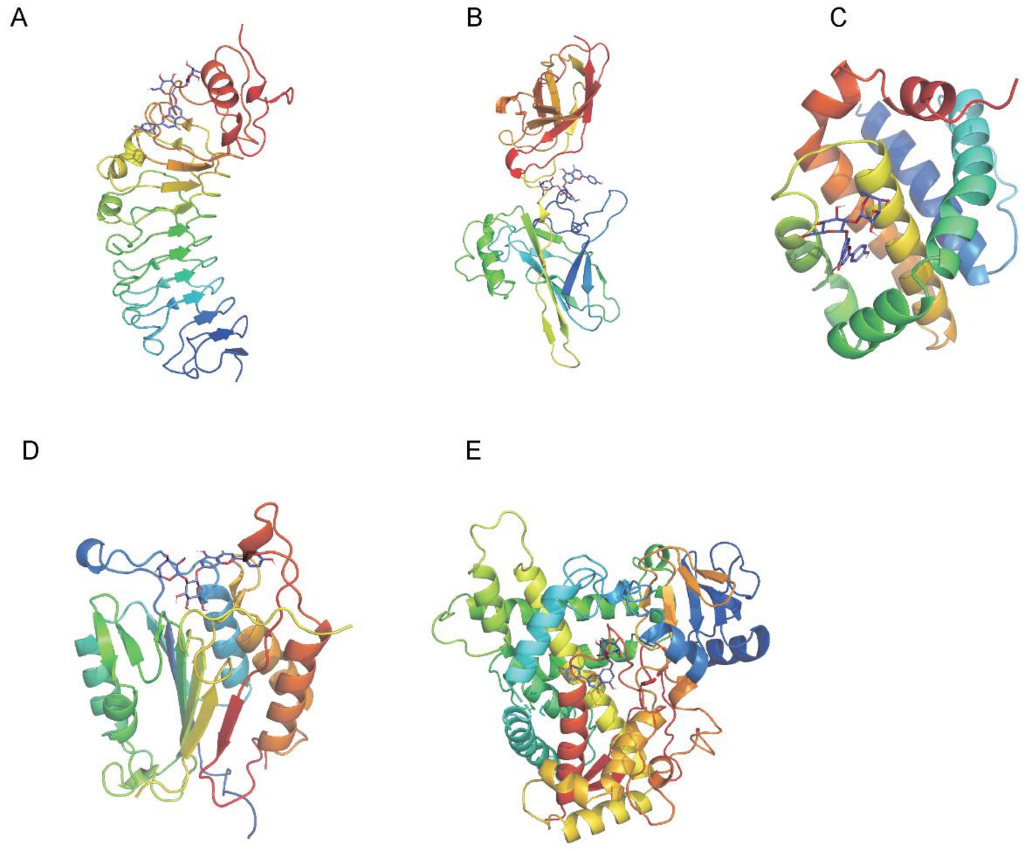

Supplementary Figure 3. Bioactive compound–protein docking combination (3D binding structure): (A)TLR4, (B)p65, (C)Bcl-2, (D)Caspase-3, (E) CYP2E1.

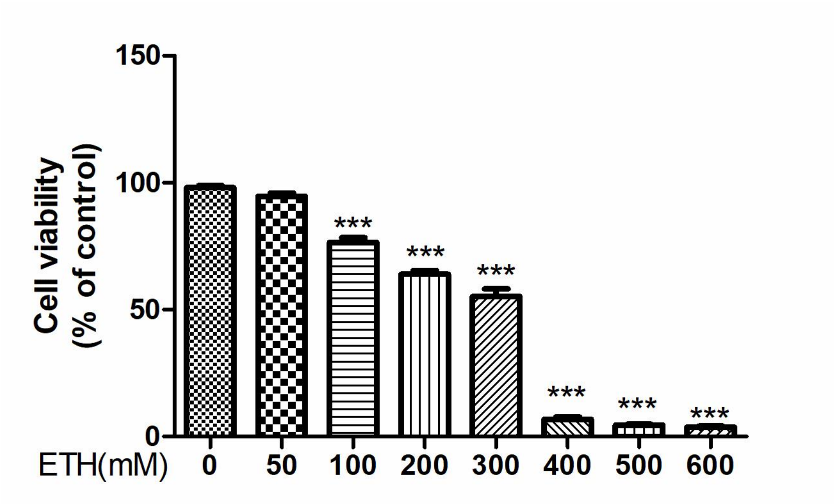

Supplementary Figure 4. Effects of ethanol on cell apoptosis and viability. Mean ± SEM (n=6). ***P<0.001 vs. the CON group.
